# Classification of Atmospheric Traveling Waves at Cloud Level

**DOI:** 10.1007/s00205-026-02253-w

**Published:** 2026-10-18

**Authors:** Adrian Constantin, Zhiwu Lin, Hao Zhu

**Affiliations:** 1 Faculty of Mathematics, University of Vienna, Oskar-Morgenstern-Platz 1, 1090 Vienna, Austria; 2 School of Mathematical Sciences, Fudan University, 200433 Shanghai, People’s Republic of China; 3 School of Mathematics, Nanjing University, 210093 Nanjing, Jiangsu, People’s Republic of China

## Abstract

We classify within the quasi-geostrophic framework all types of traveling waves in zonal bands of the planetary atmosphere at cloud level according to their wave speeds. This classification pertains to waves of all amplitudes, going beyond the small-amplitude perturbative regime; it provides a structurally robust criterion for determining which traveling-wave profiles are dynamically possible. We also show that each wave classification type was observed on Jupiter or Saturn. Building on this classification, we investigate the related rigidity issue for large-amplitude traveling waves and waves propagating near shear flows. Our study offers a unified quantitative characterization of the intrinsic constraints for traveling waves in the quasi-geostrophic regime of planetary atmospheric flow.

## Introduction

The two-dimensional incompressible β-plane equation is widely used to study the dynamics of quasi-geostrophic flows that are confined to zonal bands and for which the vertical motion is negligible. Such flows are predominant in the dynamics of the atmosphere of the gas giants (Jupiter and Saturn) and of the ice giants (Neptune and Uranus) at cloud level. By approximating the Coriolis parameter as varying linearly with latitude, having the form $f_0+\beta y$ with

1.1 $$\begin{aligned} f_0=\frac{2\varOmega 'R'}{U'}\,\sin \theta _0\quad \text {and}\quad \beta =\frac{2\varOmega 'R'}{U'}\,\cos \theta _0\,, \end{aligned}$$

the β-plane equation captures the essential behavior induced by sphericity combined with rotation; here $\Omega^{\prime }$ is the constant rate of rotation of the spherical planet of radius $R^{\prime }$ about its polar axis, $U^{\prime }$ is the typical magnitude of the horizontal velocity and $$\theta _0 \in \big ( -\frac{\pi }{2},\,\frac{\pi }{2}\big )$$ is the reference latitude of the flow region that does not comprise the poles (see the discussion in [10, 30] and Table 1). Rather than the more intricate Euler equation in rotating spherical coordinates, the β-plane equation is formulated in a Cartesian coordinate system, which simplifies the analysis but retains the effects of sphericity at leading order for the bands of moderate meridional width typical for Jupiter and Saturn [48]. The atmospheric flow at cloud level on Neptune and Uranus has also a zonal structure but the bands are much wider than those on Jupiter and Saturn: both ice giants have only two major prograde jets, one per hemisphere, with retrograde equatorial jets [45]. Moreover, the latitudinally averaged zonal flow is stable on Neptune and Uranus (see [6]), a feature which impedes the formation of long-lasting wave perturbations, in marked contrast to the gas giants.

**Table 1:** Values of the relevant parameters for Jupiter and Saturn

Planet	$R^{\prime }$	$\Omega^{\prime }$	$U^{\prime }$
Jupiter	69911 km	$1.76\times 10^{-4}$ rad/s	150 m/s
Saturn	58232 km	$1.62\times 10^{-4}$ rad/s	150 m/s

The banded appearance of Jupiter and Saturn is primarily caused by their rapid rotation. Both giant planets exhibit east–west jet streams along the boundaries of these zonal bands, with speeds ranging up to 150 m s$^{-1}$ on Jupiter and up to 400 m s$^{-1}$ on Saturn. These jets dominate the dynamics, containing most of the kinetic energy at the cloud tops on Saturn and about 90% on Jupiter [41], and lead to the formation of zonally propagating long-lived coherent shear flows. A plethora of short-lived cloud structures arise as perturbations of these shear flows but some persistent dynamical features can also be observed. Typically these are traveling waves propagating zonally but occasionally vortices, such as Jupiter’s Great Red Spot (GRS) and Saturn’s polar vortices, also occur. While vortices that persist on large time scales require special circumstances (for example, the GRS has a quite peculiar dynamics unlike that of terrestrial hurricanes [8] and Saturn’s polar vortices are hot spots [42]), a wide range of traveling waves have been identified by telescope and spacecraft (see [39] for the identification of a variety of waves detected on Jupiter by the Juno spacecraft). Such waves are unstable perturbations of an underlying shear flow that grow and acquire specific dynamic characteristics which set them apart from the original state of the flow (e.g. the circumpolar waves that form Saturn’s hexagon – see [9]).

Coherent waves can only propagate azimuthally since the strong jets ensure that waves propagating in a direction tilted to the East–West direction are short-lived. The velocity field of a traveling wave propagating zonally in a band $d_- \le y \le d_+$ at the speed *c* has the form (u(x-ct,y),v(x-ct,y)) with v=0 on $y=d_\pm$. Genuine waves correspond to flows which depend on the variable (x-ct), while an incompressible shear flow is described by a velocity of the form (*U*(*y*), 0). Within the framework of linear theory (see [34, 36]), shear flow perturbations corresponding to the stream function $\psi (x,y,t)=\phi (y)\textrm{e}^{\textrm{i}\alpha (x-ct)}$, where α>0 is the wave-number in the *x*-direction and *c* is the complex wave speed, are governed by the Rayleigh-Kuo equation

1.2 $$\begin{aligned} (U-c) (\alpha ^2\phi -\phi '') - (\beta -U'')\phi =0, \end{aligned}$$

with the boundary conditions $$\phi (d_-)=\phi (d_+)=0$$. Equation (1.2) has a singularity at a critical layer (a point $$y_c$$ with $$U(y_c)=c$$), since the coefficient of the highest-order derivative vanishes. In this case a perturbation analysis shows the appearance of vortices near the critical layer. In the absence of critical layers, instability can only arise if the imaginary part of the speed *c*, giving the growth rate of the perturbation, does not vanish. This leads to the condition that $\beta -U^{\prime \prime }$ vanishes at some level as being necessary for linear instability (see [14]). In light of these classical results, we expect that genuine traveling waves can arise as coherent perturbations of a shear flow only under special circumstances. This perspective is supported by recent rigidity results showing that, under suitable conditions, certain shear flows do not admit any nontrivial traveling-wave perturbations [10, 20].

From the observational viewpoint, the four giant planets exhibit markedly different amounts of large-scale coherent wave activity. Several long-lived wave patterns have been observed on Jupiter and Saturn (some will be described below), but Uranus is the most featureless of the giant planets and Neptune’s most notable visible flow patterns are its dark spots. The very wide zonal bands on Neptune do not confine the dark spots tightly: for example, the largest, Neptune’s Great Dark Spot, first observed by Voyager 2 in 1989, drifted towards the equator and disappeared by 1994 (see [27]). On the other hand, on Jupiter and Saturn one finds a profusion of coherent waves: Saturn’s stationary hexagon, meandering ribbons, Jupiter’s chevrons and band-limited wave trains, as well as more intricate wave patterns surrounding the GRS.

The main aim of this paper is to provide a *complete and rigorous classification* of genuine traveling-wave solutions arising as coherent flow patterns with possibly large meridional velocity components and thus not necessarily representing small perturbations of a shear flow in a zonal band. More precisely, we consider the β-plane equation

1.3 $$\begin{aligned} {\left\{ \begin{array}{ll} u_t + uu_x+vu_y - (f_0+\beta y)\, v = - {\mathfrak {P}}_x\,,\\ v_t + uv_x+vv_y + (f_0+\beta y) \, u = - {\mathfrak {P}}_y\,,\\ u_x + v_y=0 \end{array}\right. } \end{aligned}$$

confined to a zonal band ${\mathfrak {D}}_L=\{(x,y):\ x \in {\mathbb {T}}_L={\mathbb {R}}/_{L{\mathbb {Z}}},\ y \in [d_-,d_+]\}$, where (*u*, *v*) is the horizontal velocity field, ${\mathfrak {P}}$ is the pressure, and $f_0,\beta$ are defined in (1.1). The associated non-permeable boundary condition is

1.4 $$\begin{aligned} v=0\quad \text {on}\quad y = d_\pm \,. \end{aligned}$$

We will prove the following classification theorem:

### Theorem 1.1

Given L>0 and $d_+>d_-$, let

$$\begin{aligned} (u(x-ct,y),v(x-ct,y))\in C^2({\mathfrak {D}}_L) \end{aligned}$$

be an *L*-periodic genuine traveling-wave solution to the β-plane equation (1.3)-(1.4).

(i) If β>0, then the wave speed *c* must fall into one of the following four categories:

1. *c* is a generalized inflection value of *u*, i.e. $\{\beta -\Delta u=0\}\cap \{u=c\}\ne \emptyset$;
2. *c* is a critical value of *u*, i.e. $\{\nabla u=0\}\cap \{u=c\}\ne \emptyset$;
3. *c* is a maximum or minimum of *u*;
4. *c* is outside $Ran(u)=[u_{\min },u_{\max }]$, specifically, $c\in [c_\beta ^+,u_{\min })$ and there exists an integer $n_0\ge 1$ such that $\Lambda _{n_0}(a)=\beta$ holds for a=c, where
  1.5 $$\begin{aligned} c^{+}_{\beta }=u_{\min }-\frac{\beta (d_+-d_-)^2}{2\pi ^2}-\frac{(d_+-d_-)^2}{2\pi ^2}\sqrt{\beta ^2+{4\pi ^2\beta \over (d_+-d_-)^2}(u_{\max }-u_{\min })} \end{aligned}$$
  and
  1.6 $$\begin{aligned} \Lambda _{n_0}(a)=\sup _{V\subset H_0^1({\mathfrak {D}}_L),\dim V=n_0-1}\,\inf _{G\perp V, G\in H_0^1({\mathfrak {D}}_L),G\ne 0}{\int _{{\mathfrak {D}}_L}(u-a)^2|\nabla G|^2dxdy\over \int _{{\mathfrak {D}}_L} (u-a)G^2dxdy}. \end{aligned}$$

(ii) If β=0 (*f*-plane approximation), then *c* must be a generalized inflection value of *u*.

In the context of Theorem 1.1, we denoted that

$$\begin{aligned} f_{\max }=\max _{(x,y)\in {\mathfrak {D}}_L}\{f(x,y)\}\quad \text {and}\quad f_{\min }=\min _{(x,y)\in {\mathfrak {D}}_L}\{f(x,y)\}\, \end{aligned}$$

for a function $f \in C({\mathfrak {D}}_L)$. A genuine traveling wave is a periodic flow propagating in the East–West direction and presenting meridional variations, whereas flows with v≡0 throughout ${\mathfrak {D}}_L$ are termed shear flows – by the third equation in (1.3), shear flows depend only on the *y*-variable. The boundary condition (1.4) expresses the presence of zonal jets along it, confining the wave to the respective zonal band. Field data shows that the zonal jet systems of Jupiter and Saturn reach below the troposphere and have remained practically unchanged since the beginning of detailed spacecraft observations (see [17]). In addition to these zonal jets, some peculiar flow patterns occur: unlike the majority of wind jets on Jupiter and Saturn, near 47$^\circ$N on Saturn, wavy perturbations of a jet produce a long-lived meandering ribbon (see Fig. 2) – a similar ribbon on Jupiter at 30$^\circ$N was rather short-lived. Let us also note that

$$\begin{aligned} \Gamma =v_x-u_y+ \beta y \end{aligned}$$

is the total vorticity, comprising two distinct components: the relative vorticity $\gamma =v_x-u_y$ due to the fluid motion and the planetary vorticity βy due solely to the planet’s rotation about its polar axis (this being the β-plane correspondent of the concepts in spherical coordinates, discussed in [7]). By the third equation in (1.3), expressing mass conservation, we have $\Gamma _y=\beta -\Delta u$, which provides another perspective for the interpretation of generalized inflection points as stationary points of the variation of the total vorticity with latitude.

**Fig. 1:**
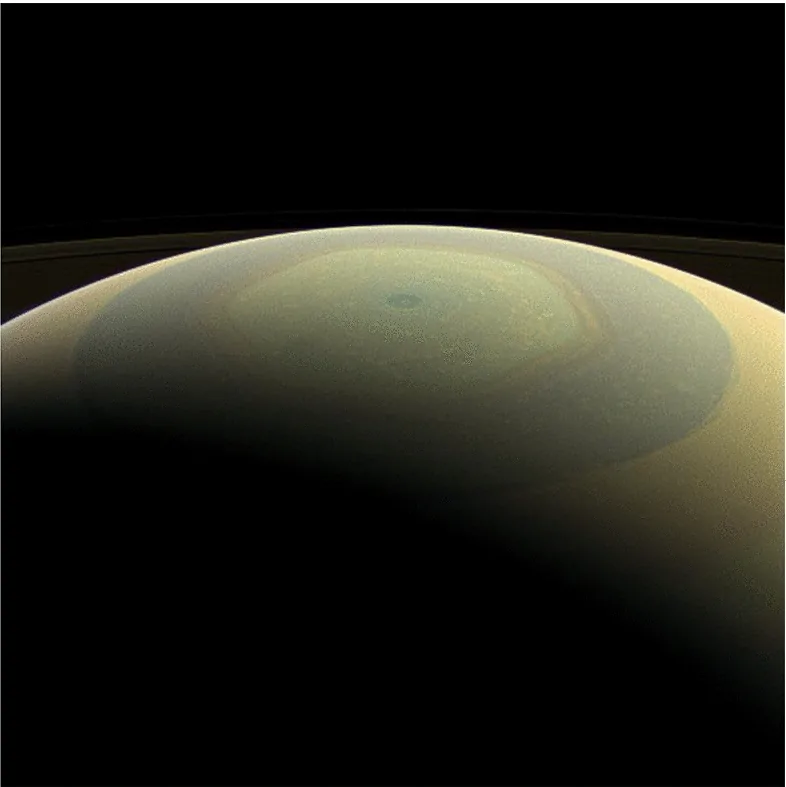
Natural-color view of Saturn’s hexagon, taken with the Cassini spacecraft wide-angle camera on 22 July 2013 (credit: NASA/JPL-Caltech/Space Science Institute). The stationary hexagonal band between 72$^\circ$N-78$^\circ$N, appearing somewhat yellow, has persisted dynamically since its discovery by Voyager 2 in 1981 [9]. Only the hexagon’s color changes seasonally, alternating between turquoise and yellow – during Saturn’s summer (lasting for about 7.5 Earth-years), sunlight triggers the formation of photochemical hazes, which give the planet’s atmosphere a yellow hue

**Fig. 2:**
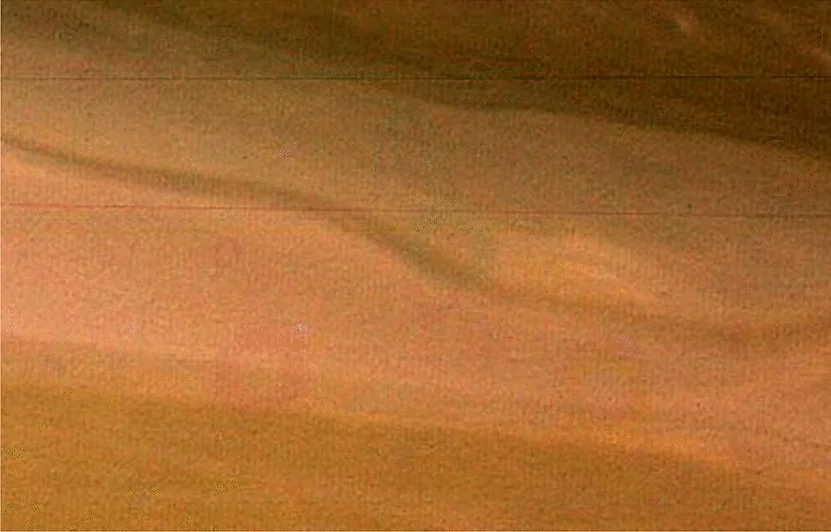
Voyager 2 image of Saturn’s ribbon, captured on 23 August 1981 (credit: NASA/JPL-Caltech/Space Science Institute). North is to the upper right and the wave is the dark meandering streak in the light band – in the image the eastward direction of wave propagation (along 47$^\circ$N) is inclined downwards. Since its discovery, this wave has been seen in every high-resolution image of Saturn that covers northern mid-latitudes [17]. The ribbon’s oscillation amplitude is about 2 degrees of latitude from peak to trough, matching the width of the jet it resides in

**Fig. 3:**
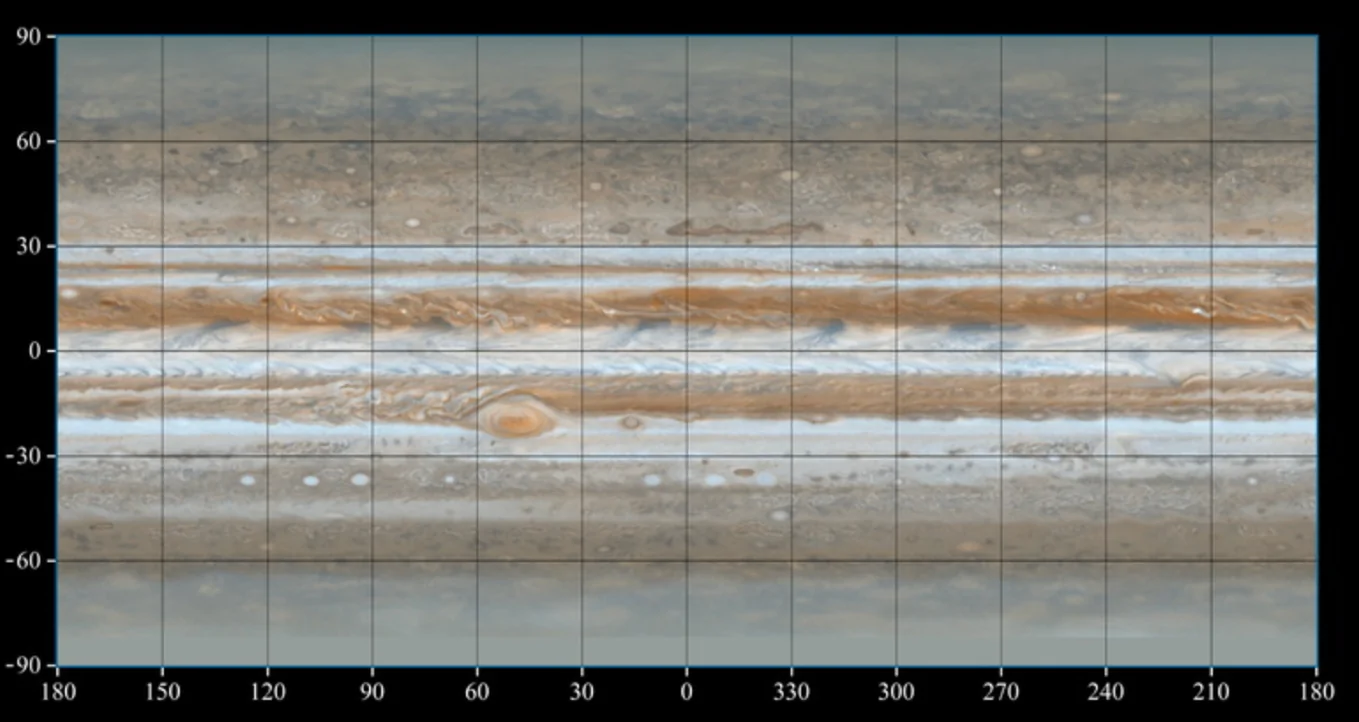
Color map of Jupiter in cylindrical coordinates, obtained from images taken by the camera onboard NASA’s Cassini spacecraft on 11–12 December 2000 (credit: NASA). Jupiter’s GRS is confined to a zone delimited by a westward jet at 19.5$^\circ$S with speed 70 m s$^{-1}$ and an eastward jet at 26.5$^\circ$S with speed 50 m s$^{-1}$, chevrons propagating along the latitude 7.5$^\circ$S can be clearly distinguished eastwards of the GRS and Jupiter’s ribbon is the whitish wave pattern visible along the latitude 30$^\circ$N

By means of available data for wave patterns on Jupiter and Saturn observed by spacecraft or Earth-based telescopes and interpreted within the regime of the β-plane approximation, we show that each classification type of large-scale coherent waves from Theorem 1.1 occurs on the gas giants:

- Saturn’s hexagon (see Fig. 1) illustrates type (i1) since for the stationary circumpolar wave (with speed c=0) embedded within the hexagonal strip near the center of the hexagonal band at 75$^\circ$N, we have u=0 and $\beta -u_{yy}=0$ at this latitude according to the data gathered in Fig. 3 of [47].
- Saturn’s ribbon (see Fig. 2) illustrates type (i2), with a wave speed of 150 m s$^{-1}$ matching the speed of the jet at 47$^\circ$N (see [43]). Since 47$^\circ$N is a line of zero relative vorticity (see [43]), taking the mean value of $v_x-u_y=0$ over a period in the *x*-variable yields locations where $u_y=0$ at this latitude. Furthermore, u=c implies $u_x=0$ along 47$^\circ$N, so that at these locations we find critical points of *u*.
- Jupiter’s ribbon at 30$^\circ$N and Jupiter’s chevrons at 7.5$^\circ$S (see Fig. 3) are examples of type (i3) waves. Observations with the Hubble Space Telescope in the period between 1994 and 2008 revealed that Jupiter’s ribbon consists of wavy oscillations within a band about $4^\circ$ of latitude wide, centered at 30$^\circ$N and propagating westwards at the maximal zonal velocity of 35 m s$^{-1}$ of the underlying jet (see [12]), so that in this case $c=u_{\min }<0$. On the other hand, the eastward jovian jet at 7.5$^\circ$S presents chevron-shaped dark spots, consistent with a wave propagating eastwards at a speed equal to the maximum zonal jet velocity $u_{\max }$ of about 140 m s$^{-1}$ (see [46]).
- Saturn’s ribbon at 42$^\circ$N was a type (i4) wave observed in the period 2011–2017 by the Cassini spacecraft mission. In December 2010 a violent storm erupted on Saturn, visible at cloud level between 32$^\circ$N and 38$^\circ$N, lasted for six months and led to a wavy perturbation of the westward jet at 42$^\circ$N, propagating at a speed slightly less than the minimal zonal jet velocity of about 54 m s$^{-1}$ (see [19]).
- The wave dynamics of the zonal band on Jupiter between the westward jet at 19.5$^\circ$S and the eastward jet at 26.5$^\circ$S is quite intricate, comprising the GRS, a filamentary oscillation near its southern boundary and a more intricate wave motion near its northern boundary (see Fig. 3). Within the *f*-plane approximation and with the origin of the coordinate system chosen at the center of the GRS, the nondimensional velocity field within the GRS is of the stationary form
  $$\begin{aligned} u(x,y)=- y\,\mu (\sqrt{x^2+y^2}) \quad \text {and}\quad v(x,y)=x\,\mu (\sqrt{x^2+y^2})\,, \end{aligned}$$
  where $\mu (s)=a -\sqrt{a^2 -b^2 s^k}$ with a>b>0 and k>2 (for the exact values we refer to [8]). Since u=Δu=0 at the core of the GRS because $\mu '(0)=\mu ''(0)=0$, this wave motion is of type (ii).

Note that, according to the recent survey of observed wave-like phenomena on Jupiter [39], the vast majority of waves are found at latitudes with strong prevailing eastward winds, heading in the same direction as Jupiter’s rotation. On Saturn the prevalence of waves in regions associated with prograde motions of the mean zonal flow is also noticeable.

**Fig. 4:**
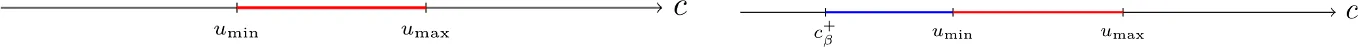
For a genuine traveling wave $(u(x-ct,y),v(x-ct,y))\in C^2({\mathfrak {D}}_L)$, for β=0 the wave speed *c* must belong to *Ran*(*u*), depicted by the red interval, while for β>0 the wave speed *c* either belongs to *Ran*(*u*) or to the interval $[c^{+}_{\beta },u_{\min })$, depicted in blue

Upper and lower bounds for the wave speeds of genuine traveling waves are established in Theorems 3.2 and 3.11 of [10] for β>0 and in [20, 31] for β=0. We summarize these results in the caption of Fig. 4.

In this paper, we provide, by means of Theorem 1.1, a complete classification of genuine traveling waves within the framework of the β-plane approximation. Explicit mathematical examples of traveling waves for each class described in Theorem 1.1 are given in Examples 3.7-3.9, in addition to the observations described above and illustrated in Figs. 1-3. To prove Theorem 1.1, we first analyze in detail which values of *c*, located within the range of the zonal velocity, can arise as wave speeds of genuine traveling waves (see Theorem 3.1). By combining this analysis with the case when *c* lies outside *Ran*(*u*), we then establish the full classification of wave speeds, thereby proving Theorem 1.1. For a genuine traveling wave (u(x-ct,y),v(x-ct,y)), the values of c∈Ran(u) are of the following three types: a generalized inflection value of *u*, or a critical value of *u*, or a maximum/minimum of *u* if β>0, while for β=0 the values of c∈Ran(u) can only be generalized inflection values of *u*. The proof for β>0 proceeds by contradiction and consists of two main parts. In the first part, we use the *F*-formulation (see Lemma 3.4) introduced in [10] and the sign of β to demonstrate that v=0 on the side $\{u<c\}$ of the level set $\{u=c\}$. This also enables us to show that the level set $\{u=c\}$ consists of several straight segments parallel to the zonal direction. In the second part, we show that v=0 on the side $\{u>c\}$ of the level set $\{u=c\}$. We pursue this as a unique continuation problem for the 2-dimensional elliptic equation $\Delta v+{\beta -\Delta u\over u-c}v=0$. A natural approach is to reduce it to a 1-dimensional setting and to analyze it using classical ODE techniques, such as the Grönwall inequality or Fuchs-type methods. One attempt is to fix an arbitrary $x_0$ and to analyze the resulting ODE, but this generates an inhomogeneous term $v_{xx}(x_0,\cdot )$ that is difficult to control. Alternatively, one can exploit the periodicity in *x* and examine the equation mode by mode in Fourier space. In this framework, the nonlinear potential term induces intricate interactions among different modes, yielding a strongly coupled ODE system that is challenging to handle. Consequently, we abandon the 1-dimensional reduction and instead adopt a 2-dimensional approach. We employ the Carleman estimate (3.3)-(3.4) adapted to a boundary-aligned ball (see Fig. 6) around the level set $\{u=c\}$ to handle the Laplacian term. This localized estimate provides the necessary weighted $L^2$ bounds for *w* and ∇w, allowing the lower-order terms to be absorbed by the Laplacian term and yielding a quantitative unique continuation inequality in the ball, where $w\in C^2$ vanishes both near the center and outside the ball. A primary obstacle arises from the singular potential term ${\beta -\Delta u\over u-c}$, which behaves like ${1\over y-y_{i_0}}\notin L^{>1}$ near a branch $\{y=y_{i_0}\}$ of the level set $\{u=c\}$, so that the unique continuation results available in the research literature [3, 29, 44] cannot be invoked in this context. The key observation is that the vanishing of the meridional velocity *v* on the level set $\{u=c\}$ effectively weakens this singularity, enabling the application of the Hardy inequality in the normal direction. This in turn allows the weighted $L^2$ contribution of the singular potential to be controlled by that of the lower-order terms. Combined with the Carleman estimate, this provides us with the mechanism that ultimately establishes the quantitative unique continuation result across $\{y=y_{i_0}\}$. We point out that our method establishes a 2-dimensional unique continuation mechanism for elliptic equations with a borderline ${1\over y-y_{i_0}}$-type potential, when the solution vanishes on the singular set.

Note that Fig. 4 may alternatively be viewed as illustrating a rigidity phenomenon: namely, for a traveling wave $$(u(x-ct,y),v(x-ct,y))\in C^2({\mathfrak D}_L)$$, if the wave speed *c* is lying in the black intervals, then it must be a shear flow. This gives rise to the natural question whether there are sufficient conditions to ensure that a traveling wave with wave speed in the red and blue intervals of Fig. 4 must be a shear flow. This question is challenging due to the singularity in the term $\frac{1}{u-c}$ when the wave speed *c* lies inside the range of zonal velocity *u*. We present some sufficient conditions to ensure that: (i) a traveling wave with wave speed in the red intervals of Fig. 4 (i.e. in the range of the zonal velocity) must be a shear flow (see Theorem 3.10); (ii) a traveling wave with wave speed in the blue and red intervals of Fig. 4 (i.e. with arbitrary wave speed) must be a shear flow (see Theorem 3.12). Note that no restrictions are imposed on the amplitudes of the traveling waves. The sufficient condition in Theorem 3.12 is a narrow range of β, and the proof makes full use of the *F*-formulation introduced in [10] combined with structural properties of the quasi-geostrophic equation. In particular, the argument establishing v=0 on the set $$\{u > c\}$$ differs from that in Theorem 3.1, where the analysis is simplified by exploiting the sign of $$\beta - \Delta u$$. We also extend some of the classification and rigidity results for traveling waves to an unbounded channel in Theorems 4.1, 4.3 and 4.5.

We also investigate whether the rigidity of traveling waves (that is, the absence of genuine traveling waves) holds, or if genuine traveling waves exist near some classes of shear flows. The clarification of this issue plays an important role in the study of long time dynamics near a shear flow [13, 33, 35]. On the one hand, the rigidity of nearby traveling waves is a necessary condition for nonlinear inviscid damping of the shear flow and thus is a first step towards the asymptotic stability. On the other hand, if genuine nearby traveling waves do exist, then the long time dynamics near the shear flow is richer, as these waves might be ‘end states’ of nearby evolutionary flows.

We study the rigidity issue for monotone shear flows in Sect. 5. In an unbounded channel, as an application of Theorem 4.3, we give a simple description of the wave speeds of traveling waves near a monotone shear flow, from which, under the Rayleigh stability condition, we infer the absence of genuine nearby traveling waves (see Corollary 5.1 and Remark 5.2). In a bounded channel, unlike the unbounded case, the situation is more complicated. Under the Rayleigh stability condition, we fully characterize the parameter regimes for (β,L) that lead to the rigidity of traveling waves for arbitrary wave speeds versus those that admit genuine unidirectional traveling waves which are $C^2$-close to the monotone shear flow. When the Rayleigh stability condition does not hold, we determine a parameter regime for (β,L) that admits genuine unidirectional nearby traveling waves. Outside this regime, the wave speed of any genuine nearby traveling wave must be a generalized inflection value (see Theorem 5.10). To prove this for a wave speed locating outside the blue interval in Fig. 4, we can apply Theorems 3.3 and 3.10. However, in general, it is hard to determine whether the rigidity of traveling waves with wave speeds in the blue interval holds or not. Let us point out that our approach for the rigidity part in Theorem 5.10 differs from that in [50], where the method depended heavily on the uniform $H^4$-bound of the $L^2$ normalized meridional velocity, which requires higher regularity. We avoid studying the limit of a sequence of traveling waves, paying instead attention to the intrinsic structure of the nonlinear equation for a specific traveling wave and taking into account the spectral properties of the linearized operator associated with the β-plane equation around the monotone shear flow. Basically, the spectral properties provide us a control from below for the linear part by a uniform $H^1$ bound of the meridional velocity, while the nonlinear part turns out to provide a control from above by a smaller $H^1$ bound, which is an impossible set-up. For the existence part in Theorem 5.10, we can not directly apply the bifurcation lemma established in Lemma 2.5 of [33], since the shear-flow profile lacks sufficient regularity. Our approach is to perturb it to a nearby shear flow with higher regularity. An issue that arises is that it is not clear whether the spectral condition in [33] is satisfied for the perturbed shear flow. To address this, we establish in Lemma 5.6 the continuous dependence of the principal eigenvalue of the singular Rayleigh-Kuo boundary value problem on the shear-flow profile in $C^2$.

In contrast to the unbounded channel, where rigidity (for arbitrary *c*) holds for all (β,L) under the Rayleigh stability condition, the bounded channel admits nearby genuine traveling waves for (β,L) lying in a critical parameter regime. For (β,L) in this critical parameter regime, we further prove that the monotone shear flow is nonlinearly Lyapunov stable. Nevertheless, the nearby coherent traveling waves act as nontrivial asymptotic states so that the shear flow is asymptotically unstable (see Theorem 5.19). This can be viewed as a subtle dynamical phenomenon arising from the inclusion of the Coriolis force, which is a hallmark of geophysical effects. The mechanism leading to asymptotic instability is the appearance of genuine traveling waves with wave speeds outside the zonal velocity, a situation that can never occur when the Coriolis effect is neglected.

Furthermore, by applying Theorem 3.12, we obtain a rigidity result for traveling waves with arbitrary wave speeds near a class of shear flows in Proposition 6.1, and then apply this result to the Couette–Poiseuille flow and the Bickley jet for a certain β-range (see Corollaries 6.3 and 6.5). In the *f*-plane setting, as an application of the classification theorem, we also prove the rigidity of traveling waves with c≠0 near a Kolmogorov flow, and, in contrast, we construct nearby non-sheared steady flows in Proposition 6.11.

The results of this paper complement and improve some recent findings, specific details being provided in remarks throughout the paper. For the 2-dimensional incompressible Euler equation in a bounded periodic channel, the rigidity of steady flows with no horizontal stagnation points is proved in [31]. The sufficient condition to ensure the rigidity of steady flows is weakened to flows with no stagnation points in [20] and with the laminar property in [15]. On the other hand, near the Couette flow, the rigidity of traveling waves with arbitrary speeds is proved in (velocity) $H^{5\over 2}$ [35]. Near the Poiseuille flow, the rigidity of traveling waves with arbitrary speeds is proved in $H^{>6}$ [13], and the rigidity of steady flows is proved in $C^2$ [15]. See also [11, 15, 16, 18, 21, 22, 49] for other rigidity results.

The rest of the paper is organized as follows. In Sect. 2 we recall the governing equations in vorticity form. Section 3 contains the core classification results: we first analyze which values of *c* lying in *Ran*(*u*) can serve as wave speeds of genuine traveling waves (Theorem 3.1), and then combine this with the case c∉Ran(u) to obtain the full classification of wave speeds (Theorem 1.1). We also discuss sufficient conditions for the rigidity of traveling waves with arbitrary amplitudes. Section 4 extends some of the classification and rigidity results to unbounded channels. In Sect. 5 we fully characterize the parameter regimes for (β,L) that yield the following dynamics near a monotone shear flow: (i) the absence of genuine traveling waves; (ii) the existence of genuine traveling waves; and (iii) the constraint that wave speed of any possible genuine traveling wave must be a generalized inflection value. Section 6 is devoted to some further applications to specific non-monotone profiles.

## The Governing Equations

It is convenient to write the β-plane equation (1.3) in the form

2.1 $$\begin{aligned} \partial _{t}\vec {u}+(\vec {u}\cdot \nabla )\vec {u}=-\nabla P-\beta yJ\vec {u}\,,\qquad \nabla \cdot \vec {u}=0 \,, \end{aligned}$$

where $\beta \ge 0$ is the Coriolis parameter, $\vec {u}=(u,v)$ is the fluid velocity, *P* is the modified pressure, and $$ J= \begin{pmatrix} 0 &  -1\\ 1 &  0 \end{pmatrix} $$ is the rotation matrix. We study the fluid flow described by (2.1) in the domain

$$\begin{aligned} D_L=\{(x,y): x\in \mathbb {T}_L,\,y\in [-d,d]\}, \end{aligned}$$

with the non-permeable boundary condition

2.2 $$\begin{aligned} v=0\quad \text {on}\quad y=\pm d. \end{aligned}$$

The non-dimensional boundary-value problem (2.1)-(2.2) in $D_L$ with $d=\frac{d_+ - d_-}{2}$ is equivalent to the system (1.3)-(1.4), as one can see by performing the translation $(x,y) \mapsto (x,y-y_0)$ with $y_0=\frac{d_++d_-}{2}$ the meridional distance from the Equator to the center of the zonal band ${\mathfrak {D}}_L$ and by adjusting the fluid pressure ${\mathfrak {P}}$ to the modified pressure $P={\mathfrak {P}} - (f_0+\beta y_0)\psi$, taking advantage of the existence of a stream function ψ with

$$\begin{aligned} (u,v)=(-\partial _y\psi ,\partial _x\psi ), \end{aligned}$$

ensured by the incompressibility condition $\nabla \cdot \vec {u}=0$.

The vorticity form of (2.1) is the 2-dimensional quasi-geostrophic vorticity equation

2.3 $$\begin{aligned} \partial _{t}\gamma +(\vec {u}\cdot \nabla )\gamma +\beta v=\partial _{t}\gamma +\{\psi ,\gamma \}+\beta v =0, \end{aligned}$$

where $\{\psi ,\gamma \}=\partial _x\psi \partial _y\gamma -\partial _y\psi \partial _x\gamma$ is the Poisson bracket, and $\gamma =\partial _{x}v-\partial _{y}u=\Delta \psi$ is the vorticity of the flow. Alternatively, we can write (2.3) in the form

2.4 $$\begin{aligned} \partial _{t}\Gamma +(\vec {u}\cdot \nabla )\Gamma =0\,, \end{aligned}$$

where $\Gamma =\gamma + \beta y$ is the total vorticity. Note that (2.4) expresses the conservation of total vorticity along a fluid trajectory.

## Classification and Rigidity of Traveling Waves

In this section, we present a comprehensive classification of the genuine traveling-wave solutions of the β-plane equation. Subsequently, without imposing any smallness constraint on the amplitude, we derive some sufficient conditions ensuring the rigidity of traveling waves with wave speeds lying in the red and blue intervals depicted in Fig. 4.

### Classification of Genuine Traveling Waves

We first investigate which values within the range of the zonal velocity are admissible wave speeds for genuine traveling waves.

#### Theorem 3.1

Let $(u(x-ct,y),v(x-ct,y))\in C^2(D_L)$ be a traveling-wave solution to the β-plane equation (2.1)-(2.2). Assume that $\beta -\Delta u\ne 0$ whenever u=c, and one of the conditions

(i) β>0, $$c\in (u_{\min }, u_{\max })$$ and $$\nabla u\ne 0$$ whenever u=c,
(ii) β=0 and c∈Ran(u)
  holds. Then (u(x-ct,y),v(x-ct,y)) is a shear flow.

For β=0, Theorem 1.1 in [20] implies that there exist no genuine traveling waves $(u(x-ct,y),v(x-ct,y))\in C^2(D_L)$ for which *c* lies outside *Ran*(*u*). However, the situation is different when β>0, as illustrated in Example 3.8. In the lemma below, we characterize which values outside *Ran*(*u*) can arise as the wave speed of a genuine traveling wave.

#### Lemma 3.2

Let β>0 and $(u(x-ct,y),v(x-ct,y))\in C^2(D_L)$ be a genuine traveling-wave solution to the β-plane equation (2.1), subject to (4.1). If c∉Ran(u), then $a=c\in [c_\beta ^+,u_{\min })$ satisfies $\Lambda _{n_0}(a)=\beta$ for some integer $n_0\ge 1$, where $c_\beta ^+$ and $\Lambda _{n_0}(a)$ are defined in (1.5)-(1.6) with $d_{\pm }=\pm d$.

Theorem 3.1, together with Theorem 1.1 in [20] and Lemma 3.2, provides a complete classification of the genuine traveling-wave solutions.

#### Theorem 3.3

(=Theorem 1.1) Let $(u(x-ct,y),v(x-ct,y))\in C^2(D_L)$ be a genuine traveling-wave solution to the β-plane equation (2.1)-(2.2).

(i) If β>0, then the wave speed *c* must fall into one of the following four categories:
  *c* is a generalized inflection value of *u*, i.e. $\{\beta -\Delta u=0\}\cap \{u=c\}\ne \emptyset$,
  *c* is a critical value of *u*,
  *c* is a maximum or minimum of *u*,
  $c\in [c_\beta ^+,u_{\min })$ and there exists an integer $n_0\ge 1$ such that $\Lambda _{n_0}(a)=\beta$ holds for a=c, where $c_\beta ^+$ and $\Lambda _{n_0}(a)$ are defined in (1.5)-(1.6) with $d_{\pm }=\pm d$.
(ii) If β=0, then the wave speed *c* must be a generalized inflection value of *u*.

To prove Theorem 3.1 and Theorem 3.3, we employ the *F*-formulation of the β-plane equation introduced in [10].

#### Lemma 3.4

Let $(u(x-ct,y),v(x-ct,y))\in C^2(D_L)$ be a traveling-wave solution to the β-plane equation (2.1)-(2.2). Then the function *F* defined by

3.1 $$\begin{aligned} F={v\over u-c} \end{aligned}$$

solves

3.2 $$\begin{aligned} \partial _{x}((u-c)^2\partial _{x}F)+\partial _{y}((u-c)^2\partial _{y}F)=-\beta (u-c)F \end{aligned}$$

on $$\{u\ne c\}$$.

We will also use the following localized Carleman estimates in the unique-continuation argument for the proof of Theorem 3.1:

#### Lemma 3.5

(Carleman estimates) For any b>0 and any $w\in C^2(\mathbb {T}_L\times [-d,d])$ vanishing outside $B_{\varepsilon _0}$ and on $B_{\varepsilon _0\over 2}$, we have

3.3 $$\begin{aligned} \int _{B_{\varepsilon _0}}r^{-2b-2}e^{2r^{-b}}|w|^2dxdy\le&{1\over 2b^4}\int _{B_{\varepsilon _0}}r^{b+2}e^{2r^{-b}}|\Delta w|^2dxdy, \end{aligned}$$

and

3.4 $$\begin{aligned} \int _{B_{\varepsilon _0}}e^{2r^{-b}}|\nabla w|^2dxdy\le&\left( {1\over 4b^4}+{1\over 2}\varepsilon _0^b+{2\over b^2}\right) \int _{B_{\varepsilon _0}}r^{b+2}e^{2r^{-b}}|\Delta w|^2dxdy, \end{aligned}$$

where $$B_{\varepsilon _0}=B_{\varepsilon _0}(0,0),0<\varepsilon _0< \min \{1,\frac{L}{2}, d\}$$ and $$r=\sqrt{x^2+y^2}$$.

The above Carleman estimates (3.3)-(3.4) provide weighted $L^2$ control of *w* and ∇w in terms of the weighted $L^2$ norm of Δw, and the coefficients on the right-hand side of (3.3)-(3.4) can be made arbitrarily small by choosing b>0 sufficiently large. Moreover, the Carleman weight satisfies $e^{r^{-b}}\rightarrow \infty$ as r→0, and thus enforces a strong amplification of any nonvanishing solution for b>0 sufficiently large. These ingredients are essential for handling the Laplacian contribution in the quantitative unique continuation argument.

The condition that *w* vanishes on $B_{\varepsilon _0\over 2}$ guarantees the existence of the integrals in (3.3)-(3.4), while the condition that *w* vanishes outside $B_{\varepsilon _0}$ ensures that the boundary terms in the integration by parts below vanish. The Carleman estimates (3.3)-(3.4) were derived in [40], a slight difference here being that *b* can be chosen as an arbitrary positive number (while in [40], *b* is required to be sufficiently large). For completeness we provide the proof.

#### Proof

Let

$$\begin{aligned} \eta =e^{r^{-b}}w \end{aligned}$$

on $\mathbb {T}_L\times [-d,d]$. Then η vanishes outside $B_{\varepsilon _0}$ and on $B_{\varepsilon _0\over 2}$. Since

$$\begin{aligned}&r^{b+2}e^{r^{-b}}\left( \nabla e^{-r^{-b}}\cdot \nabla \eta \right) ={b}(x\partial _x\eta +y\partial _y\eta )\quad \text {and}\\&r^{b+2}e^{r^{-b}}\Delta e^{-r^{-b}}={b^2}(r^{-b}-1), \end{aligned}$$

we have

$$\begin{aligned} r^{b+2}e^{2r^{-b}}|\Delta w|^2=&\, r^{b+2}\left( \Delta \eta +2e^{r^{-b}}\left( \nabla e^{-r^{-b}}\cdot \nabla \eta \right) +\eta e^{r^{-b}}\Delta e^{-r^{-b}}\right) ^2\\ \ge&4r^{b+2}e^{r^{-b}}\left( \nabla e^{-r^{-b}}\cdot \nabla \eta \right) \left( \Delta \eta +\eta e^{r^{-b}}\Delta e^{-r^{-b}}\right) \\ =&\, 4{b}(x\partial _x\eta +y\partial _y\eta )(\Delta \eta +{b^2}r^{-b-2}(r^{-b}-1)\eta ). \end{aligned}$$

Then

3.5 $$\begin{aligned} \int _{B_{\varepsilon _0}}r^{b+2}e^{2r^{-b}}|\Delta w|^2dxdy\ge&4b\int _{B_{\varepsilon _0}}\Delta \eta (x\partial _x\eta +y\partial _y\eta )dxdy\nonumber \\&+4b^3\int _{B_{\varepsilon _0}}r^{-b-2}(r^{-b}-1)(x\partial _x\eta +y\partial _y\eta )\eta dxdy. \end{aligned}$$

For the first term in the right-hand side of (3.5) we have

$$\begin{aligned} \int _{B_{\varepsilon _0}}\Delta \eta (x\partial _x\eta +y\partial _y\eta )dxdy =&\, {1\over 2}\int _{B_{\varepsilon _0}}\left( x((\eta _x)^2)_x-x((\eta _y)^2)_x-y((\eta _x)^2)_y+y((\eta _y)^2)_y\right) dxdy\\ =&\, {1\over 2}\int _{B_{\varepsilon _0}}\left( -(\eta _x)^2+(\eta _y)^2+(\eta _x)^2-(\eta _y)^2\right) dxdy=0. \end{aligned}$$

Thus (3.5) becomes

3.6 $$\begin{aligned} \int _{B_{\varepsilon _0}}r^{b+2}e^{2r^{-b}}|\Delta w|^2dxdy\ge&4b^3\int _{B_{\varepsilon _0}}r^{-2b-2}(x\partial _x\eta +y\partial _y\eta )\eta dxdy\nonumber \\&-4b^3\int _{B_{\varepsilon _0}}r^{-b-2}(x\partial _x\eta +y\partial _y\eta )\eta dxdy=I+II. \end{aligned}$$

Since

$$\begin{aligned} \partial _x\left( {x\over r^{2b+2}}\right) +\partial _y\left( {y\over r^{2b+2}}\right) =&\, {r^2-(2b+2)x^2\over r^{2b+4}}+{r^2-(2b+2)y^2\over r^{2b+4}} ={-2b\over r^{2b+2}}, \end{aligned}$$

we have

$$\begin{aligned} I=&\, 2b^3\int _{B_{\varepsilon _0}}r^{-2b-2}(x(\eta ^2)_x+y(\eta ^2)_y) dxdy\\ =&\, -2b^3\int _{B_{\varepsilon _0}}\left( \partial _x\left( {x\over r^{2b+2}}\right) +\partial _y\left( {y\over r^{2b+2}}\right) \right) |\eta |^2 dxdy\\ =&\, 4b^4\int _{B_{\varepsilon _0}}{1\over r^{2b+2}}|\eta |^2 dxdy\ge 0. \end{aligned}$$

Similarly,

$$\begin{aligned} \partial _x\left( {x\over r^{b+2}}\right) +\partial _y\left( {y\over r^{b+2}}\right) =&\, {r^2-(b+2)x^2\over r^{b+4}}+{r^2-(b+2)y^2\over r^{b+4}} ={-b\over r^{b+2}} \end{aligned}$$

implies

$$\begin{aligned} II=&\, -2b^3\int _{B_{\varepsilon _0}}r^{-b-2}(x(\eta ^2)_x+y(\eta ^2)_y)dxdy\\ =&\, 2b^3\int _{B_{\varepsilon _0}}\left( \partial _x\left( {x\over r^{b+2}}\right) +\partial _y\left( {y\over r^{b+2}}\right) \right) |\eta |^2dxdy\\ =&\, -2b^4\int _{B_{\varepsilon _0}}{1\over r^{b+2}}|\eta |^2dxdy\\ \ge&-2b^4\int _{B_{\varepsilon _0}}{1\over r^{2b+2}}|\eta |^2dxdy=-{1\over 2} I. \end{aligned}$$

This, along with (3.6), implies

$$\begin{aligned} \int _{B_{\varepsilon _0}}r^{b+2}e^{2r^{-b}}|\Delta w|^2dxdy\ge&{1\over 2}I+\left( {1\over 2} I+II\right) \ge {1\over 2}I\\ =&\, 2b^4\int _{B_{\varepsilon _0}}{1\over r^{2b+2}}|\eta |^2 dxdy\\ =&\, 2b^4\int _{B_{\varepsilon _0}}{1\over r^{2b+2}}e^{2r^{-b}}|w|^2 dxdy, \end{aligned}$$

which proves (3.3).

We show now the validity of (3.4). Since

$$\begin{aligned} \left| \Delta \left( e^{2r^{-b}}\right) \right| =e^{2r^{-b}}{4b^2\over r^{2b+2}}|1+{1\over 2}r^b|\le e^{2r^{-b}}{8b^2\over r^{2b+2}}, \end{aligned}$$

we infer from integration by parts and (3.3) that

$$\begin{aligned} \int _{B_{\varepsilon _0}}e^{2r^{-b}}|\nabla w|^2dxdy=&\, -\int _{B_{\varepsilon _0}}e^{2r^{-b}} w\Delta wdxdy+{1\over 2}\int _{B_{\varepsilon _0}}\Delta \left( e^{2r^{-b}}\right) | w|^2dxdy\\ \le&\int _{B_{\varepsilon _0}}e^{2r^{-b}} |w||\Delta w|dxdy+{1\over 2}\int _{B_{\varepsilon _0}}\left| \Delta \left( e^{2r^{-b}}\right) \right| | w|^2dxdy\\ \le&\int _{B_{\varepsilon _0}} \left( e^{r^{-b}}r^{-b-1}|w|\right) \left( e^{r^{-b}}r^{b+1}|\Delta w|\right) dxdy\\  &+\int _{B_{\varepsilon _0}}e^{2r^{-b}}{4b^2\over r^{2b+2}}| w|^2dxdy\\ \le&{1\over 2}\int _{B_{\varepsilon _0}} e^{2r^{-b}}r^{-2b-2}|w|^2dxdy\\&+{1\over 2}\int _{B_{\varepsilon _0}} e^{2r^{-b}}r^{2b+2}|\Delta w|^2dxdy\\&+{2\over b^2}\int _{B_{\varepsilon _0}}r^{b+2}e^{2r^{-b}}| \Delta w|^2dxdy\\ \le&{1\over 4b^4}\int _{B_{\varepsilon _0}}r^{b+2}e^{2r^{-b}}| \Delta w|^2dxdy\\&+{1\over 2}\varepsilon _0^b\int _{B_{\varepsilon _0}} e^{2r^{-b}}r^{b+2}|\Delta w|^2dxdy\\&+{2\over b^2}\int _{B_{\varepsilon _0}}r^{b+2}e^{2r^{-b}}| \Delta w|^2dxdy\\ =&\, \left( {1\over 4b^4}+{1\over 2}\varepsilon _0^b+{2\over b^2}\right) \int _{B_{\varepsilon _0}}r^{b+2}e^{2r^{-b}}| \Delta w|^2dxdy. \end{aligned}$$

This proves (3.4). □

Now, we are in a position to prove Theorem 3.1.

#### Proof of Theorem 3.1

The idea of the proof for β>0 is as follows. On the side $\{u<c\}$ of the level set $\{u=c\}$, we make use of the *F*-formulation of the governing equation in Lemma 3.4 and take advantage of the sign of β to show that v=0. This in turn implies that each connected component of $\{u=c\}$ must be a straight segment parallel to the zonal direction and the number of such segments is finite. On the other side $\{u>c\}$, we employ a boundary-adapted Carleman estimate combined with a Hardy inequality to establish unique continuation for an elliptic equation with a borderline ${1\over y-y_{i}}$-type potential near a branch $\{y=y_{i}\}$ of the level set $\{u=c\}$. This yields continuation of v=0 across $\{u=c\}$ and hence throughout the entire domain. In the *f*-plane setting (β=0), the key feature that β=0 renders equation (3.2), and hence the subsequent analysis, considerably simpler than those in the β-plane setting.

Since (u(x-ct,y),v(x-ct,y)) is a traveling-wave solution to the β-plane equation (2.1)-(2.2), we have

3.7 $$\begin{aligned} {(u-c)}\partial _x\gamma +v(\partial _y\gamma +\beta )=0. \end{aligned}$$

By the incompressibility condition we have $\partial _x\gamma =\Delta v$ and $\partial _y\gamma =-\Delta u$, and thus (3.7) can be written as

3.8 $$\begin{aligned} {(u-c)}\Delta v+v(-\Delta u+\beta )=0. \end{aligned}$$

We divide $D_L$ into three parts:

3.9 $$\begin{aligned} S^+=\{(x,y):u>c\}, \quad S^0=\{(x,y):u=c\}, \quad S^-=\{(x,y):u<c\}. \end{aligned}$$

Since $\beta -\Delta u=\beta +\partial _y\gamma \ne 0$ whenever u=c, we infer from (3.7) that

3.10 $$\begin{aligned} v=0 \quad \text {on }\quad S^0\cup \{y=\pm d\}. \end{aligned}$$

Define *F* by (3.1) on $S^{\pm }$. By Lemma 3.4, *F* solves the formulation (3.2) of the β-plane equation on $S^{\pm }$. Any $(x_0,y_0)\in S^0$ is a boundary point of $S^{\pm }$, and thus, since $\beta -(\Delta u)(x_0,y_0)\ne 0$, by the continuity of Δu there exists a neighborhood $U_{(x_0,y_0)}$ of $(x_0,y_0)$ such that $\beta -\Delta u\ne 0$ on $U_{(x_0,y_0)}$. Then by (3.8), (3.10) and $(u,v)\in C^2(D_L)$ we have

3.11 $$\begin{aligned}&\lim _{U_{(x_0,y_0)}\cap S^{\pm }\ni (x,y)\rightarrow (x_0,y_0)}((u-c)^2F\partial F)(x,y)\nonumber \\&\quad =\lim _{U_{(x_0,y_0)}\cap S^{\pm }\ni (x,y)\rightarrow (x_0,y_0)}v\left( \partial v-\partial u{\Delta v\over \Delta u-\beta }\right) (x,y)=0, \end{aligned}$$

where $\partial =\partial _x$ or $\partial _y$. Any $(x_0,y_0)\in \{y=\pm d\}\setminus S^0$ is a boundary point of $S^{\pm }$ and thus $u(x_0,y_0)-c\ne 0$ ensures, by the continuity of *u*, the existence of a neighborhood $V_{(x_0,y_0)}$ of $(x_0,y_0)$ such that u-c≠0 on $V_{(x_0,y_0)}$. By $v(x_0,y_0)=0$ and $(u,v)\in C^1(D_L)$, we have

3.12 $$\begin{aligned}&\lim _{V_{(x_0,y_0)}\cap S^{\pm }\ni (x,y)\rightarrow (x_0,y_0)}((u-c)^2F\partial F)(x,y)\nonumber \\&\quad =\lim _{V_{(x_0,y_0)}\cap S^{\pm }\ni (x,y)\rightarrow (x_0,y_0)}\left( v\partial v-{v^2\partial u\over u-c}\right) (x,y)=0. \end{aligned}$$

Let us first prove the statement under the assumption (i). Note that

3.13 $$\begin{aligned} S^0\cap (\mathbb {T}_L\times (-d,d))\ne \emptyset \quad \text {and}\quad S^{\pm }\cap (\mathbb {T}_L\times (-d,d))\ne \emptyset . \end{aligned}$$

In fact, we choose a curve ϱ such that $\varrho \setminus \{(\tilde{x}_i,\tilde{y}_i)\}_{i=1}^2\subset \mathbb {T}_L\times (-d,d)$ and ϱ connects the points $(\tilde{x}_1,\tilde{y}_1)$ and $(\tilde{x}_2, \tilde{y}_2)$ at which *u* attains its minimum and maximum in $D_L$, respectively. Since $c\in (u_{\min },u_{\max })$, there exists $(\tilde{x}_3,\tilde{y}_3)\in \varrho \setminus \{(\tilde{x}_i,\tilde{y}_i)\}_{i=1}^2\subset \mathbb {T}_L\times (-d,d)$ such that $u(\tilde{x}_3,\tilde{y}_3)=c$. Thus $S^0\cap (\mathbb {T}_L\times (-d,d))\ne \emptyset .$ Then $\nabla u(\tilde{x}_3,\tilde{y}_3)\ne 0$. This implies that $\partial _{x}u(\tilde{x}_3,\tilde{y}_3)\ne 0$ or $\partial _{y}u(\tilde{x}_3,\tilde{y}_3)\ne 0$. If $\partial _y u(\tilde{x}_3,\tilde{y}_3)\ne 0$ (resp. $\partial _x u(\tilde{x}_3,\tilde{y}_3)\ne 0$), there exists $\tilde{\delta }>0$ small enough such that one of the line segments $\{(\tilde{x}_3, y):y\in (\tilde{y}_3-\tilde{\delta },\tilde{y}_3)\}$ and $\{(\tilde{x}_3, y):y\in (\tilde{y}_3,\tilde{y}_3+\tilde{\delta })\}$ (resp. $\{(x, \tilde{y}_3):x\in (\tilde{x}_3-\tilde{\delta },\tilde{x}_3)\}$ and $\{(x, \tilde{y}_3):x\in (\tilde{x}_3,\tilde{x}_3+\tilde{\delta })\}$) lies in $S^+$ and the other is in $S^-$. This validates (3.13).

Based on the assumption that *c* is not a critical value of *u* (i.e. $\nabla u\ne 0$ whenever u=c), we claim that

3.14 $$\begin{aligned} \partial _{x}u=0\quad \text {whenever}\quad u=c, \end{aligned}$$

while

3.15 $$\begin{aligned} \partial _{y}u\ne 0\quad \text {whenever}\quad u=c. \end{aligned}$$

In fact, by (3.13), we have $S^{\pm }\cap (\mathbb {T}_L\times (-d,d))\ne \emptyset$. Multiplying (3.2) by *F* and integrating by parts, we infer from β>0 and the definition of $S^-$ that

3.16 $$\begin{aligned} 0\le \int _{S^-}(u-c)^2|\nabla F|^2dxdy=\int _{S^-} \beta (u-c)|F|^2dxdy\le 0. \end{aligned}$$

Here, the boundary of $S^-$ is contained in $S^0\cup \{y=\pm d\}$, and the boundary terms arising from integration by parts in (3.16) vanish due to (3.11)-(3.12). Then F=0 and

3.17 $$\begin{aligned} v=0 \quad \text {on}\quad S^-. \end{aligned}$$

Since $S^{-}$ is relatively open in $D_L$ and v=0 on $S^-$, we have ∇v=0 on $S^{-}$. By the continuity of ∇v, this extends to ∇v=0 on the boundary set $\{u=c\}$ (i.e. on $S^0$). Due to the incompressibility condition we have $\partial _{x}u=-\partial _{y}v=0$ whenever u=c, which proves (3.14). Under the assumption that $\nabla u\ne 0$ whenever u=c, it follows that (3.15) holds.

**Fig. 5:**
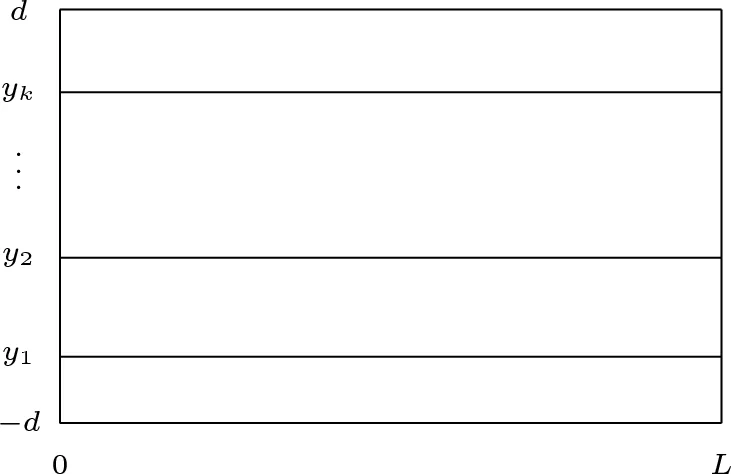
Configuration of a branch of $\{u=c\}$

Using (3.14)-(3.15) and the Implicit Function Theorem, we see that every branch of the level set $\{u=c\}$ is a straight segment parallel to the zonal direction, spanning the full period. Since $\partial _yu$ is continuous and nonzero on the compact set $\{u = c\}$, it attains a strictly positive minimum in absolute value. This implies that distinct segments have a uniform separation and only finitely many such segments can fit in the interval (-d,d). We denote $\{u=c\}\cap (\mathbb {T}_L\times (-d,d))=\{x\in \mathbb {T}_L, y=y_i\}_{i=1}^{k}$ (see Fig. 5), where $y_i<y_{i+1}$ for $1\le i\le k-1$.

By (3.17) we have v=0 on $S^-$. We now prove that v=0 on $S^+$. For this, denote $y_0=-d$, let $y_{k+1}=d$ for convenience and fix $1\le i_0\le k$. Since *c* is not a critical value of *u*, by (3.15) we know that u-c takes opposite signs on the two regions $\mathbb {T}_L\times (y_{i_0-1},y_{i_0})$ and $\mathbb {T}_L\times (y_{i_0},y_{i_0+1})$, indicating that one of the above two regions lies in $S^-$. Without loss of generality, we assume that $\mathbb {T}_L\times (y_{i_0-1},y_{i_0}) \subset S^-$. Then

3.18 $$\begin{aligned} v=0\quad \text {on}\quad \mathbb {T}_L\times (y_{i_0-1},y_{i_0}). \end{aligned}$$

Let $\varepsilon _0\in (0,\min \{1,{L\over 2},{1\over 2}(y_{i_0+1}-y_{i_0}),{1\over 2}(y_{i_0}-y_{i_0-1})\})$. Fix any $x_0\in \mathbb {T}_L$. We denote by $B_{\varepsilon _0}(x_0,y_{i_0}-{\varepsilon _0\over 2})$ the open ball centered at $(x_0,y_{i_0}-{\varepsilon _0\over 2})$ with radius $\varepsilon _0$. Then $B_{\varepsilon _0}(x_0,y_{i_0}-{\varepsilon _0\over 2})\subset \mathbb {T}_L\times (y_{i_0-1},y_{i_0+1})$ while $B_{\varepsilon _0\over 2}(x_0,y_{i_0}-{\varepsilon _0\over 2})\subset \mathbb {T}_L\times (y_{i_0-1},y_{i_0})$, which implies

$$\begin{aligned} v=0\quad \text {on}\quad B_{\varepsilon _0\over 2}\left( x_0,y_{i_0}-{\varepsilon _0\over 2}\right) . \end{aligned}$$

Through the approach of unique continuation for the elliptic equation

3.19 $$\begin{aligned} \Delta v+{\beta -\Delta u\over u-c}v=0 \end{aligned}$$

with the singular potential ${\beta -\Delta u\over u-c}$ near $\{y=y_{i_0}\}$, we will show that

3.20 $$\begin{aligned} v=0\quad \text {on}\quad B_{\varepsilon _0}\left( x_0,y_{i_0}-{\varepsilon _0\over 2}\right) . \end{aligned}$$

Note that since $\beta -\Delta u\ne 0$ and $\partial _y u\ne 0$ on $\{y=y_{i_0}\}$, the singularity of the potential near $\{y=y_{i_0}\}$, is ${\beta -\Delta u\over u-c}\approx {1\over y-y_{i_0}}\notin L^{>1}$, so that the unique continuation results in [3, 29, 44] for the 2-dimensional case do not apply – they require a potential in $L^{>1}$.

**Fig. 6:**
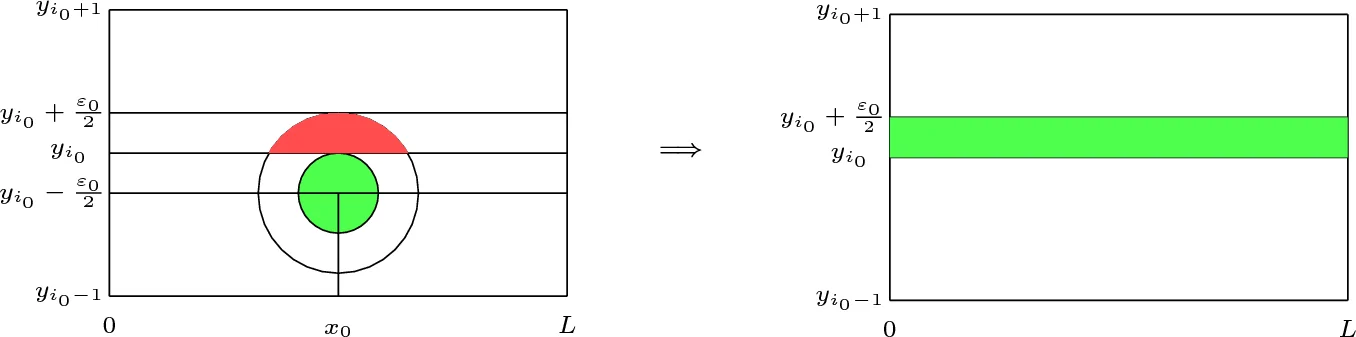
Unique continuation across the level set $\{u=c\}$, propagating vanishing from one side of $\{y=y_{i_0}\}$ to the other side

Note that $\varepsilon _0>0$ is independent of $x_0\in \mathbb {T}_L$. If (3.20) holds true, then by the arbitrary choice of $x_0\in \mathbb {T}_L$, we have v=0 on $\mathbb {T}_L\times (y_{i_0},y_{i_0}+{\varepsilon _0\over 2})$, see Fig. 6. Since ${\beta -\Delta u\over u-c}\in L_{loc}^\infty (\mathbb {T}_L\times (y_{i_0}+{\varepsilon _0},y_{i_0+1}))$, by the classical unique continuation (see, for example, [5, 25, 38]), we have v=0 on $\mathbb {T}_L\times (y_{i_0},y_{i_0+1})$, and thus on $S^+$ by the arbitrary choice of $1\le i_0\le k$.

The rest of the proof under the assumption (i) is devoted to establishing the validity of (3.20). Without loss of generality, we assume that $x_0=0$ and $y_{i_0}-{\varepsilon _0\over 2}=0$. Otherwise, we can perform the change of variables $(x,y)\mapsto (\tilde{x},\tilde{y})=(x-x_0,y-(y_{i_0}-{\varepsilon _0\over 2}))$ and define $\tilde{v}(\tilde{x},\tilde{y})=v(x,y)$. Then the analysis can be carried out for (0, 0) and $\tilde{v}$ instead of $(x_0,y_{i_0}-{\varepsilon _0\over 2})$ and *v*.

Let $a\in \left( {\varepsilon _0\over 2},\varepsilon _0\right)$. Define $\chi \in C^\infty (\mathbb {T}_L\times [-d,d])$ to be a cutoff function such that $\chi \equiv 1$ on $B_a$ and $\chi \equiv 0$ outside $B_{\varepsilon _0}$. Then

$$\begin{aligned} \chi v\in C^2(\mathbb {T}_L\times [-d,d]) \, \text {vanishes on}\, B_{\varepsilon _0\over 2} \, \text {and outside}\, B_{\varepsilon _0}. \end{aligned}$$

Since $\beta -\Delta u\ne 0$ and $\partial _y u\ne 0$ on $\{y=y_{i_0}\}$, by shrinking $\varepsilon _0>0$ if necessary we have

$$\begin{aligned} \left| {\beta -(\Delta u)(x,y)\over u(x,y)-c}\right| =\left| {\beta -(\Delta u)(x,y)\over u(x,y)-u(x,y_{i_0})}\right| \le {C\over |y-y_{i_0}|} \end{aligned}$$

for some C>0, where $(x,y)\in B_{\varepsilon _0}$. By (3.18), we know that $e^{r^{-b}}\chi v=0$ on $\left( \mathbb {T}_L\times (y_{i_0-1},y_{i_0})\right) \cap B_{\varepsilon _0}$. For any $\phi \in H^{1}(a,b)$ that vanishes at some point $y_*\in [a,b]$, the Hardy inequality [24, 33] reads

3.21 $$\begin{aligned} \left\| {\phi \over y-y_*}\right\| _{L^2(a,b)}^2\le C\Vert \phi '\Vert _{L^2(a,b)}^2, \end{aligned}$$

where $a, b\in \mathbb {R}$ with a<b. Then by the Hardy inequality (3.21) in the *y*-direction, we have

3.22 $$\begin{aligned} \left\| {\beta -\Delta u\over u-c}\left( e^{r^{-b}}\chi v\right) \right\| _{L^2(B_{\varepsilon _0})}^2\le&\int _{B_{\varepsilon _0}}{C\over (y-y_{i_0})^2}\left( e^{r^{-b}}\chi v\right) ^2dxdy\nonumber \\ =&\, C\int _{-{\sqrt{3}\over 2}\varepsilon _0}^{{\sqrt{3}\over 2}\varepsilon _0} \int _{\varepsilon _0\over 2}^{\sqrt{\varepsilon _0^2-x^2}}{1\over (y-{\varepsilon _0\over 2})^2}\left( e^{r^{-b}}\chi v\right) ^2dydx\nonumber \\ \le&C\int _{-{\sqrt{3}\over 2}\varepsilon _0}^{{\sqrt{3}\over 2}\varepsilon _0} \int _{\varepsilon _0\over 2}^{\sqrt{\varepsilon _0^2-x^2}}\left| \partial _y\left( e^{r^{-b}}\chi v\right) \right| ^2dydx\nonumber \\ =&\, C\left\| \partial _y\left( e^{r^{-b}}\chi v\right) \right\| _{L^2(B_{\varepsilon _0})}^2, \end{aligned}$$

where we recall that $x_0=0$ and $y_{i_0}-{\varepsilon _0\over 2}=0$. Note that

$$\begin{aligned} \left| \partial _y e^{r^{-b}}\right| ^2=b^2r^{-2b-4}e^{2r^{-b}}y^2\le b^2r^{-2b-2}e^{2r^{-b}}. \end{aligned}$$

By (3.19), (3.22), the Carleman estimate (3.3)-(3.4) and v=χv on $B_a$, we have

3.23 $$\begin{aligned} \int _{B_a}r^{b+2}e^{2r^{-b}}\left| \Delta v\right| ^2dxdy=&\, \int _{B_a}r^{b+2}\left| e^{r^{-b}}{\beta -\Delta u\over u-c} v\right| ^2dxdy\nonumber \\ \le&\int _{B_{\varepsilon _0}}r^{b+2}\left| e^{r^{-b}}{\beta -\Delta u\over u-c}\chi v\right| ^2dxdy\nonumber \\ \le&\left\| {\beta -\Delta u\over u-c}\left( e^{r^{-b}}\chi v\right) \right\| _{L^2(B_{\varepsilon _0})}^2\nonumber \\ \le&C\left\| \partial _y\left( e^{r^{-b}}\chi v\right) \right\| _{L^2(B_{\varepsilon _0})}^2\nonumber \\ \le&C\int _{B_{\varepsilon _0}}\left( e^{2r^{-b}}|\partial _y(\chi v)|^2+\left| \partial _y e^{r^{-b}}\right| ^2|\chi v|^2 \right) dxdy\nonumber \\ \le&C\int _{B_{\varepsilon _0}}\left( e^{2r^{-b}}|\partial _y(\chi v)|^2+b^2r^{-2b-2}e^{2r^{-b}}|\chi v|^2 \right) dxdy\nonumber \\ \le&C \left( {1\over 4b^4}+{1\over 2}\varepsilon _0^b+{2\over b^2}\right) \int _{B_{\varepsilon _0}}r^{b+2}e^{2r^{-b}}|\Delta (\chi v)|^2dxdy\nonumber \\&+{C\over 2b^2}\int _{B_{\varepsilon _0}}r^{b+2}e^{2r^{-b}}|\Delta (\chi v)|^2dxdy\nonumber \\ =&\, C\left( {1\over 4b^4}+{1\over 2}\varepsilon _0^b+{5\over 2b^2}\right) \int _{B_{\varepsilon _0}}r^{b+2}e^{2r^{-b}}|\Delta (\chi v)|^2dxdy. \end{aligned}$$

Choose b>0 large enough such that $C\left( {1\over 4b^4}+{1\over 2}\varepsilon _0^b+{5\over 2b^2}\right) <{1\over 2}$. By (3.23) we have

$$\begin{aligned}&\int _{B_a}r^{b+2}e^{2r^{-b}}\left| \Delta v\right| ^2dxdy \le C\left( {1\over 4b^4}+{1\over 2}\varepsilon _0^b+{5\over 2b^2}\right) \\&\quad \left( \int _{B_{a}}+\int _{B_{\varepsilon _0}\setminus B_a}\right) r^{b+2}e^{2r^{-b}}|\Delta (\chi v)|^2dxdy\\&\quad \le {1\over 2}\int _{B_a}r^{b+2}e^{2r^{-b}}\left| \Delta v\right| ^2dxdy+ C\left( {1\over 4b^4}+{1\over 2}\varepsilon _0^b+{5\over 2b^2}\right) \\&\quad \int _{B_{\varepsilon _0}\setminus B_a}r^{b+2}e^{2r^{-b}}|\Delta (\chi v)|^2dxdy, \end{aligned}$$

which implies

3.24 $$\begin{aligned}&\int _{B_a}r^{b+2}e^{2r^{-b}}\left| \Delta v\right| ^2dxdy\le C\left( {1\over 2b^4}+\varepsilon _0^b+{5\over b^2}\right) \nonumber \\&\quad \int _{B_{\varepsilon _0}\setminus B_a}r^{b+2}e^{2r^{-b}}|\Delta (\chi v)|^2dxdy. \end{aligned}$$

By (3.3) and (3.24), we have

3.25 $$\begin{aligned}&\int _{B_{\varepsilon _0}}r^{-2b-2}e^{2r^{-b}}|\chi v|^2dxdy\nonumber \\&\quad \le {1\over 2b^4}\int _{B_{\varepsilon _0}}r^{b+2}e^{2r^{-b}}|\Delta (\chi v)|^2dxdy\nonumber \\&\quad = {1\over 2b^4}\left( \int _{B_{a}}+\int _{B_{\varepsilon _0}\setminus B_a}\right) r^{b+2}e^{2r^{-b}}|\Delta (\chi v)|^2dxdy\nonumber \\&\quad \le {1\over 2b^4}\left( C\left( {1\over 2b^4}+\varepsilon _0^b+{5\over b^2}\right) +1\right) \int _{B_{\varepsilon _0}\setminus B_a}r^{b+2}e^{2r^{-b}}|\Delta (\chi v)|^2dxdy. \end{aligned}$$

Note that C>0 is independent of b>0. Using (3.25) we obtain

$$\begin{aligned} \int _{B_a}|v|^2dxdy=&\, \int _{B_a}r^{2b+2}r^{-2b-2}|\chi v|^2dxdy\le a^{2b+2}\int _{B_a}r^{-2b-2}|\chi v|^2dxdy\\ =&\, a^{2b+2}\int _{B_a}r^{-2b-2}e^{-2r^{-b}}\left| e^{r^{-b}}\chi v\right| ^2dxdy\\ \le&a^{2b+2}e^{-2a^{-b}}\int _{B_{\varepsilon _0}}r^{-2b-2}e^{2r^{-b}}\left| \chi v\right| ^2dxdy\\ \le&a^{2b+2}e^{-2a^{-b}}{1\over 2b^4}\left( C\left( {1\over 2b^4}+\varepsilon _0^b+{5\over b^2}\right) +1\right) \\&\quad \int _{B_{\varepsilon _0}\setminus B_a}r^{b+2}e^{2r^{-b}}|\Delta (\chi v)|^2dxdy\\ \le&a^{2b+2}{1\over 2b^4}\left( C\left( {1\over 2b^4}+\varepsilon _0^b+{5\over b^2}\right) +1\right) \varepsilon _0^{b+2}\\&\quad \int _{B_{\varepsilon _0}\setminus B_a}|\Delta (\chi v)|^2dxdy\\ \rightarrow&0\quad \text {as}\quad b\rightarrow \infty , \end{aligned}$$

due to $a<\varepsilon _0<1$, $a^{2b+2}{1\over 2b^4}\left( C\left( {1\over 2b^4}+\varepsilon _0^b+{5\over b^2}\right) +1\right) \varepsilon _0^{b+2}\rightarrow 0$ as $b\rightarrow \infty$, and to the fact that the integral $\int _{B_{\varepsilon _0}\setminus B_a}|\Delta (\chi v)|^2dxdy$ is independent of b>0. Thus v=0 on $B_a$. Since $a\in ({\varepsilon _0\over 2},\varepsilon _0)$ is arbitrary we have v=0 on $B_{\varepsilon _0}$. This shows the validity of (3.20) under the assumption (i).

Now we prove the statement under the assumption (ii). Recall the partition of the domain $D_L$ into $S^+, S^0$ and $S^-$ according to (3.9), and define *F* on $S^{\pm }$ as in (3.1). The assumption that $\Delta u\ne 0$ whenever u=c is used at two points. First, evaluating

$$\begin{aligned} {(u-c)}\Delta v-v\Delta u=0 \end{aligned}$$

on $S^0$ and using the assumption $\Delta u\ne 0$ there, we obtain v=0 on $S^0$. Second, let $(x_0,y_0)\in S^0$. Since $(\Delta u)(x_0,y_0)\ne 0$, the continuity of Δu ensures that there exists a neighborhood $U_{(x_0,y_0)}$ of $(x_0,y_0)$ such that $\Delta u\ne 0$ on $U_{(x_0,y_0)}$. Consequently, for $\partial =\partial _x$ or $\partial _y$,

$$\begin{aligned}&\lim _{U_{(x_0,y_0)}\cap S^{\pm }\ni (x,y)\rightarrow (x_0,y_0)}((u-c)^2F\partial F)(x,y)\\&\quad =\lim _{U_{(x_0,y_0)}\cap S^{\pm }\ni (x,y)\rightarrow (x_0,y_0)}v\left( \partial v-\partial u{\Delta v\over \Delta u }\right) (x,y)=0. \end{aligned}$$

For $(x_0,y_0)\in \{y=\pm d\}\setminus S^0$, equation (3.12) remains valid. Thus, the boundary terms on $S^0\cup \{y=\pm d\}$ vanish in the integration by parts used below to derive (3.27).

Since β=0, by Lemma 3.4 the *F*-formulation of the β-plane equation simplifies to

3.26 $$\begin{aligned} \partial _{x}((u-c)^2\partial _{x}F)+\partial _{y}((u-c)^2\partial _{y}F)=0. \end{aligned}$$

Multiplying (3.26) by *F*, integrating over $S^{\pm }$, and using the vanishing of the boundary terms, we obtain

3.27 $$\begin{aligned} \int _{S^{\pm }}(u-c)^2|\nabla F|^2dxdy=0. \end{aligned}$$

Thus, ∇F=0 on $S^{\pm }$ (i.e. ∇F=0 on both sides of the level set $\{u=c\}$). We show now that v=0 on $S^{\pm }$. Since the considerations for $S^{\pm }$ are similar, we only give the proof for $S^{-}$. Let $S_1^{-}$ be a connected component of $S^{-}$. Then ∇F=0 and thus $F={v\over u-c}=a_{1}$ on $S_1^{-}$, where $a_{1}\in \mathbb {R}$ is a constant. If $a_{1}=0$, then $F={v\over u-c}=0$ and v=0 on $S_1^{-}$. If $a_{1}\ne 0$, by the incompressibility condition we have $\partial _xu+\partial _y v={1\over a_{1}}\partial _x v+\partial _y v=0$ on $S_1^{-}$. Let $(x_1,y_1)\in S_1^{-}$ and $\{(x_1+{1\over a_1} s, y_1+s), s\in \mathbb {R}\}$ be a line. Then there exists $s_1>0$ such that $(x_1+{1\over a_1} s_1, y_1+s_1)\in S^0\cup \{y=\pm d\}$ and $\{(x_1+{1\over a_1} s, y_1+s), s\in [0,s_1)\}\subset S_1^-$. Since

$$\begin{aligned} \partial _sv\left( x_1+{1\over a_1} s, y_1+s\right) =\left( {1\over a_1}\partial _xv+\partial _yv\right) \left( x_1+{1\over a_1} s, y_1+s\right) =0 \end{aligned}$$

on $s\in [0,s_1)$, $v\in C^1(D_L)$ and $v(x_1+{1\over a_1} s_1, y_1+s_1)=0$, we have $v(x_1,y_1)=0$. However, $v=a_1(u-c)\ne 0$ on $S_1^-$, which is a contradiction. In conclusion, we have v=0 on $S^-$. □

#### Remark 3.6

We provide a supplement to Theorem 3.1 (*i*). For a traveling-wave solution $(u(x-ct,y),v(x-ct,y))\in C^2(D_L)$ to the β-plane equation (2.1)-(2.2), if $\beta -\Delta u\ne 0$ whenever u=c, β>0 and $c=u_{\max }$, then it is a shear flow.

In fact, in the case β>0 and $c=u_{\max }$, we have $S^+=\emptyset$ and v=0 on $S^0$ due to (3.8) and $\beta -\Delta u\ne 0$ whenever u=c. Moreover, by (3.16) we have v=0 on $S^-$. Here, $S^{\pm }$ and $S^0$ are defined in (3.9).

#### Proof of Lemma 3.2

For the parameter $a\in (-\infty , u_{\min })$, the elliptic boundary value problem

3.28 $$\begin{aligned} -\partial _{x}((u-a)^2\partial _{x}G)-\partial _{y}((u-a)^2\partial _{y}G)=\Lambda (u-a)G,\quad G\in H_0^1(D_L)\cap H^2(D_L) \end{aligned}$$

has infinitely countable eigenvalues $\{\Lambda _n(a)\}_{n=1}^\infty$, which are all real and can be arranged in the non-decreasing order: $\Lambda _n(a)\le \Lambda _{n+1}(a)$ for any integer n≥1, counting repeatedly according to their multiplicity. Moreover, the *n*-th eigenvalue $\Lambda _n(a)$ has the variational expression

$$\begin{aligned} \Lambda _{n}(a)=\sup _{V\subset H_0^1({ D}_L),\dim V=n-1}\,\inf _{G\perp V, G\in H_0^1({ D}_L), G\ne 0}{\int _{{D}_L}(u-a)^2|\nabla G|^2dxdy\over \int _{{ D}_L} (u-a)G^2dxdy}. \end{aligned}$$

For an integer n≥1, $\{\Lambda _n(a), a\in (-\infty , u_{\min })\}$ forms an eigenvalue curve.

Since c∉Ran(u), by Theorems 3.2 and 3.11 of [10] we have $c\in [c_\beta ^+,u_{\min })$. By Lemma 3.4, the function *F* defined by (3.1) solves (3.28) with a=c and $\Lambda =\beta$ on the whole domain $D_L$. This implies that β is an eigenvalue of the elliptic boundary value problem (3.28) with a=c. Then there exists some integer $n_0\ge 1$ such that $\Lambda _{n_0}(a)=\beta$ holds for a=c. □

#### Proof of Theorem 3.3

Let β>0. Since (u(x-ct,y),v(x-ct,y)) is a genuine traveling wave, by Theorems 3.2 and 3.11 in [10] we have $c\in [c^{+}_{\beta },u_{\max }]$. If *c* is outside *Ran*(*u*), then by Lemma 3.2 we know that $c\in [c_\beta ^+,u_{\min })$ solves $\Lambda _{n_0}(c)=\beta$ for some integer $n_0\ge 1$. Next, we consider c∈Ran(u). If $c\in (u_{\min }, u_{\max })$, by Theorem 3.1 (i), we know that *c* is either a generalized inflection value of *u* or a critical value of *u*.

For β=0, since (u(x-ct,y),v(x-ct,y)) is a genuine traveling wave, by Theorem 1.1 in [20] (or Theorem 1.1 in [31]) we know that c∈Ran(u). By Theorem 3.1 (ii), *c* must be a generalized inflection value of *u*. □

We now provide mathematical examples to demonstrate that genuine traveling waves in each of the classification types described in Theorem 1.1 do exist.

#### Example 3.7

In the β-plane setting there are genuine traveling waves whose wave speeds are generalized inflection values of the zonal velocity. As in Example 3.1 from [10], we consider the flow in the channel $\mathbb {T}_{2\pi }\times [-1,1]$ with stream function ψ given by

$$\begin{aligned} \psi (x,y)= &   \, {{A}\over {n}}\cos \left( \frac{(2k+1)\pi y}{2}\right) \sin (nx)\\  &   +\tilde{A}\cos (\sqrt{-\lambda }y)+B\sin (\sqrt{-\lambda }y) +\frac{\lambda c-\beta }{-\lambda }y+\frac{\xi }{-\lambda }, \end{aligned}$$

with integers n,k≥1, $\lambda =-n^{2}-\frac{(2k+1)^2\pi ^2}{4},$ and real constants *c*, ξ, *A*, $\tilde{A}$ and *B*. Since

3.29 $$\begin{aligned} \left\{ \begin{aligned} \Delta \psi +\beta y=\lambda (\psi +cy)+\xi , \\ \partial _{x}\psi =0 \quad \text {on}\quad y=\pm 1, \end{aligned} \right. \end{aligned}$$

the corresponding velocity field (u(x-ct,y),v(x-ct,y)) is a traveling-wave solution to (2.1)-(2.2). It follows from (3.8) and (3.29) that $({\beta -\Delta u})/(u-c)=-\Delta v/v=-\lambda .$ This yields that $\{\beta -\Delta u=0\}=\{u=c\}\ne \emptyset$ as long as c∈Ran(u). Hence, *c* is a generalized inflection value of *u* provided that c∈Ran(u).

#### Example 3.8

In the β-plane setting, there are genuine traveling waves whose wave speeds are of the following types: (i) a minimum of the zonal velocity; (ii) a critical value of the zonal velocity, (iii) they lie outside the range of the zonal velocity. To see this consider, as in Example 3.13 from [10], the genuine traveling-wave flow (u(x-ct,y),v(x-ct,y)) in $\mathbb {T}_{2\pi }\times [-1,1]$ with stream function

$$\begin{aligned} \psi (x,y)=\left( \frac{-\beta }{{\pi ^2\over 4}+1}-c\right) y+\cos (x)\cos \left( \frac{\pi y}{2}\right) . \end{aligned}$$

A direct computation yields

$$\begin{aligned} Ran(u)=\left[ c+\frac{\beta }{{\pi ^2\over 4}+1}-\frac{\pi }{2},c+\frac{\beta }{{\pi ^2\over 4}+1}+\frac{\pi }{2}\right] . \end{aligned}$$

Let $$\beta _0={\pi \over 2}({\pi ^2\over 4}+1)$$.

(i) If $\beta =\beta _0>0$, then $u_{\min }=c+\frac{\beta }{{\pi ^2\over 4}+1}-\frac{\pi }{2}=c$, so that *c* is a minimum of *u*.
(ii) For $\beta =\beta _0$, we have u(π,1)=c and $(\nabla u)(\pi ,1)=0$. Thus, *c* is a critical value of *u*.
(iii) It is shown in Example 3.13 of [10] that for $\beta >\beta _0$, *c* is outside *Ran*(*u*), more specifically, $c\in (c_\beta ^+,u_{\min })$. Since (*u*, *v*) solves (3.8), the function $F={v\over u-c}$ solves (3.28) with a=c and $\Lambda =\beta$, which implies $\Lambda _{n_0}(c)=\beta$ for some $n_0\ge 1$.

#### Example 3.9

In the *f*-plane setting, there are genuine traveling waves whose wave speeds are generalized inflection values of the zonal velocity. Indeed, for any $\varepsilon \in \mathbb {R}$, consider the genuine steady solution with velocity field

3.30 $$\begin{aligned} (u_\varepsilon ,v_\varepsilon )=\left( \sin (y)+{\varepsilon \over 2}\sin \left( {y\over 2}\right) \sin \left( {\sqrt{3}\over 2}x\right) ,{\sqrt{3}\varepsilon \over 2}\cos \left( {y\over 2}\right) \cos \left( {\sqrt{3}\over 2}x\right) \right) \end{aligned}$$

in the channel $\mathbb {T}_{L_0}\times [-\pi ,\pi ]$ with $L_0={4\pi \over \sqrt{3}}$. Since

$$\begin{aligned} -\Delta u_\varepsilon =u_\varepsilon =\sin (y)+{\varepsilon \over 2}\sin \left( {y\over 2}\right) \sin \left( {\sqrt{3}\over 2}x\right) \,, \end{aligned}$$

we see that (*x*, 0) is a generalized inflection point of $u_\varepsilon$ for any $x\in \mathbb {T}_{L_0}$ and $c=0=u_\varepsilon (x,0)$. Thus *c* is a generalized inflection value of $u_\varepsilon$.

### Rigidity of Traveling Waves with Arbitrary Amplitudes

We first establish a rigidity result for traveling waves with wave speeds lying in the red intervals of Fig. 4. The conclusions differ between the *f*-plane and β-plane settings.

#### Theorem 3.10

Let $\beta \notin Ran(\Delta u)$ and $(u(x-ct,y),v(x-ct,y))\in C^2(D_L)$ be a traveling-wave solution to the β-plane equation (2.1)-(2.2). Assume that one of the following conditions

(i) β>0, $$c\notin [c_\beta ^+, u_{\min }]$$ and $$\nabla u\ne 0$$ on $$D_L$$,
(ii) β=0 and $c\in \mathbb {R}$ holds.

Then (u(x-ct,y),v(x-ct,y)) is a shear flow, where $c_\beta ^{+}$ is defined in (1.5) with $d_{\pm }=\pm d$.

#### Proof

Let β>0. By Theorem 3.3 (i), if $c\notin [c_\beta ^+, u_{\min }]\cup \{u_{\max }\}$, then (u(x-ct,y),v(x-ct,y)) is a shear flow. It follows from Remark 3.6 that if $c=u_{\max }$, then (u(x-ct,y),v(x-ct,y)) is a shear flow.

Let β=0. By Theorem 3.3 (ii), (u(x-ct,y),v(x-ct,y)) is a shear flow for all $c\in \mathbb {R}$. □

#### Remark 3.11

Recall that in the β-plane setting $[c_\beta ^+, u_{\min }]$ is the blue interval of Fig. 4. Within this setting, there may exist traveling waves with speeds in the blue interval $[c_\beta ^+, u_{\min }]$, see Example 3.8. In general, it is hard to determine whether the rigidity of traveling waves with speeds in the blue interval holds or not. Nevertheless,

- for certain specific ranges of β, in Theorem 3.12 we prove the rigidity of traveling waves with speeds in the blue interval (and thus for arbitrary wave speeds),
- for traveling waves near monotone shear flows under the Rayleigh stability condition, in Theorem 5.10 we determine for which β the rigidity of traveling waves with speeds in the blue interval holds for all zonal periods *L*, while for other values of β there exist genuine traveling waves with a specific zonal period $L_0$.

Next, we consider the rigidity of traveling waves with wave speeds in the red and blue intervals of Fig. 4.

#### Theorem 3.12

Assume that one of the conditions

(i) $$(\Delta u)_{\min }>0$$ and $$\beta \in (0,(\Delta u)_{\min })$$,
(ii) $\Delta u\ne 0$ on $D_L$ and β=0
  holds. If $(u(x-ct,y),v(x-ct,y))\in C^2(D_L)$ is a traveling-wave solution to the β-plane equation (2.1)-(2.2) with $c\in \mathbb {R}$, then (u(x-ct,y),v(x-ct,y)) is a shear flow.

#### Remark 3.13

In a bounded periodic channel, Theorem 3.12 offers a generalization of Corollary $1.6\, (\textrm{i})$ in [18] through a different approach, improving it in two aspects: one extension is from the *f*-plane to the β-plane setting, and the other is that the amplitudes of the traveling waves are arbitrary, with no smallness constraint. In Subsection 6.1 we present its application to the rigidity of traveling waves near a class of shear flows which include the Couette–Poiseuille flow and the Bickley jet.

#### Proof

Since (u(x-ct,y),v(x-ct,y)) is a traveling-wave solution to the β-plane equation (2.1)-(2.2), we know that (3.7)-(3.8) hold. We partition the domain $D_L$ into $S^+, S^0$ and $S^-$ according to (3.9).

We first prove the statement under the assumption (i). Since $\beta \in (0,(\Delta u)_{\min })$, we have

3.31 $$\begin{aligned} \beta +\partial _y \gamma =\beta -\Delta u\le \beta -(\Delta u)_{\min }<0 \end{aligned}$$

for $(x,y)\in D_L$. This, along with (3.8), implies that

3.32 $$\begin{aligned} v=0 \quad \text {on }\quad \{y=\pm d\}\cup S^0. \end{aligned}$$

We now prove that v=0 on $S^-$. We define *F* on $S^-$ as in (3.1) and then use the *F*-formulation of the β-plane equation (3.2) on $S^-$. Any $(x_0,y_0)\in S^0\cup \{y=\pm d\}$ being a boundary point of $S^-$, by (3.8), (3.31)-(3.32) and $(u,v)\in C^2(D_L)$ we have

3.33 $$\begin{aligned}&\lim _{S^-\ni (x,y)\rightarrow (x_0,y_0)}((u-c)^2F\partial F)(x,y)=\lim _{S^-\ni (x,y)\rightarrow (x_0,y_0)}\left( v\partial v-{v^2\partial u\over u-c}\right) (x,y)\nonumber \\&\quad =\lim _{S^-\ni (x,y)\rightarrow (x_0,y_0)}v\left( \partial v-\partial u{\Delta v\over \Delta u-\beta }\right) (x,y)=0, \end{aligned}$$

where $\partial =\partial _x$ or $\partial _y$. Thus, multiplying (3.2) by *F* and integrating by parts, we infer from the definition of $S^-$ and β>0 that

$$\begin{aligned} 0\le \int _{S^-}(u-c)^2|\nabla F|^2dxdy=\int _{S^-} \beta (u-c)|F|^2dxdy\le 0. \end{aligned}$$

Therefore, F=0 and v=0 on $S^-$.

We now prove that v=0 on $S^+$. Unlike the proof of Theorem 3.1, the present argument takes advantage of the sign of $\partial _y\gamma +\beta$ in (3.31). By (3.8) the original governing equation of the traveling wave (u(x-ct,y),v(x-ct,y)) is

3.34 $$\begin{aligned} \Delta v+\frac{ \partial _y\gamma +\beta }{u-c}v=0\quad \text {on}\quad S^+. \end{aligned}$$

Since $\partial _y \gamma +\beta <0$ and u-c>0 on $S^+$, it follows that $\frac{ \partial _y\gamma +\beta }{u-c}<0$ on $S^+$. Using (3.32) and the fact that $v\in C^2(D_L)$, we can apply the weak maximum principle for elliptic equations (see, for example, Theorem 2.3 in [23]) to both *v* and -v, which immediately yields

$$\begin{aligned} v= 0 \end{aligned}$$

on any connected component of $S^+$.

By Theorem 3.10, (u(x-ct,y),v(x-ct,y)) is a shear flow under the assumption (ii). □

## Results for An Unbounded Channel

We study the fluid flow in the unbounded channel

$$\begin{aligned} D_L^{\infty }=\{(x,y): x\in \mathbb {T}_L,\,y\in (-\infty ,+\infty )\}, \end{aligned}$$

with the asymptotic behavior

4.1 $$\begin{aligned} v\rightarrow 0\quad \text {as}\quad y\rightarrow \pm \infty \end{aligned}$$

for $x\in \mathbb {T}_L$. Denote that

$$\begin{aligned} F_{\sup }=\sup _{(x,y)\in D_L^{\infty }}\{F(x,y)\}\,,\quad F_{\inf }=\inf _{(x,y)\in D_L^{\infty }}\{F(x,y)\}\, \end{aligned}$$

for a function $$F \in C(D_L^{\infty })$$.

We study the propagation speeds and structural properties of traveling waves, extending prior results to an unbounded channel. First, we extend Theorems 3.2 and 3.11 in [10] to an unbounded channel, thus addressing the rigidity issue for wave speeds located outside the range of the zonal velocity.

### Theorem 4.1

Let $(u(x-ct,y),v(x-ct,y))\in C^2(D_L^{\infty })$ be a traveling-wave solution to the β-plane equation (2.1), subject to (4.1). Assume that $v\partial _yv\rightarrow 0$ and $v^2\partial _y u\rightarrow 0$ as $y\rightarrow \pm \infty$ for $x\in \mathbb {T}_L$, and one of the conditions

(i) β>0 and $$c>u_{\sup }$$;
(ii) β=0 and $$c\notin \overline{Ran(u)}$$
  holds. Then (u(x-ct,y),v(x-ct,y)) is a shear flow.

### Remark 4.2

The condition “$v\partial _yv\rightarrow 0$ and $v^2\partial _y u\rightarrow 0$ as $y\rightarrow \pm \infty$ for $x\in \mathbb {T}_L$" is mild. In fact, by (4.1) and the incompressibility condition, $u\in \dot{W}^{1,\infty }(D_L^{\infty })=\{u:\partial _xu,\partial _y u\in L^\infty (D_L^\infty )\}$ is sufficient to guarantee this.

Note that in the unbounded case, we do not obtain a lower bound for the wave speeds of genuine traveling waves in the β-plane setting. This is because the unbounded domain prevents the Poincaré inequality (see (3.29) in [10]) from being carried out. This can also be seen from $c_\beta ^+\rightarrow -\infty$ as $d_{\pm }\rightarrow \pm \infty$, where $c_\beta ^+$ is defined in (1.5).

### Proof

Since *c* does not touch $\overline{Ran(u)}$, we can define $F={v\over u-c}$ on the whole domain $D_{L}^\infty$. By Lemma 3.4, the *F*-formulation of the β-plane equation is

$$\begin{aligned} \partial _{x}((u-c)^2\partial _{x}F)+\partial _{y}((u-c)^2\partial _{y}F)=-\beta (u-c)F\quad \text {on}\quad D_L^\infty . \end{aligned}$$

Since $v\partial _yv\rightarrow 0$ and $v^2\partial _y u\rightarrow 0$ as $y\rightarrow \pm \infty$, we have

4.2 $$\begin{aligned} \lim _{y\rightarrow \pm \infty }((u-c)^2F\partial _y F)(x,y) =\lim _{y\rightarrow \pm \infty }v\left( \partial _y v-{v\partial _y u\over u-c}\right) (x,y)=0 \end{aligned}$$

for $x\in \mathbb {T}_L$. For any d>0, we have

4.3 $$\begin{aligned}&\int _{-d}^d\int _{\mathbb {T}_L}(u-c)^2|\nabla F|^2dxdy-\int _{\mathbb {T}_L}((u-c)^2\partial _yFF)\Big |_{y=-d}^ddx\nonumber \\&\quad =\int _{-d}^d\int _{\mathbb {T}_L} \beta (u-c)|F|^2dxdy. \end{aligned}$$

If one of (i) and (ii) holds, then $\beta (u-c)\le 0$ on $D_L^\infty$. We claim that ∇F=0 on $D_L^\infty$, which implies v=0 on $D_L^\infty$ by (4.1). Otherwise, there exists $d_0>0$ such that

4.4 $$\begin{aligned}&\int _{-d_0}^{d_0}\int _{\mathbb {T}_L}(u-c)^2|\nabla F|^2dxdy>0. \end{aligned}$$

By (4.2) we can choose $d_1>d_0$ such that

$$\begin{aligned}&\int _{\mathbb {T}_L}((u-c)^2\partial _y F F)|_{y=-d_1}^{d_1}dx<{1\over 2}\int _{-d_0}^{d_0}\int _{\mathbb {T}_L}(u-c)^2|\nabla F|^2dxdy. \end{aligned}$$

Now, using (4.3)-(4.4), $\beta (u-c)\le 0$ on $D_L^\infty$ and $d_1>d_0$, we obtain

4.5 $$\begin{aligned} 0\ge&\int _{-d_1}^{d_1}\int _{\mathbb {T}_L} \beta (u-c)|F|^2dxdy\nonumber \\>&\int _{-d_1}^{d_1}\int _{\mathbb {T}_L}(u-c)^2|\nabla F|^2dxdy-{1\over 2}\int _{-d_0}^{d_0}\int _{\mathbb {T}_L}(u-c)^2|\nabla F|^2dxdy\nonumber \\ \ge&{1\over 2}\int _{-d_0}^{d_0}\int _{\mathbb {T}_L}(u-c)^2|\nabla F|^2dxdy>0, \end{aligned}$$

which is a contradiction. □

We now extend the classification of the traveling waves to an unbounded channel. We do not intend to get the generalized version as in Theorem 3.3, but focus on some special case, which is applicable to the traveling waves near monotone shear flows in the next section.

### Theorem 4.3

Let $(u(x-ct,y),v(x-ct,y))\in C^2(D_L^{\infty })$ be a genuine traveling-wave solution to the β-plane equation (2.1), subject to (4.1). Assume that $|\partial _{y}u|\ge C_0$ on $D_L^{\infty }$ for some $C_0>0$, $v\in \dot{W}^{1,\infty }(D_L^{\infty })$, and ${v^2\partial _y u\over u}\rightarrow 0$ as $y\rightarrow \pm \infty$ for $x\in \mathbb {T}_L$. Then the wave speed *c* must be a generalized inflection value of *u*.

### Remark 4.4

$(\textrm{i})$ Note that $\lim _{y\rightarrow \pm \infty }|u(x,y)|=\infty$ and thus Ran(u(x,·))=Ran(u) spans $\mathbb {R}$ for $x\in \mathbb {T}_L$ in Theorem 4.3, a situation that differs from the case of a bounded channel (for which *Ran*(*u*) is compact).

$\mathrm{(ii)}$ By (4.1), a sufficient condition for the asymptotic behavior “${v^2\partial _y u\over u}\rightarrow 0$ as $y\rightarrow \pm \infty$ for $x\in \mathbb {T}_L$" is ${\partial _{y}u\over u}$ is bounded for |*y*| sufficiently large.

### Proof

The proof essentially follows the same approach as in Theorem 3.1. We highlight only a difference, primarily concerning the asymptotic behavior as $y\rightarrow \pm \infty$.

Assume that *c* is not a generalized inflection value of *u*. We will show that (u(x-ct,y),v(x-ct,y)) is a shear flow. Note that $Ran (u)=Ran(u(x,\cdot ))=\mathbb {R}$ for $x\in \mathbb {T}_L$, due to $|\partial _{y}u|\ge C_0$ on $D_L^{\infty }$ for some $C_0>0$. Then there exists a unique $y_x\in \mathbb {R}$ such that $u(x,y_x)=c$ for $x\in \mathbb {T}_L$. This, combined with (3.14), implies that

$$ S^0=\{(x,y_x):x\in \mathbb {T}_L\} $$

is a straight segment parallel to the zonal direction. The subset $S^-$ lies on one side of the curve $S^0$, while $S^+$ occupies the other side. The difference is that the calculation of the boundary term in (3.12) should be replaced by the asymptotic behavior

$$\begin{aligned} \lim _{y\rightarrow \pm \infty }((u-c)^2F\partial _y F)(x,y) =\lim _{y\rightarrow \pm \infty }v\left( \partial _y v-{v\partial _y u\over u-c}\right) (x,y)=0 \end{aligned}$$

for $x\in \mathbb {T}_L$, due to (4.1), $\partial _y v\in L^{\infty }(D_L^\infty )$ and ${v^2\partial _y u\over u}\rightarrow 0$ as $y\rightarrow \pm \infty$. Now v=0 on $S^-$ follows from an argument similar to that in (4.3)-(4.5). □

Finally, we extend Theorem 3.12 to the unbounded channel, providing sufficient conditions for the rigidity of traveling waves with arbitrary wave speeds.

### Theorem 4.5

Let $(u(x-ct,y),v(x-ct,y))\in C^2(D_L^{\infty })$ be a traveling-wave solution to the β-plane equation (2.1), subject to (4.1). Assume that $v\in \dot{W}^{2,\infty }(D_L^{\infty })=\{v:\partial _xv,\partial _y v, \Delta v\in L^\infty (D_L^\infty )\}$, $v\partial _yu\rightarrow 0$ as $y\rightarrow \pm \infty$ for $x\in \mathbb {T}_L$, and one of the conditions

(i) $$(\Delta u)_{\inf }>0$$, $$\beta \in (0,(\Delta u)_{\inf })$$;
(ii) $0=\beta \notin \overline{Ran(\Delta u)}$ holds. Then (u(x-ct,y),v(x-ct,y)) is a shear flow for an arbitrary $c\in \mathbb {R}$.

### Remark 4.6

By (4.1), $\partial _{y}u\in L^{\infty }(D_L^{\infty })$ is sufficient to ensure $v\partial _yu\rightarrow 0$ as $y\rightarrow \pm \infty$ for $x\in \mathbb {T}_L$.

### Proof

Under the assumption (i), we follow the approach used in Theorem 3.12 (i). We only discuss the differences. For $(x_0,y_0)\in S^0$ a boundary point of $S^-$, we still have the calculation of the boundary term (3.33). As $y\rightarrow \pm \infty$, instead of (3.33) for the boundary points in $\{y=\pm d\}$, we need the asymptotic behavior

4.6 $$\begin{aligned} \lim _{y\rightarrow \pm \infty }((u-c)^2F\partial _y F)(x,y) =\lim _{y\rightarrow \pm \infty }v\left( \partial _y v-\partial _y u{\Delta v\over \Delta u-\beta }\right) (x,y)=0 \end{aligned}$$

for $x\in \mathbb {T}_L$, due to (4.1), $\Delta u-\beta \ge (\Delta u)_{\inf }-\beta >0$ for $(x,y)\in D_L^\infty$, $v\in \dot{W}^{2,\infty }(D_L^\infty )$, and $v\partial _yu\rightarrow 0$ as $y\rightarrow \pm \infty$. A second difference is that in the proof of v=0 on $S^+$, we multiply (3.34) by *v* and integrate by parts to get

$$\begin{aligned} \lim _{d\rightarrow \infty }\int _{S^+\cap (\mathbb {T}_L\times [-d,d])}\left( -|\nabla v|^2+{\partial _y \gamma +\beta \over u-c} |v|^2\right) dxdy=0. \end{aligned}$$

Here, we use $\lim \limits _{S^+\ni (x,y)\rightarrow (x_0,y_0)} (v\partial v)(x,y)=0$ for $(x_0,y_0)\in S^0$ a boundary point of $S^+$, and $\lim \limits _{y\rightarrow \pm \infty } (v\partial _y v)(x,y)=0$ for $x\in \mathbb {T}_L$, due to (4.1) and $v\in \dot{W}^{2,\infty }(D_L^\infty )$. This, along with $\partial _y\gamma +\beta <0$ and u>c on $S^+$, implies v=0 on $S^+$.

Under the assumption (ii), we use the method in Theorem 3.1 (ii). The main difference is that, instead of (3.12), we use the asymptotic behavior (4.6) as $y\rightarrow \pm \infty$ for $x\in \mathbb {T}_L$. □

## Applications to Monotone Shear Flows

In this section, based on the theoretical framework developed in Sects. 3-4, we present a precise classification of the traveling waves near a monotone shear flow. In an unbounded channel, we get a simple description of the propagation speeds of nearby traveling waves in Corollary 5.1. In the bounded channel setting, the situation becomes complicated and we present a comprehensive analysis of nearby traveling waves. First, we obtain the rigidity of nearby traveling waves with wave speeds lying inside the range of the monotone shear flow. Under the Rayleigh stability condition, we provide a complete characterization of the (β,L)-parameter regimes that result in either the rigidity of traveling waves for arbitrary wave speeds, or the emergence of genuine unidirectional traveling waves that are close to the monotone shear flow. In the absence of Rayleigh stability condition, we identify a parameter regime for (β,L) that allows for the existence of genuine unidirectional traveling waves in a neighborhood of the monotone shear flow. Outside this parameter regime, the wave speed of any genuine nearby traveling wave must be a generalized inflection value of the zonal velocity, see Theorem 5.10 and Remark 5.11. Our results hold for general monotone shear flows and in the special case of the Couette flow we improve Theorem 1.1 and Theorem 1.3 in [50]; see Remark 5.4 and Corollary 5.15.

Under the Rayleigh stability condition, while monotone shear flows are isolated in an unbounded channel, in a bounded channel genuine traveling waves arise near such shear flows for a large (β,L)-parameter regime. Moreover, for (β,L) in this regime, the monotone shear flow is nonlinearly Lyapunov stable yet asymptotically unstable.

### Monotone Shear Flows in An Unbounded Channel

We consider monotone shear flows $(u_0,0)$ satisfying $|u_0'|\ge C_0$ on $\mathbb {R}$ for some $C_0>0$, a typical example being the Couette flow. A characteristic of such shear flows is that the range of zonal velocity spans the entire real line, which allows us to deal with arbitrary wave speeds.

#### Corollary 5.1

Assume that $u_0\in C^2(\mathbb {R})$, and there exist $C_0, C_1>0$ such that $|u_0'|\ge C_0$ on $\mathbb {R}$ and $\left| {u_0'\over u_0}\right| \le C_1$ for |*y*| sufficiently large. Let $\varepsilon _0\in (0,C_0)$. Then the wave speed of any genuine traveling-wave solution $(u(x-ct,y),v(x-ct,y))\in C^2(D_L^{\infty })$ to the β-plane equation (2.1), subject to  (4.1) satisfying $v\in \dot{W}^{1,\infty }(D_L^{\infty })$ and

$$\begin{aligned} \Vert u-u_{0}\Vert _{W^{1,\infty }(D_L^{\infty })}<\varepsilon _0 \end{aligned}$$

must be a generalized inflection value of *u*.

#### Proof

This is a consequence of Theorem 4.3. Indeed, since $\varepsilon _0\in (0,C_0)$ and $|u_0'|\ge C_0$ on $\mathbb {R}$, we have

5.1 $$\begin{aligned} |\partial _{y}u|\ge |u_0'|-|u_0'-\partial _{y}u|>C_0-\varepsilon _0>0. \end{aligned}$$

Moreover,

5.2 $$\begin{aligned}&\left| {\partial _y u\over u}\right| \le \left| {u_0'\over u_0}-{\partial _y u\over u}\right| +\left| {u_0'\over u_0}\right| \le \left| { u_0'-\partial _y u\over u}\right| \nonumber \\&\quad +\left| {u_0'\over u_0}\cdot { u-u_0\over u}\right| +\left| {u_0'\over u_0}\right| \le \varepsilon _0+ C_1\varepsilon _0+C_1 \end{aligned}$$

for $x\in \mathbb {T}_L$ and |*y*| sufficiently large, in which case |u|>1. By (4.1), (5.1) and (5.2), all the assumptions in Theorem 4.3 hold for (*u*, *v*). Then *c* is a generalized inflection value of *u*. □

#### Remark 5.2

We point out an interesting consequence of Corollary 5.1: under the assumption of Corollary 5.1, if $\beta \notin \overline{Ran(u_0'')}$ and $\varepsilon _0$ is taken smaller such that

$$\begin{aligned} 0<\varepsilon _0<\min \left\{ C_0,\left| (u_0'')_{\inf }-\beta \right| ,\left| (u_0'')_{\sup }-\beta \right| \right\} , \end{aligned}$$

then any traveling-wave solution $(u(x-ct,y),v(x-ct,y))\in C^2(D_L^{\infty })$ to the β-plane equation (2.1), subject to (4.1) satisfying $v\in \dot{W}^{1,\infty }(D_L^{\infty })$ and

$$\begin{aligned} \Vert u-u_{0}\Vert _{W^{2,\infty }(D_L^{\infty })}<\varepsilon _0 \end{aligned}$$

must be a shear flow for arbitrary $c\in \mathbb {R}$.

In fact, since $\beta \notin \overline{Ran(u_0'')}$, we have

$$\begin{aligned} |\Delta u\!-\!\beta |\ge |u_0''-\beta |-|\Delta u-u_0''|\!\ge \!\min \left\{ \left| (u_0'')_{\inf }-\beta \right| ,\left| \beta \!-\! (u_0'')_{\sup }\right| \right\} -\varepsilon _0>0 \end{aligned}$$

for $(x,y)\in D_L^\infty$, which implies that $\beta \notin Ran(\Delta u)$. By Corollary 5.1, (u(x-ct,y),v(x-ct,y)) is a shear flow.

This shows that for a general monotone shear flow $(u_0,0)$ in an unbounded channel, if $\beta \notin \overline{Ran(u_0'')}$, genuine nearby traveling waves are absent. This is a necessary condition for asymptotic stability (i.e. nonlinear inviscid damping) near a shear flow in a specific space. Taking for example the Couette flow, it is shown in [4] that nonlinear inviscid damping near it holds in a certain Gevrey space for β=0. This result was extended to the case β>0 in [50]. Applying the above consequence to the Couette flow, we conclude that there are no genuine traveling waves $W^{2,\infty }(D_L^{\infty })$-close (in velocity) to (*y*, 0) for β>0. This is consistent with Theorem 1.7 in [50].

### Monotone Shear Flows in a Bounded Channel

We first establish a rigidity result for traveling waves near a monotone shear flow $(u_0,0)$ with wave speeds inside $Ran (u_0)$.

#### Corollary 5.3

Let $u_0\in C^2([-d,d])$ and $\delta \in (0,{u_{0,\max }-u_{0,\min }\over 2})$. Assume that $|u_0'|\ge C_0$ on [-d,d] for some $C_0>0$, $\beta \notin Ran(u_0'')$, and $c\in [u_{0,\min }+\delta ,u_{0,\max }-\delta ]$. Let

$$\begin{aligned} 0<\varepsilon _\delta <\min \{\delta ,C_0,|(u_0'')_{\min }-\beta |,|(u_0'')_{\max }-\beta |\}. \end{aligned}$$

Then any traveling-wave solution $(u(x-ct,y),v(x-ct,y))\in C^2(D_L)$ to the β-plane equation (2.1)-(2.2) satisfying

5.3 $$\begin{aligned} \Vert (u,v)-(u_{0},0)\Vert _{C^{2}(D_L)}<\varepsilon _\delta \end{aligned}$$

must be a shear flow.

#### Proof

This is a consequence of Theorem 3.1. Indeed, since $\beta \notin Ran(u_0'')$, by (5.3) and the choice of $\varepsilon _\delta$ we have $\beta \notin Ran(\Delta u)$. Moreover, $|\partial _{y}u|\ge C_0-\varepsilon _\delta >0$ for $(x,y)\in D_L.$ Let $(x_1,y_1),(x_2,y_2)\in D_L$ such that

$$\begin{aligned} u_{0}(x_1,y_1)=u_{0,\min }\quad \text {and}\quad u_{0}(x_2,y_2)=u_{0,\max }. \end{aligned}$$

Since $c\in [u_{0,\min }+\delta ,u_{0,\max }-\delta ]$, by (5.3) we have

$$\begin{aligned} c-u_{\min }\ge c-u(x_1,y_1)= c-u_0(x_1,y_1)+u_0(x_1,y_1)-u(x_1,y_1)\ge \delta -\varepsilon _\delta >0, \end{aligned}$$

and

$$\begin{aligned} c-u_{\max }\le c-u(x_2,y_2)= c-u_0(x_2,y_2)+u_0(x_2,y_2)-u(x_2,y_2)\le -\delta +\varepsilon _\delta <0. \end{aligned}$$

Thus $c\in (u_{\min },u_{\max })$ and all assumptions in Theorem 3.1 hold for (*u*, *v*). Therefore (u(x-ct,y),v(x-ct,y)) is a shear flow. □

#### Remark 5.4

Corollary 5.3 extends Theorem 1.1 in [50] from the Couette flow setting (in which case $Ran(u_0'')=\{0\}$) to general monotone shear flows, while simultaneously improving the regularity assumption from $H^{\ge 5}$ to $C^2$.

Regarding Corollary 5.3, two natural questions arise: the first is whether there are genuine nearby traveling waves outside the range $[u_{0,\min }+\delta ,u_{0,\max }-\delta ]$ for $\beta \notin Ran(u_0'')$, the other regards specific features of traveling waves for $\beta \in Ran(u_0'')$. We will provide answers in Theorem 5.10. To proceed, we first discuss some relevant spectral aspects. The principal eigenvalue of the singular Rayleigh-Kuo boundary value problem

5.4 $$\begin{aligned} -\phi ''-{\beta -u_0''\over u_0-u_{0,\min }}\phi =\lambda \phi , \,\,\,\,\phi (\pm d)=0 \end{aligned}$$

with $\phi \in H_0^1\cap H^2(-d,d)$, denoted by $\lambda _1(\beta ,u_{0,\min })$, is

5.5 $$\begin{aligned} \lambda _1(\beta ,u_{0,\min }) =&\, \inf _{\phi \in H_0^1, \Vert \phi \Vert _{L^2}=1}\int _{-d}^{d}\left( |\phi '|^2-{\beta -u_0''\over u_0-u_{0,\min }}|\phi |^2\right) dy. \end{aligned}$$

By the compactness of the embedding $H_0^1(-d,d)\hookrightarrow L^2(-d,d)$ and since $|u_0'|\ge C_0$ on [-d,d] for some $C_0>0$, $\lambda _1(\beta ,u_{0,\min })$ is well-defined and the infimum in (5.5) is attained by a corresponding eigenfunction. The variational characterization (5.5) shows that

5.6 $$\begin{aligned} \lambda _1(\cdot ,u_{0,\min })\text { is decreasing on }\mathbb {R} \text { and }\lim _{\beta \rightarrow \infty }\lambda _1(\beta ,u_{0,\min })=-\infty . \end{aligned}$$

For $c<u_{0,\min }$ we denote by $\lambda _1(\beta ,c)$ the principal eigenvalue of the regular Rayleigh-Kuo boundary value problem (5.4) with $u_{0,\min }$ replaced by *c*. When we need to specify the dependence of $\lambda _1(\beta ,c)$ on the shear-flow profile and on the zonal-band width, we denote it by $\lambda _1(\beta ,c;u_0)$ or $\lambda _1(\beta ,c;u_0,d)$, for $c\le u_{0,\min }$.

We first prove the continuity of $\lambda _1(\cdot ,u_{0,\min })$.

#### Lemma 5.5

Assume that $u_0\in C^2([-d,d])$ and $u_0'\ne 0$ on [-d,d]. Then $\lambda _1(\cdot ,u_{0,\min })$ is continuous on $\mathbb {R}$.

#### Proof

Without loss of generality, we may assume that $u_{0}'\ge C_0$ on [-d,d] for some $C_0>0$. Fix $\beta _0\in \mathbb {R}$. First, we prove that

5.7 $$\begin{aligned} \lim _{\beta \rightarrow \beta _{0}^{-}}\lambda _1(\beta ,u_{0,\min })=\lambda _1(\beta _{0},u_{0,\min }). \end{aligned}$$

Let $\beta <\beta _0$ and $\phi _{\beta _{0}}\in H_0^1(-d,d)$ with $\Vert \phi _{\beta _{0}}\Vert _{L^2(-d,d)}=1$ such that

5.8 $$\begin{aligned} \lambda _1(\beta _{0},u_{0,\min }) =&\, \int _{-d}^{d}\left( |\phi '_{\beta _{0}}|^2-{\beta _{0}-u_0''\over u_0-u_{0,\min }}|\phi _{\beta _{0}}|^2\right) dy. \end{aligned}$$

By (5.5) we have

5.9 $$\begin{aligned} \lambda _1(\beta ,u_{0,\min }) \le&\int _{-d}^{d}\left( |\phi '_{\beta _{0}}|^2-{\beta -u_0''\over u_0-u_{0,\min }}|\phi _{\beta _{0}}|^2\right) dy. \end{aligned}$$

Since $u_{0}'\ge C_0>0$, we get

5.10 $$\begin{aligned} u_{0}(y)-u_{0,\min }=u_{0}(y)-u_{0}(-d)\ge C_0(y+d)\ge 0 \end{aligned}$$

for y∈[-d,d]. By (5.6), (5.8)-(5.10) and the Hardy inequality (3.21), we obtain

$$\begin{aligned} 0&<\lambda _1(\beta ,u_{0,\min })-\lambda _1(\beta _{0},u_{0,\min }) \le \int _{-d}^{d}{\beta _0-\beta \over u_0-u_{0,\min }}|\phi _{\beta _{0}}|^2dy\\&\le \int _{-d}^{d}{\beta _0-\beta \over C_0(y+d)}|\phi _{\beta _{0}}(y)|^2dy \le {\beta _0-\beta \over C_0}\left( \int _{-d}^{d}\left| {\phi _{\beta _{0}}(y)\over y+d}\right| ^{2}dy\right) ^{\frac{1}{2}}\Vert \phi _{\beta _{0}}\Vert _{L^2(-d,d)}\\&\le C(\beta _0-\beta )\Vert \phi '_{\beta _{0}}\Vert _{L^2(-d,d)}. \end{aligned}$$

This implies that $\lambda _1(\beta ,u_{0,\min })\rightarrow \lambda _1(\beta _{0},u_{0,\min })$ as $\beta \rightarrow \beta _{0}^{-}$.

Next, we show that

5.11 $$\begin{aligned} \lim _{\beta \rightarrow \beta _{0}^{+}}\lambda _1(\beta ,u_{0,\min })=\lambda _1(\beta _{0},u_{0,\min }). \end{aligned}$$

Let $\beta >\beta _0$ and $\phi _{\beta }\in H_0^1(-d,d)$ with $\Vert \phi _{\beta }\Vert _{L^2(-d,d)}=1$ such that

5.12 $$\begin{aligned} \lambda _1(\beta ,u_{0,\min }) =&\, \int _{-d}^{d}\left( |\phi '_{\beta }|^2-{\beta -u_0''\over u_0-u_{0,\min }}|\phi _{\beta }|^2\right) dy. \end{aligned}$$

Then by (5.5) we have

$$\begin{aligned} \lambda _1(\beta _0,u_{0,\min }) \le&\int _{-d}^{d}\left( |\phi '_{\beta }|^2-{\beta _0-u_0''\over u_0-u_{0,\min }}|\phi _{\beta }|^2\right) dy. \end{aligned}$$

Thus

5.13 $$\begin{aligned} 0&>\lambda _1(\beta ,u_{0,\min })-\lambda _1(\beta _{0},u_{0,\min }) \ge \int _{-d}^{d}{\beta _0-\beta \over u_0-u_{0,\min }}|\phi _{\beta }|^2dy\nonumber \\&\ge \int _{-d}^{d}{\beta _0-\beta \over C_0(y+d)}|\phi _{\beta }(y)|^2dy \ge {\beta _0-\beta \over C_0}\left( \int _{-d}^{d}\left| {\phi _{\beta }(y)\over y+d}\right| ^{2}dy\right) ^{\frac{1}{2}}\Vert \phi _{\beta }\Vert _{L^2(-d,d)}\nonumber \\&\ge C(\beta _0-\beta )\Vert \phi '_{\beta }\Vert _{L^2(-d,d)}. \end{aligned}$$

We claim that $\Vert \phi '_{\beta }\Vert _{L^2(-d,d)}, \beta \in [\beta _{0},\beta _0+1]$ is uniformly bounded. In fact, by (5.6) we have

5.14 $$\begin{aligned} \lambda _1(\beta _0+1,u_{0,\min })\le \lambda _1(\beta ,u_{0,\min })\le \lambda _1(\beta _0,u_{0,\min }),\qquad \beta \in [\beta _{0},\beta _0+1]. \end{aligned}$$

From (5.12), (5.14), (5.10) and the fact that $\Vert \phi _{\beta }\Vert _{L^2(-d,d)}=1$ we get

$$\begin{aligned} \Vert \phi '_{\beta }\Vert _{L^2(-d,d)}^2&=\lambda _1(\beta ,u_{0,\min })\Vert \phi _{\beta }\Vert _{L^2(-d,d)}^{2} +\int _{-d}^{d}{\beta -u''_{0}\over u_0-u_{0,\min }}|\phi _{\beta }|^2dy\\&\le C|\lambda _1(\beta _0,u_{0,\min })|\Vert \phi _{\beta }\Vert _{L^2(-d,d)}\Vert \phi '_{\beta }\Vert _{L^2(-d,d)}\\&\quad +C\int _{-d}^{d}{1\over C_0(y+d)}|\phi _{\beta }(y)|^2dy\\&\le C|\lambda _1(\beta _0,u_{0,\min })|\Vert \phi '_{\beta }\Vert _{L^2(-d,d)} +C\left( \int _{-d}^{d}\left| {\phi _{\beta }(y)\over y+d}\right| ^2dy\right) ^{{1\over 2}}\\&\le C|\lambda _1(\beta _0,u_{0,\min })|\Vert \phi '_{\beta }\Vert _{L^2(-d,d)} +C\Vert \phi '_{\beta }\Vert _{L^2(-d,d)}. \end{aligned}$$

This yields

5.15 $$\begin{aligned} \Vert \phi '_{\beta }\Vert _{L^2(-d,d)}\le C|\lambda _1(\beta _0,u_{0,\min })|+C. \end{aligned}$$

It follows from (5.13) and (5.15) that

$$\begin{aligned} 0>\lambda _1(\beta ,u_{0,\min })-\lambda _1(\beta _{0},u_{0,\min })&\ge C(\beta _0-\beta )\Vert \phi '_{\beta }\Vert _{L^2(-d,d)}\\&\ge C(\beta _0-\beta )(C|\lambda _1(\beta _0,u_{0,\min })|+C)\,, \end{aligned}$$

which implies $\lambda _1(\beta ,u_{0,\min })\rightarrow \lambda _1(\beta _{0},u_{0,\min })$ as $\beta \rightarrow \beta _{0}^{+}$.

Combining (5.7) and (5.11) proves that $\lambda _1(\cdot ,u_{0,\min })$ is continuous on $\mathbb {R}$. □

We now establish the continuous dependence of the principal eigenvalue $\lambda _1(\beta ,u_{0,\min }; u_0)$ on the shear-flow profile in the $C^2$-topology.

#### Lemma 5.6

Assume that $u_0\in C^2([-d,d])$ and $u_0'\ne 0$ on [-d,d]. Then

5.16 $$\begin{aligned} \lambda _1(\beta ,\tilde{u}_{0,\min }; \tilde{u}_0)\rightarrow \lambda _1(\beta ,u_{0,\min }; u_0)\qquad \text {as}\quad \tilde{u}_0 \rightarrow u_{0}\,\, \text {in}\,\, C^2([-d,d]). \end{aligned}$$

#### Proof

Without loss of generality, we may assume that $u_0'>0$ on [-d,d]. Then there exist $C_2>C_1>0$ such that

$$\begin{aligned} C_1(y+d)\le \tilde{u}_0(y)-\tilde{u}_{0,\min }=\tilde{u}_0(y)-\tilde{u}_0(-d)\le C_2 (y+d),\qquad y\in [-d,d] \end{aligned}$$

for $\tilde{u}_0$ sufficiently close to $u_0$ in $C^2([-d,d])$, which implies

$$\begin{aligned} \left| {\beta -\tilde{u}_0''\over \tilde{u}_0-\tilde{u}_{0,\min }}\right| \le {C\over y+d},\qquad y\in [-d,d]. \end{aligned}$$

Here, the constants $C_1, C_2, C$ are independent of the choice of $\tilde{u}_0$. For any $\phi \in H_0^1(-d,d)$ with $\Vert \phi \Vert _{L^2(-d,d)}=1$, we have

5.17 $$\begin{aligned} \int _{-d}^{d}\left( |\phi '|^2-{C\over y+d}|\phi |^2\right) dy\le&\int _{-d}^{d}\left( |\phi '|^2-{\beta -\tilde{u}_0''\over \tilde{u}_0-\tilde{u}_{0,\min }}|\phi |^2\right) dy\nonumber \\ \le&\int _{-d}^{d}\left( |\phi '|^2+{C\over y+d}|\phi |^2\right) dy \end{aligned}$$

provided that $\tilde{u}_0$ is sufficiently close to $u_0$ in $C^2([-d,d])$. Taking the infimum over all $\phi \in H_0^1(-d,d)$ with $\Vert \phi \Vert _{L^2(-d,d)}=1$, we obtain

$$\begin{aligned} \lambda _1(C,-d;y)\le \lambda _1(\beta ,\tilde{u}_{0,\min }; \tilde{u}_0)\le \lambda _1(-C,-d;y) \end{aligned}$$

uniformly for $\tilde{u}_0$ sufficiently close to $u_0$ in $C^2([-d,d])$. Let $\tilde{\phi }_0\in H_0^1(-d,d)$ be an eigenfunction of $\lambda _1(\beta ,\tilde{u}_{0,\min }; \tilde{u}_0)$ with $\Vert \tilde{\phi }_0\Vert _{L^2(-d,d)}=1$. By the variational characterization of $\lambda _1(\beta ,\tilde{u}_{0,\min }; \tilde{u}_0)$ and by the Poincaré and Hardy inequalities, we have

$$\begin{aligned} \Vert \tilde{\phi }'_{0}\Vert _{L^2(-d,d)}^2&\le C \max \{|\lambda _1(C,-d;y)|,|\lambda _1(-C,-d;y)|\} \Vert \tilde{\phi }'_{0}\Vert _{L^2(-d,d)}\\&+\int _{-d}^{d}{C\over y+d}|\tilde{\phi }_{0}|^2dy\\&\le C( \max \{|\lambda _1(C,-d;y)|,|\lambda _1(-C,-d;y)|\}+1) \Vert \tilde{\phi }'_{0}\Vert _{L^2(-d,d)}, \end{aligned}$$

which yields

5.18 $$\begin{aligned} \Vert \tilde{\phi }'_{0}\Vert _{L^2(-d,d)}&\le C( \max \{|\lambda _1(C,-d;y)|,|\lambda _1(-C,-d;y)|\}+1)\triangleq M_0 \end{aligned}$$

uniformly for $\tilde{u}_0$ sufficiently close to $u_0$ in $C^2([-d,d])$. If $\phi _0\in H_0^1(-d,d)$ is an eigenfunction of $\lambda _1(\beta , u_{0,\min }; u_0)$ with $\Vert \phi _0\Vert _{L^2(-d,d)}=1$, then, by (5.18), we have

$$\begin{aligned} 0\longleftarrow&-C \Vert \tilde{u}_0-u_0\Vert _{C^2([-d,d])}M_0^2\\ \le&-C \Vert \tilde{u}_0-u_0\Vert _{C^2([-d,d])}\Vert \tilde{\phi }_0'\Vert _{L^2(-d,d)}^2\\ \le&\int _{-d}^d{(\tilde{u}_0''-u_0'')(u_0-u_{0,\min })+(\beta -u_0'')(\tilde{u}_0-u_0-(\tilde{u}_{0,\min }-u_{0,\min }))\over (\tilde{u}_0-\tilde{u}_{0,\min })( u_0-u_{0,\min })}|\tilde{\phi }_0|^2 dy\\ =&\, \int _{-d}^d\left( |\tilde{\phi }_0'|^2-{\beta -\tilde{u}_0''\over \tilde{u}_0-\tilde{u}_{0,\min }}|\tilde{\phi }_0|^2\right) dy-\int _{-d}^d\left( |\tilde{\phi }_0'|^2-{\beta - u_0''\over u_0- u_{0,\min }}|\tilde{\phi }_0|^2\right) dy\\ \le&\lambda _1(\beta ,\tilde{u}_{0,\min }; \tilde{u}_0)-\lambda _1(\beta ,u_{0,\min }; u_0)\\ \le&\int _{-d}^d\left( |\phi _0'|^2-{\beta -\tilde{u}_0''\over \tilde{u}_0-\tilde{u}_{0,\min }}|\phi _0|^2\right) dy-\int _{-d}^d\left( |\phi _0'|^2-{\beta - u_0''\over u_0- u_{0,\min }}|\phi _0|^2\right) dy\\ =&\, \int _{-d}^d{-(\beta -\tilde{u}_0'')(u_0-u_{0,\min })+(\beta -u_0'')(\tilde{u}_0-\tilde{u}_{0,\min })\over (\tilde{u}_0-\tilde{u}_{0,\min })( u_0-u_{0,\min })}|\phi _0|^2 dy\\ =&\, \int _{-d}^d{(\tilde{u}_0''-u_0'')(u_0-u_{0,\min })+(\beta -u_0'')(\tilde{u}_0-u_0-(\tilde{u}_{0,\min }-u_{0,\min }))\over (\tilde{u}_0-\tilde{u}_{0,\min })( u_0-u_{0,\min })}|\phi _0|^2 dy\\ \le&C \Vert \tilde{u}_0-u_0\Vert _{C^2([-d,d])}\int _{-d}^d{1\over (y+d)^2}|\phi _0|^2 dy\\ \le&C \Vert \tilde{u}_0-u_0\Vert _{C^2([-d,d])}\Vert \phi _0'\Vert _{L^2(-d,d)}^2\longrightarrow 0 \end{aligned}$$

since $\tilde{u}_0\rightarrow u_0$ in $C^2([-d,d])$. This proves (5.16). □

It is useful to determine the limits of the principal eigenvalue $\lambda _1(\beta ,c)$ as $c\rightarrow -\infty$ and as *c* approaches the left endpoint of the range of $u_0$.

#### Lemma 5.7

Assume that $u_0\in C^2([-d,d])$ and $u_0'\ne 0$ on [-d,d]. Then

5.19 $$\begin{aligned} \lim _{c\rightarrow u_{0,\min }^-}\lambda _1(\beta ,c)=\lambda _1(\beta ,u_{0,\min })\quad \text {and}\quad \lim _{c\rightarrow -\infty }\lambda _1(\beta ,c)={\pi ^2\over 4d^2}>0. \end{aligned}$$

#### Proof

Since the second limit in (5.19) is straightforward, we only provide a proof for the first. Assume that $u_0'>0$ on [-d,d]. For $c\in (-\infty ,u_{0,\min }]$, we have

$$\begin{aligned} u_0(y)-c&=u_0(y)-u_{0,\min }+u_{0,\min }-c\ge u_0(y)-u_{0,\min }\\&= u_0(y)-u_{0}(-d)\ge C(y+d)\,,\quad y\in [-d,d]\,. \end{aligned}$$

It follows that

$$\begin{aligned} \left| {\beta - u_0''\over u_0-c}\right| \le {C\over y+d},\qquad y\in [-d,d], \end{aligned}$$

where C>0 is independent of $c\in (-\infty ,u_{0,\min }]$. By the same argument as in (5.17), we obtain the uniform eigenvalue-bound

$$\begin{aligned} \lambda _1(C,-d;y)\le \lambda _1(\beta ,c)\le \lambda _1(-C,-d;y),\qquad c\in (-\infty ,u_{0,\min }]. \end{aligned}$$

This, in turn, yields a uniform eigenfunction-bound

$$\begin{aligned} \Vert \phi '_{c}\Vert _{L^2(-d,d)}&\le M_0,\qquad c\in (-\infty ,u_{0,\min }], \end{aligned}$$

where $\phi _c\in H_0^1(-d,d)$ is an eigenfunction of $\lambda _1(\beta ,c)$ normalized by $\Vert \phi _c\Vert _{L^2(-d,d)}=1$, and $M_0$ is defined in (5.18). Then

$$\begin{aligned} 0\longleftarrow&C(c-u_{0,\min })M_0^2\\ \le&C(c-u_{0,\min })\Vert \phi _{c}'\Vert _{L^2(-d,d)}^2\\ \le&C(c-u_{0,\min })\int _{-d}^d{1\over (y+d)^2}|\phi _{c}|^2 dy\\ \le&\int _{-d}^d\left( |\phi _{c}'|^2-{\beta -u_0''\over u_0-c}|\phi _{c}|^2\right) dy -\int _{-d}^d\left( |\phi _{c}'|^2-{\beta -u_0''\over u_0-u_{0,\min }}|\phi _{c}|^2\right) dy\\ \le&\lambda _1(\beta ,c)-\lambda _1(\beta ,u_{0,\min })\\ \le&\int _{-d}^d\left( |\phi _{u_{0,\min }}'|^2-{\beta -u_0''\over u_0-c}|\phi _{u_{0,\min }}|^2\right) dy\\&-\int _{-d}^d\left( |\phi _{u_{0,\min }}'|^2-{\beta -u_0''\over u_0-u_{0,\min }}|\phi _{u_{0,\min }}|^2\right) dy\\ =&\, (u_{0,\min }-c)\int _{-d}^d{\beta -u_0''\over (u_0-c)(u_0-u_{0,\min })}|\phi _{u_{0,\min }}|^2 dy\\ \le&C(u_{0,\min }-c)\int _{-d}^d{1\over (y+d)^2}|\phi _{u_{0,\min }}|^2 dy\\ \le&C(u_{0,\min }-c)\Vert \phi _{u_{0,\min }}'\Vert _{L^2(-d,d)}^2\longrightarrow 0 \end{aligned}$$

since $c\rightarrow u_{0,\min }^-$. This establishes the first limit in (5.19). □

Due to (5.5), we have $\lambda _1(\beta ,u_{0,\min })>0$ for $\beta <(u_0'')_{\min }$. This, together with (5.6) and Lemma 5.5, implies that the equation $\lambda _1(\cdot ,u_{0,\min })=0$ has a unique solution in $[(u_0'')_{\min },\infty )$, denoted by $\lambda _1^{-1}(0,u_{0,\min })$. We now prove the positiveness of $\lambda _1(0,u_{0,\min })$.

#### Lemma 5.8

Assume that $u_0\in C^2([-d,d])$ and $u_0'\ne 0$ on [-d,d]. Then $\lambda _1(0,u_{0,\min })>0$, and consequently, $\lambda _1^{-1}(0,u_{0,\min })>0$.

#### Proof

Without loss of generality, we may assume that $u_0'\ge C_0$ on [-d,d] for some $C_0>0$. Let $\phi _0\in H_0^1(-d,d)$ with $\Vert \phi _0\Vert _{L^2(-d,d)}=1$ such that (5.12) holds with β=0. Then

$$\begin{aligned} \lambda _1(0,u_{0,\min })=&\, \int _{-d}^{d}\left( |\phi '_{0}|^2+{u_0''\over u_0-u_{0,\min }}|\phi _{0}|^2\right) dy\\ =&\, \int _{-d}^{d}|\phi '_{0}|^2dy+{u_0'\phi _0^2\over u_0-u_0(-d)}\bigg |_{y=-d}^d-\int _{-d}^du_0'\left( {\phi _0^2\over u_0-u_{0,\min }}\right) 'dy. \end{aligned}$$

Since

$$\begin{aligned}&\left| {u_0'(y)\phi _0(y)^2\over u_0(y)-u_0(-d)}\right| \le {|u_0'(y)|\Vert \phi _0'\Vert _{L^2(-d,y)}^2(y+d)\over C_0(y+d)}\rightarrow 0\quad \text {as}\quad y\rightarrow -d, \end{aligned}$$

and

$$\begin{aligned}&\int _{-d}^d{\phi _0\phi _0'u_0'\over u_0-u_{0,\min }}dy\le C\Vert \phi _0'\Vert _{L^2(-d,d)}^2,\,\,\int _{-d}^d{\phi _0^2u_0'^2\over (u_0-u_{0,\min })^2}dy\le C\Vert \phi _0'\Vert _{L^2(-d,d)}^2, \end{aligned}$$

we have

$$\begin{aligned} \lambda _1(0,u_{0,\min })=&\, \int _{-d}^{d}\left( |\phi '_{0}|^2-{2\phi _0\phi _0'u_0'\over u_0-u_{0,\min }}+{\phi _0^2u_0'^2\over (u_0-u_{0,\min })^2}\right) dy\\ =&\, \int _{-d}^{d}\left( \phi '_{0}-{\phi _0u_0'\over u_0-u_{0,\min }}\right) ^2dy\ge 0. \end{aligned}$$

Suppose that $\lambda _1(0,u_{0,\min })=0$. Then $\phi '_{0}={\phi _0u_0'\over u_0-u_{0,\min }}$ for y∈(-d,d). Thus $|\phi _0|=C(u_0-u_{0,\min })$ on (-d,d) and $0=|\phi _0(d)|=C(u_0(d)-u_0(-d))$. This implies that C=0 and $\phi _0\equiv 0$ on (-d,d), which contradicts $\Vert \phi _0\Vert _{L^2(-d,d)}=1$. Therefore, $\lambda _1(0,u_{0,\min })>0$. □

#### Remark 5.9

In Lemma 5.8, if we replace the monotone shear flow $u_0$ by a non-monotone shear flow $u_0$ such that $u_0$ attains its minimum only at the endpoints ±d and $u_0'(\pm d)\ne 0$, then $\lambda _1(0,u_{0,\min })\ge 0$. Moreover, $\lambda _1(0,u_{0,\min })=0$ can be achieved by considering the shear flow $u_0(y)=\cos \left( {\pi \over 2} y\right)$ on [-1,1]. Indeed, it can be directly verified that $\cos \left( {\pi \over 2} y\right)$ is an eigenfunction of the principal eigenvalue $\lambda _1(0,u_{0,\min })=0$ for the eigenvalue problem (5.4).

Now we are ready to completely characterize traveling waves near a monotone shear flow in a bounded channel.

#### Theorem 5.10

Let $u_0\in C^2([-d,d])$ and $u_0'\ne 0$ on [-d,d].

- Let β>0.
  For $$\beta \notin Ran(u_0'')$$,
  (i) if $L\in \left( 0,{2\pi \over \sqrt{\max \{-\lambda _1(\beta ,u_{0,\min }),0\}}}\right)$, then there exists $\varepsilon _0>0$ such that any traveling-wave solution $(u(x-ct,y),v(x-ct,y))\in C^2(D_L)$ to the β-plane equation (2.1)-(2.2) satisfying $\Vert (u,v)-(u_{0},0)\Vert _{C^{2}(D_L)}$ $<\varepsilon _0$ must be a shear flow for arbitrary wave speed $c\in \mathbb {R}$;
  (ii) if $L\in \left[ {2\pi \over \sqrt{\max \{-\lambda _1(\beta ,u_{0,\min }),0\}}},\infty \right)$, then for any ε>0 there exists a genuine traveling-wave solution $(u_\varepsilon (x-c_\varepsilon t,y),v_\varepsilon (x-c_\varepsilon t,y))\in C^2(D_{L})$ to the β-plane equation (2.1)-(2.2) satisfying $\Vert (u_\varepsilon ,v_\varepsilon )-(u_{0},0)\Vert _{C^{2}(D_{L})}<\varepsilon$ and $c_\varepsilon <(u_\varepsilon )_{\min }.$
  For $$\beta \in Ran(u_0'')$$,
  (iii) if $L\in \left( 0,{2\pi \over \sqrt{\max \left\{ -\inf _{\tilde{c}\le u_{0,\min }}\lambda _1(\beta ,\tilde{c}),0\right\} }}\right)$, then there exists $\varepsilon _0>0$ such that the wave speed of any genuine traveling-wave solution $(u(x-ct,y),v(x-ct,y))\in C^2(D_L)$ to the β-plane equation (2.1)-(2.2) satisfying $\Vert (u,v)-(u_{0},0)\Vert _{C^{2}(D_L)}$ $<\varepsilon _0$ must be a generalized inflection value of *u*;
  (iv) if $L\in \left[ {2\pi \over \sqrt{\max \left\{ -\inf _{\tilde{c}\le u_{0,\min }}\lambda _1(\beta ,\tilde{c}),0\right\} }},\infty \right)$, then the same conclusion in $(\textrm{ii})$ holds, with the convention that [a,b)=∅ if a≥b.
- Let β=0.
  If $0\notin Ran(u_0'')$, then the same conclusion as in (i) holds.
  If $0\in Ran(u_0'')$, then the same conclusion as in (iii) holds.

#### Proof

Let β>0. We prove the following equivalent statements for $\beta \notin Ran(u_0'')$.

1. Assume that $0<(u_0'')_{\min }$. Let $\beta \in (0,(u_0'')_{\min })$ and L>0 be arbitrary. Then there exists $\varepsilon _0>0$ such that any traveling-wave solution $(u(x-ct,y),v(x-ct,y))\in C^2(D_L)$ to the β-plane equation (2.1)-(2.2) satisfying $\Vert (u,v)-(u_{0},0)\Vert _{C^{2}(D_L)}$ $<\varepsilon _0$ must be a shear flow for an arbitrary wave speed $c\in \mathbb {R}$.
2. Assume that $$\lambda _1((u_0'')_{\max },u_{0,\min })>0$$.
  - Let $\beta \in ((u_0'')_{\max },\lambda _1^{-1}(0,u_{0,\min })]\cap (0,\infty )$ and L>0 be arbitrary. Then the same conclusion as in (1) holds.
  - Let $\beta \in (\lambda _1^{-1}(0,u_{0,\min }),\infty )$. (Note that $0< \lambda _1^{-1}(0,u_{0,\min })$.)
    $\mathrm{(2b_1)}$ If $L\in \left( 0,{2\pi \over \sqrt{-\lambda _1(\beta ,u_{0,\min })}}\right)$, then the same conclusion as in $\mathrm{(1)}$ holds.
    $\mathrm{(2b_2)}$ If $L\in \left[ {2\pi \over \sqrt{-\lambda _1(\beta ,u_{0,\min })}},\infty \right)$, then for any ε>0 there exists a genuine traveling-wave solution $(u_\varepsilon (x-c_\varepsilon t,y),v_\varepsilon (x-c_\varepsilon t,y))\in C^2(D_{L})$ to the β-plane equation (2.1)-(2.2) satisfying $\Vert (u_\varepsilon ,v_\varepsilon )-(u_{0},0)\Vert _{C^{2}(D_{L})}<\varepsilon$ and $c_\varepsilon <(u_\varepsilon )_{\min }.$
3. Assume that $\lambda _1((u_0'')_{\max },u_{0,\min })\le 0$. Let $\beta \in ((u_0'')_{\max },\infty )$. (Note that $0< \lambda _1^{-1}(0,u_{0,\min })\le (u_0'')_{\max }$.)
  - If $L\in \left( 0,{2\pi \over \sqrt{-\lambda _1(\beta ,u_{0,\min })}}\right)$, then the same conclusion as in $\mathrm{(1)}$ holds.
  - If $L\in \left[ {2\pi \over \sqrt{-\lambda _1(\beta ,u_{0,\min })}},\infty \right)$, then the same conclusion as in $\mathrm{(2b_2)}$ holds.
  We prove the following equivalent statements for $\beta \in Ran(u_0'')$:
4. Assume that $\inf _{\tilde{c}\le u_{0,\min }}\lambda _1(\beta ,\tilde{c})\ge 0$. Let L>0 be arbitrary. Then there exists $\varepsilon _0>0$ such that the wave speed of any genuine traveling-wave solution $(u(x-ct,y),v(x-ct,y))\in C^2(D_L)$ to the β-plane equation (2.1)-(2.2) satisfying $\Vert (u,v)-(u_{0},0)\Vert _{C^{2}(D_L)}$ $<\varepsilon _0$ must be a generalized inflection value of *u*.
5. Assume that $$\inf _{\tilde{c}\le u_{0,\min }}\lambda _1(\beta ,\tilde{c})<0$$.
  - If $L\in \left( 0,{2\pi \over \sqrt{-\inf _{\tilde{c}\le u_{0,\min }}\lambda _1(\beta ,\tilde{c})}}\right)$, then the same conclusion as in $\mathrm{(4)}$ holds.
  - If $L\in \left[ {2\pi \over \sqrt{-\inf _{\tilde{c}\le u_{0,\min }}\lambda _1(\beta ,\tilde{c})}},\infty \right)$, then the same conclusion as in $\mathrm{(2b_2)}$ holds.

**Proof of (1).** Since $\beta \in (0,(u_0'')_{\min })$, we can choose $\varepsilon _0\in (0,1)$ small enough such that $\beta <(\Delta u)_{\min }$ on $D_L$. By Theorem 3.12, (u(x-ct,y),v(x-ct,y)) is a shear flow for all $c\in \mathbb {R}$.

**Proof of (2).** By (5.6), we have

5.20 $$\begin{aligned}&\lambda _1(\beta ,u_{0,\min })>0\quad \text {for} \quad \beta \in ((u_0'')_{\max },\lambda _1^{-1}(0,u_{0,\min })), \nonumber \\  &\lambda _1(\beta ,u_{0,\min })<0\quad \text {for} \quad \beta \in (\lambda _1^{-1}(0,u_{0,\min }),\infty ). \end{aligned}$$

**Proof of (2a).** Without loss of generality, we assume that $$u_0'>0$$ on [-d,d]. Since $$\beta >(u_0'')_{\max }$$ and $$u_0'>0$$ on [-d,d], we can choose $$\varepsilon _0\in (0,1)$$ small enough such that $$\beta > (\Delta u)_{\max }$$ and $$ \partial _{y}u> 0$$ on $$D_L. $$

Let $\beta \in ((u_0'')_{\max },\lambda _1^{-1}(0,u_{0,\min })]\cap (0,\infty )$ and L>0 be arbitrary. Since β>0, by Theorem 3.10 (i) we know that (u(x-ct,y),v(x-ct,y)) is a shear flow for $c\notin [c_\beta ^+,u_{\min }]$, where $c_\beta ^+$ is defined in (1.5) with $d_{\pm }=\pm d$. Now, let (u(x-ct,y),v(x-ct,y)) be a traveling wave with $c\in [c_\beta ^+,u_{\min }]$. We will show that it has to be a shear flow. Since $\partial _y u\ge C_0$ on $D_L$ for some $C_0>0$, we have

5.21 $$\begin{aligned} u(x,y)-c\ge u(x,y)-u_{\min }\ge u(x,y)-u(x,-d)\ge C_0(y+d)\end{aligned}$$

for $$(x,y)\in D_L$$. Moreover,

$$\begin{aligned} \Vert \beta -\Delta u\Vert _{C^0(D_L)}\le \Vert \beta -u_0''\Vert _{C^0(D_L)}+\Vert u_0''-\Delta u\Vert _{C^0(D_L)}\le C+\varepsilon _0\le C'\, \end{aligned}$$

for some $C^{\prime }>C$. By (3.8), (5.21), v(·,-d)=0 and the Hardy inequality (3.21), we have

$$\begin{aligned}&\Vert \nabla v\Vert _{L^2(D_L)}^2=\int _{D_L}|\nabla v|^2 dxdy \le C\int _{D_L}{1\over u-c}v^2dxdy\\&\quad \le C\int _{D_L}{v^2\over y+d} dxdy \le C\Vert v\Vert _{L^2(D_L)}\left( \int _{\mathbb {T}_L}\int _{-d}^d{v^2\over (y+d)^2} dydx\right) ^{1\over 2}\\&\quad \le C_1\Vert v\Vert _{L^2(D_L)}\left( \int _{\mathbb {T}_L}\int _{-d}^d|\partial _y v(x,y)|^2 dydx\right) ^{1\over 2} \le C_1\Vert v\Vert _{L^2(D_L)}\Vert \nabla v\Vert _{L^2(D_L)}, \end{aligned}$$

which implies

5.22 $$\begin{aligned} \Vert \nabla v\Vert _{L^2(D_L)} \le C_1\Vert v\Vert _{L^2(D_L)} \end{aligned}$$

for some $C_1>0$. Since $\beta >(\Delta u)_{\max }$, by (5.21) we have

5.23 $$\begin{aligned} 0=&\, \int _{D_L}\left( |\nabla v|^2 -{\beta -\Delta u\over u-c}v^2\right) dxdy\nonumber \\ \ge&\int _{D_L}\left( |\nabla v|^2 -{\beta -\Delta u\over u-u(x,-d)}v^2\right) dxdy\nonumber \\ =&\, \int _{D_L}\left( |\nabla v|^2-{\beta - u_0''\over u_0-u_0(-d)}v^2\right) dxdy\nonumber \\&+\int _{D_L}\left( {\beta - u_0''\over u_0-u_0(-d)}v^2 -{\beta -\Delta u\over u-u(x,-d)}v^2\right) dxdy\nonumber \\ =&\, I+II. \end{aligned}$$

Since $\beta \in ((u_0'')_{\max },\lambda _1^{-1}(0,u_{0,\min })]$, by (5.20) we have $\lambda _1(\beta ,u_{0,\min })\ge 0$ and

$$\begin{aligned} \int _{-d}^d\left( |\phi '|^2-{\beta -u_0''\over u_0-u_{0,\min }}|\phi |^2\right) dy\ge \lambda _1(\beta ,u_{0,\min })\int _{-d}^d|\phi |^2dy\ge 0 \end{aligned}$$

for $0\ne \phi \in H_0^1(-d,d)$. Then $v(x,y)=\sum _{k\ne 0} e^{i{2k\pi \over L}x} v_k(y)$ and

$$\begin{aligned} I=&\, L\sum _{k\ne 0}\int _{-d}^d\left( |v_k'|^2+k^2\left( {2\pi \over L}\right) ^2|v_k|^2-{\beta -u_0''\over u_0-u_{0,\min }}|v_k|^2\right) dy\\ \ge&L\sum _{k\ne 0}\int _{-d}^d\left( \lambda _1(\beta ,u_{0,\min })+k^2\left( {2\pi \over L}\right) ^2\right) |v_k|^2dy\\ \ge&\left( {2\pi \over L}\right) ^2\Vert v\Vert _{L^2(D_L)}^2\ge {\left( {2\pi / L}\right) ^2\over C_1^2}\Vert \nabla v\Vert _{L^2(D_L)}^2, \end{aligned}$$

where we have used (5.22) and $\int _{\mathbb {T}_L} v dx=0$. On the other hand, by (5.21) and $u_0(y)-u_0(-d)\ge \tilde{C}_0(y+d)$ for some $\tilde{C}_0>0$, we have

5.24 $$\begin{aligned} -II=&\, \int _{D_L}\bigg ( {\beta -\Delta u\over u-u(x,-d)}v^2-{\beta - u_0''\over u-u(x,-d)}v^2+{\beta - u_0''\over u-u(x,-d)}v^2\nonumber \\&-{\beta - u_0''\over u_0-u_0(-d)}v^2\bigg )dxdy\nonumber \\ =&\, \int _{D_L}\left( {u_0''-\Delta u\over u-u(x,-d)}v^2+{(\beta - u_0'')(u_0-u-u_0(-d)+u(x,-d))\over (u-u(x,-d))(u_0-u_0(-d))}v^2\right) dxdy\nonumber \\ \le&C\Vert u_0- u\Vert _{C^2(D_L)}\int _{D_L}{1\over (y+d)}v^2 dxdy+C\Vert u_0- u\Vert _{C^0(D_L)}\int _{D_L}{1\over (y+d)^2}v^2 dxdy\nonumber \\ \le&C\Vert u_0-u\Vert _{C^2(D_L)}\Vert v\Vert _{L^2(D_L)}\Vert \nabla v\Vert _{L^2(D_L)}+C\Vert u_0- u\Vert _{C^0(D_L)}\Vert \nabla v\Vert _{L^2(D_L)}^2\nonumber \\ \le&C_2\Vert u_0-u\Vert _{C^2(D_L)}\Vert \nabla v\Vert _{L^2(D_L)}^2 \end{aligned}$$

for some $C_2>0$, where we have used v(·,-d)=0 and the Hardy and Poincaré inequalities in the last two estimates. Combining the estimates for *I* and *II*, we obtain

$$\begin{aligned} {\left( {2\pi / L}\right) ^2\over C_1^2}\Vert \nabla v\Vert _{L^2(D_L)}^2\le I\le -II\le C_2\Vert u_0-u\Vert _{C^2(D_L)}\Vert \nabla v\Vert _{L^2(D_L)}^2. \end{aligned}$$

Taking $\varepsilon _0>0$ such that $\varepsilon _0<{\left( {2\pi / L}\right) ^2\over C_1^2C_2}$, we get ∇v=0 and v=0 on $D_L$, proving $(\textrm{2a})$.

**Proof of** $\boldsymbol{\mathrm {(2b_1)}}$. Let $\beta \in (\lambda _1^{-1}(0,u_{0,\min }),\infty )$ and $L\in \left( 0,{2\pi \over \sqrt{-\lambda _1(\beta ,u_{0,\min })}}\right)$. Then $\beta>\lambda _1^{-1}(0,u_{0,\min })>0$ by Lemma 5.8. By Theorem 3.10 (i), we only need to show that if (u(x-ct,y),v(x-ct,y)) is a traveling wave with $c\in [c_\beta ^+,u_{\min }]$, then it must be a shear flow. We still have (5.22), and define *I* and *II* by (5.23). Since $\beta \in (\lambda _1^{-1}(0,u_{0,\min }),\infty )$, by (5.20) and $L\in \left( 0,{2\pi \over \sqrt{-\lambda _1(\beta ,u_{0,\min })}}\right)$, we have

5.25 $$\begin{aligned} 0>\lambda _1(\beta ,u_{0,\min })>-\left( 2\pi \over L\right) ^2 \end{aligned}$$

and

5.26 $$\begin{aligned} \int _{-d}^d\left( |\phi '|^2-{\beta -u_0''\over u_0-u_{0,\min }}|\phi |^2\right) dy\ge \lambda _1(\beta ,u_{0,\min })\int _{-d}^d|\phi |^2dy \end{aligned}$$

for $0\ne \phi \in H_0^1(-d,d)$. Then by (5.26) we obtain

5.27 $$\begin{aligned} I \ge&L\sum _{k\ne 0}\int _{-d}^d\left( \lambda _1(\beta ,u_{0,\min })+\left( {2\pi \over L}\right) ^2\right) |v_k|^2dy\nonumber \\&+L\sum _{k\ne 0}\int _{-d}^d\left( -\left( {2\pi \over L}\right) ^2+k^2\left( {2\pi \over L}\right) ^2\right) |v_k|^2dy\nonumber \\ \ge&L\left( \lambda _1(\beta ,u_{0,\min })+\left( {2\pi \over L}\right) ^2\right) \sum _{k\ne 0}\int _{-d}^d|v_k|^2dy\nonumber \\ =&\, \left( \lambda _1(\beta ,u_{0,\min })+\left( {2\pi \over L}\right) ^2\right) \Vert v\Vert _{L^2(D_L)}^2 \ge {\lambda _1(\beta ,u_{0,\min })+\left( {2\pi \over L}\right) ^2\over C_1^2}\Vert \nabla v\Vert _{L^2(D_L)}^2, \end{aligned}$$

where $v(x,y)=\sum _{k\ne 0} e^{i{2k\pi \over L}x} v_k(y)$ and we have used (5.22). Note that ${\lambda _1(\beta ,u_{0,\min })+\left( {2\pi \over L}\right) ^2\over C_1^2}>0$ due to (5.25). The estimates (5.24) for *II* still hold. By (5.27) and (5.24) we have

$$\begin{aligned} {\lambda _1(\beta ,u_{0,\min })+\left( {2\pi \over L}\right) ^2\over C_1^2}\Vert \nabla v\Vert _{L^2(D_L)}^2\le I\le -II\le C_2\Vert u_0-u\Vert _{C^2(D_L)}\Vert \nabla v\Vert _{L^2(D_L)}^2. \end{aligned}$$

Taking $\varepsilon _0<{\lambda _1(\beta ,u_{0,\min })+\left( {2\pi \over L}\right) ^2\over C_1^2C_2}$, we conclude that v=0 on $D_L$. This proves $(\mathrm {2b_1})$.

**Proof of** $\boldsymbol{\mathrm {(\mathrm {2b_2})}}$. Let $\beta \in (\lambda _1^{-1}(0,u_{0,\min }),\infty )$. We first consider the case $L > {2\pi \over \sqrt{-\lambda _1(\beta ,u_{0,\min };u_0)}}$, where $\lambda _1(\beta ,u_{0,\min };u_0)<0$ by (5.20). Then

$$\begin{aligned} \lambda _1(\beta , u_{0,\min };u_0)<-\left( {2\pi \over L}\right) ^2<0\, \end{aligned}$$

This, combined with Lemma 5.7, implies that there exists $c_{L}<u_{0,\min }$ such that $\lambda _1(\beta ,c_{L};u_0)=-\left( {2\pi \over L}\right) ^2$. Thus $c_{L}$ is an isolated real eigenvalue of the operator

$$\begin{aligned} \mathcal {R}_{{2\pi \over L},\beta ,u_0}=-\bigg (\partial _y^2-\bigg ({2\pi \over L}\bigg )^2\bigg )^{-1}\bigg ((u_0''-\beta )-u_0\bigg (\partial _y^2-\bigg ({2\pi \over L}\bigg )^2\bigg )\bigg ). \end{aligned}$$

Indeed, to simplify notation, set $A_L={\partial _y^2}-\left( {2\pi \over L}\right) ^2$. A direct computation yields the commutator identity

$$\begin{aligned} A_L(u_0\phi )-u_0A_L\phi =u_0''\phi +2u_0'\phi ', \end{aligned}$$

and hence

$$\begin{aligned} A_L^{-1}u_0A_L\phi =u_0\phi -A_L^{-1}(u_0''\phi +2u_0'\phi '). \end{aligned}$$

Substituting this into the definition of $\mathcal {R}_{{2\pi \over L},\beta ,u_0}$, we obtain

$$\begin{aligned} \mathcal {R}_{{2\pi \over L},\beta ,u_0}\phi =u_0\phi -A_L^{-1}(-\beta \phi +2(u_0'\phi )') \end{aligned}$$

for $\phi \in L^2(-d,d)$. Since the mapping $\phi \mapsto -\beta \phi +2(u_0'\phi )'$ is bounded from $L^2(-d,d)$ to $H^{-1}(-d,d)$, and $A_L^{-1}:H^{-1}(-d,d)\rightarrow H_0^1(-d,d)$ is bounded, the compact embedding $H_0^1(-d,d)\hookrightarrow L^2(-d,d)$ implies that the operator $\phi \mapsto -A_L^{-1}(-\beta \phi +2(u_0'\phi )')$ is compact on $L^2(-d,d)$. It follows that $\mathcal {R}_{{2\pi \over L},\beta ,u_0}$ is a compact perturbation of $M_{u_0}$, where $M_{u_0}$ denotes the multiplication operator by $u_0$. By Theorem 5.35 in Chapter IV of [32], we obtain the following spectral properties of $\mathcal {R}_{{2\pi \over L},\beta ,u_0}$:

- its essential spectrum is given by $\sigma _e\big (\mathcal {R}_{{2\pi \over L},\beta ,u_0}\big )=\sigma _e\left( M_{u_0}\right) =Ran\,(u_0)$,
- its isolated real eigenvalues lie outside the range of $u_0$, namely, $\sigma _d\big (\mathcal {R}_{{2\pi \over L},\beta ,u_0}\big )\cap \mathbb {R}\subset (-\infty ,u_{0,\min })\cup (u_{0,\max },\infty )$.

Any eigenvalue contained in $Ran\,(u_0)$ belongs to the essential spectrum and is therefore non-isolated.

Although the spectral condition $c_{L}\in \sigma _d(\mathcal {R}_{{2\pi \over L},\beta ,u_0})\cap \mathbb {R}$ is satisfied, we can not directly apply the bifurcation lemma (Corollary 2.6 in [33]) to the shear flow $(u_0,0)$ since it requires the shear flow to possess $H^4$ regularity, whereas we only have $u_0\in C^2.$ Our approach is to perturb $(u_0,0)$ to a nearby shear flow $(\tilde{u}_0,0)$ with $C^4$ regularity. The only subtlety that remains is then to ensure that the corresponding operator $\mathcal {R}_{{2\pi \over L},\beta ,\tilde{u}_0}$ retains an isolated real eigenvalue. As we will see, this obstacle is readily resolved by Lemmas 5.6-5.7. Indeed, for any ε>0, by Lemma 5.6 and the density of $C^\infty ([-d,d])$ in $C^2([-d,d])$, we can choose $\tilde{u}_0\in C^\infty ([-d,d])$ such that $\tilde{u}_0'>0$ on [-d,d],

5.28 $$\begin{aligned} \Vert \tilde{u}_0-u_0\Vert _{C^2([-d,d])}<{\varepsilon \over 2} \end{aligned}$$

and

$$\begin{aligned} |\lambda _1(\beta ,\tilde{u}_{0,\min };\tilde{u}_0)-\lambda _1(\beta , u_{0,\min }; u_0)|<{-\left( 2\pi \over L\right) ^2-\lambda _1(\beta ,u_{0,\min };u_0)\over 2}. \end{aligned}$$

Then

5.29 $$\begin{aligned}&-\left( 2\pi \over L\right) ^2-\lambda _1(\beta ,\tilde{u}_{0,\min };\tilde{u}_0)\nonumber \\&\quad>-\left( 2\pi \over L\right) ^2-\lambda _1(\beta ,u_{0,\min };u_0)-{-\left( 2\pi \over L\right) ^2-\lambda _1(\beta ,u_{0,\min };u_0)\over 2}\nonumber \\&\quad ={-\left( 2\pi \over L\right) ^2-\lambda _1(\beta ,u_{0,\min };u_0)\over 2}>0, \end{aligned}$$

which implies

5.30 $$\begin{aligned} \lambda _1(\beta ,\tilde{u}_{0,\min };\tilde{u}_0)<-\left( {2\pi \over L}\right) ^2<0.\end{aligned}$$

By applying Lemma 5.7 to $\tilde{u}_0$, we have

5.31 $$\begin{aligned} \lim _{c\rightarrow \tilde{u}_{0,\min }^-}\lambda _1(\beta ,c;\tilde{u}_0)=\lambda _1(\beta ,\tilde{u}_{0,\min };\tilde{u}_0)<0\quad \text {and}\quad \lim _{c\rightarrow -\infty }\lambda _1(\beta ,c;\tilde{u}_0)={\pi ^2\over 4d^2}>0. \end{aligned}$$

It follows from (5.30)-(5.31) that there exists $\tilde{c}_L<\tilde{u}_{0,\min }$ such that $\lambda _1(\beta ,\tilde{c}_L;\tilde{u}_0)=-\left( {2\pi \over L}\right) ^2$. Then $\tilde{c}_L$ is an isolated real eigenvalue of the operator $\mathcal {R}_{{2\pi \over L},\beta ,\tilde{u}_0}$. We now can apply Corollary 2.6 in [33] to the shear flow $(\tilde{u}_0,0)$, and obtain the existence of a genuine traveling-wave solution $(u_\varepsilon (x-c_\varepsilon t,y),v_\varepsilon (x-c_\varepsilon t,y))\in H^4(D_{L})$ to the β-plane equation (2.1)-(2.2) satisfying $c_\varepsilon <(u_\varepsilon )_{\min }$ and

5.32 $$\begin{aligned} \Vert (u_\varepsilon ,v_\varepsilon )-(\tilde{u}_{0},0)\Vert _{C^{2}(D_{L})}\le \Vert (u_\varepsilon ,v_\varepsilon )-(\tilde{u}_{0},0)\Vert _{H^{4}(D_{L})}<{\varepsilon \over 2} \,. \end{aligned}$$

Combining (5.28) and (5.32), we conclude that $\Vert (u_\varepsilon ,v_\varepsilon )-(u_{0},0)\Vert _{C^{2}(D_{L})}<\varepsilon$.

We next consider the case $L={2\pi \over \sqrt{-\lambda _1(\beta ,u_{0,\min };u_0)}}$. In this case, the operator $\mathcal {R}_{{2\pi \over L},\beta ,u_0}$ has a non-isolated eigenvalue $u_{0,\min }$. The strategy is as follows. We first perturb the shear flow $(u_0,0)$ to a scaled shear flow $(au_0,0)$ with 0<a<1 so that the corresponding operator $\mathcal {R}_{{2\pi \over L},\beta ,a u_0}$ admits an isolated real eigenvalue. Then, as in the previous case, we further perturb $(au_0,0)$ to a shear flow $(\hat{u}_0,0)$ with $C^\infty$ regularity and apply the bifurcation lemma to $(\hat{u}_0,0)$ in order to obtain a nearby genuine traveling-wave solution. More precisely, for the shear flow $(au_0,0)$ with a∈(0,1), the corresponding Rayleigh-Kuo boundary value problem is

$$\begin{aligned} -\phi ''-{\beta -au_0''\over au_0-c}\phi =-\phi ''-{{\beta \over a}-u_0''\over u_0-{c\over a}}\phi =\lambda \phi , \,\,\,\,\phi (\pm d)=0 \end{aligned}$$

with $\phi \in H_0^1\cap H^2(-d,d)$, where $c\le au_{0,\min }$. Then

5.33 $$\begin{aligned} \lambda _{1}(\beta ,c;au_0)=\lambda _{1}\left( {\beta \over a},{c\over a};u_0\right) \end{aligned}$$

for $c\le a u_{0,\min }$. By (5.6) and ${\beta \over a}>\beta$, we have

5.34 $$\begin{aligned} -\left( 2\pi \over L\right) ^2=\lambda _1(\beta ,u_{0,\min };u_0)>\lambda _1\left( {\beta \over a},u_{0,\min };u_0\right) =\lambda _1(\beta ,au_{0,\min };au_0).\end{aligned}$$

By Lemma 5.7 and (5.34), there exists $c_{L,a}<u_{0,\min }$ such that

5.35 $$\begin{aligned} \lambda _1\left( {\beta \over a},c_{L,a};u_0\right) =-\left( 2\pi \over L\right) ^2.\end{aligned}$$

Combining (5.33) and (5.35), we have

5.36 $$\begin{aligned} \lambda _1(\beta ,ac_{L,a};au_0)=-\left( 2\pi \over L\right) ^2. \end{aligned}$$

Since $ac_{L,a}<au_{0,\min }$, $ac_{L,a}$ is an isolated real eigenvalue of the operator $\mathcal {R}_{{2\pi \over L},\beta ,au_0}$. For any ε>0, we take a∈(0,1) close to 1 such that

5.37 $$\begin{aligned} \Vert au_0-u_0\Vert _{C^2([-d,d])}<{\varepsilon \over 3}.\end{aligned}$$

By (5.36), Lemma 5.6 and the density of $C^\infty ([-d,d])$ in $C^2([-d,d])$, we can choose $\hat{u}_0\in C^\infty ([-d,d])$ such that $\hat{u}_0'>0$ on [-d,d],

5.38 $$\begin{aligned} \Vert \hat{u}_0-au_0\Vert _{C^2([-d,d])}<{\varepsilon \over 3} \end{aligned}$$

and

$$\begin{aligned} |\lambda _1(\beta ,\hat{u}_{0,\min };\hat{u}_0)-\lambda _1(\beta , au_{0,\min }; au_0)|<{-\left( 2\pi \over L\right) ^2-\lambda _1(\beta ,au_{0,\min };au_0)\over 2}. \end{aligned}$$

By an argument similar to that in (5.29), we have

5.39 $$\begin{aligned} \lambda _1(\beta ,\hat{u}_{0,\min };\hat{u}_0)<-\left( {2\pi \over L}\right) ^2<0. \end{aligned}$$

Applying Lemma 5.7 to $\hat{u}_0$ and using (5.39), there exists $\hat{c}_L<\hat{u}_{0,\min }$ such that $\lambda _1(\beta ,\hat{c}_L;\hat{u}_0)=-\left( {2\pi \over L}\right) ^2$. Thus $\hat{c}_{L}$ is an isolated real eigenvalue of the operator $\mathcal {R}_{{2\pi \over L},\beta ,\hat{u}_0}$. Applying Corollary 2.6 in [33] to the shear flow $(\hat{u}_0,0)$, we obtain the existence of a genuine traveling-wave solution $(u_\varepsilon (x-c_\varepsilon t,y),v_\varepsilon (x-c_\varepsilon t,y))\in C^2(D_L)$ such that $c_\varepsilon <(u_\varepsilon )_{\min }$ and

5.40 $$\begin{aligned} \Vert (u_\varepsilon ,v_\varepsilon )-(\hat{u}_0,0)\Vert _{C^2(D_L)}<{\varepsilon \over 3}\,.\end{aligned}$$

Combining (5.37)-(5.38) and (5.40), we conclude that $\Vert (u_\varepsilon ,v_\varepsilon )-( u_0,0)\Vert _{C^2(D_L)}$ <ε. This proves $(\mathrm {2b_2})$.

**Proof of (3).** By (5.6) we have

$$\begin{aligned} \lambda _1(\beta ,u_{0,\min })<0\quad \text {for} \quad \beta \in ((u_0'')_{\max },\infty ). \end{aligned}$$

Let $\beta \in ((u_0'')_{\max },\infty )$. If $L\in \left( 0,{2\pi \over \sqrt{-\lambda _1(\beta ,u_{0,\min })}}\right)$, then $\lambda _1(\beta ,c)\ge \lambda _1(\beta ,u_{0,\min })>-(2\pi /L)^2$ for all $c\le u_{0,\min }$. If $L\in \left[ {2\pi \over \sqrt{-\lambda _1(\beta ,u_{0,\min })}},\infty \right)$, then there exists $c_{L}\le u_{0,\min }$ such that $\lambda _1(\beta ,c_{L})=-(2\pi /L)^2$. Based on the above spectral properties, the proofs of $(\textrm{3a})$ and $(\textrm{3b})$ are similar to those of $(\mathrm{2b_1})$ and $(\mathrm{2b_2})$, respectively.

**Proof of** $\boldsymbol{\mathrm {(4)}}$ **and** $\boldsymbol{\mathrm {(5a)}}$. Suppose that *c* is not a generalized inflection value of *u*. We show that (u(x-ct,y),v(x-ct,y)) is a shear flow when $\varepsilon _0>0$ is small enough. Note that *c* is not a critical value of *u* since *u* is $C^2$ close to the monotone shear flow $u_0$. If $c=u_{\max }$, by Remark 3.6(u(x-ct,y),v(x-ct,y)) is a shear flow. If $c\notin [c_\beta ^+,u_{\min }]$, by Theorem 3.3 (i) (u(x-ct,y),v(x-ct,y)) is a shear flow. Now let (u(x-ct,y),v(x-ct,y)) be a traveling wave with $c\in [c_\beta ^+,u_{\min }]$. We will show that it has to be a shear flow. We still have (5.22) for some $C_1>0$. We divide the discussions into two cases.

In the case $c\le u_{0,\min }$, we have

$$\begin{aligned} 0&=\int _{D_L}\left( |\nabla v|^2-{\beta -u_0''\over u_0-c}v^2\right) dxdy\\&\quad +\int _{D_L}\left( {\beta -u_0''\over u_0-c}v^2-{\beta -\Delta u\over u-c}v^2\right) dxdy=I_0+II_0. \end{aligned}$$

For (4), we have $\lambda _1(\beta ,c)\ge \inf _{\tilde{c}\le u_{0,\min }}\lambda _1(\beta ,\tilde{c})\ge 0>-\left( {2\pi \over L}\right) ^2$. For $\mathrm {(5a)}$, we have $\lambda _1(\beta ,c)\ge \inf _{\tilde{c}\le u_{0,\min }}\lambda _1(\beta ,\tilde{c})>-\left( {2\pi \over L}\right) ^2$. Using (5.22), we get

$$\begin{aligned} I_0=&\, L\sum _{k\ne 0}\int _{-d}^d\left( |v_k'|^2+k^2\left( {2\pi \over L}\right) ^2|v_k|^2-{\beta -u_0''\over u_0-c}|v_k|^2\right) dy\\ \ge&\left( \lambda _1(\beta ,c)+\left( {2\pi \over L}\right) ^2\right) \Vert v\Vert _{L^2(D_L)}^2 \ge {\inf _{\tilde{c}\le u_{0,\min }}\lambda _1(\beta ,\tilde{c})+\left( {2\pi \over L}\right) ^2\over C_1^2}\Vert \nabla v\Vert _{L^2(D_L)}^2. \end{aligned}$$

Since $c\le u_{\min }$ and $c\le u_{0,\min }$, similarly to (5.24) we obtain

$$\begin{aligned} -II_0=&\, \int _{D_L}\left( {u_0''-\Delta u\over u-c}v^2+{(\beta - u_0'')(u_0-u)\over (u-c)(u_0-c)}v^2\right) dxdy\\&\le C_2\Vert u_0-u\Vert _{C^2(D_L)}\Vert \nabla v\Vert _{L^2(D_L)}^2 \end{aligned}$$

for some $C_2>0$. By the estimates for $I_0$ and $II_0$, we have

$$\begin{aligned} {\inf _{\tilde{c}\le u_{0,\min }}\lambda _1(\beta ,\tilde{c})+\left( {2\pi \over L}\right) ^2\over C_1^2}\Vert \nabla v\Vert _{L^2(D_L)}^2\le I_0=-II_0\le C_2\Vert u_0-u\Vert _{C^2(D_L)}\Vert \nabla v\Vert _{L^2(D_L)}^2. \end{aligned}$$

Then v=0 if $$\varepsilon _0< {\inf _{\tilde{c}\le u_{0,\min }}\lambda _1(\beta ,\tilde{c})+\left( {2\pi \over L}\right) ^2\over C_1^2C_2}$$.

In the other case, $c>u_{0,\min }$, we have $u_0(-d)=u_{0,\min }<c\le u_{\min }=u(x_0,-d)$ for some $x_0\in \mathbb {T}_L$. Then

5.41 $$\begin{aligned} |c-u_{0,\min }|+|c-u_{\min }|=u_{\min }-u_{0,\min }=u(x_0,-d)-u_0(-d)\le \Vert u-u_0\Vert _{C^0(D_L)} \end{aligned}$$

and

$$\begin{aligned} 0&=\int _{D_L}\left( |\nabla v|^2-{\beta -u_0''\over u_0-u_{0,\min }}v^2\right) dxdy+\int _{D_L}\left( {\beta -u_0''\over u_0-u_{0,\min }}v^2-{\beta -\Delta u\over u-c}v^2\right) dxdy\\&=I_1+II_1. \end{aligned}$$

For both (4) and $\mathrm{(5a)}$, we have $\lambda _1(\beta ,u_{0,\min })\ge \inf _{\tilde{c}\le u_{0,\min }}\lambda _1(\beta ,\tilde{c})>-\left( {2\pi \over L}\right) ^2$. Then

$$\begin{aligned} I_1=&\, L\sum _{k\ne 0}\int _{-d}^d\left( |v_k'|^2+k^2\left( {2\pi \over L}\right) ^2|v_k|^2-{\beta -u_0''\over u_0-u_{0,\min }}|v_k|^2\right) dy\\ \ge&\left( \lambda _1(\beta ,u_{0,\min })+\left( {2\pi \over L}\right) ^2\right) \Vert v\Vert _{L^2(D_L)}^2 \ge {\inf _{\tilde{c}\le u_{0,\min }}\lambda _1(\beta ,\tilde{c})+\left( {2\pi \over L}\right) ^2\over C_1^2}\Vert \nabla v\Vert _{L^2(D_L)}^2. \end{aligned}$$

Using (5.21) and (5.41), we obtain

$$\begin{aligned} -II_1=&\, \int _{D_L}{(\beta -\Delta u)(u_0-u_{0,\min })-(\beta -u_0'')(u-c)\over (u_0-u_{0,\min })(u-c)}v^2dxdy\\ \le&\int _{D_L}{|((\beta -\Delta u)-(\beta -u_0''))(u_0-u_{0,\min })+(\beta -u_0'')((u_0-u_{0,\min })-(u-c))|\over (u_0-u_{0,\min })(u-c)}v^2dxdy\\ \le&\int _{D_L}{|u_0''-\Delta u||u_0-u_{0,\min }|+|\beta -u_0''|(|u-u_0|+|c-u_{0,\min }|)\over (u_0-u_{0,\min })(u-c)}v^2dxdy\\ \le&C\Vert u-u_0\Vert _{C^2(D_L)}\int _{D_L}{1\over y+d}v^2dxdy+C\Vert u-u_0\Vert _{C^0(D_L)}\int _{D_L}{1\over (y+d)^2}v^2dxdy\\ \le&C\Vert u-u_0\Vert _{C^2(D_L)}\Vert v\Vert _{L^2(D_L)}\Vert \nabla v\Vert _{L^2(D_L)}+C\Vert u-u_0\Vert _{C^0(D_L)}\Vert \nabla v\Vert _{L^2(D_L)}^2\\ \le&C_3\Vert u-u_0\Vert _{C^2(D_L)}\Vert \nabla v\Vert _{L^2(D_L)}^2 \end{aligned}$$

for some $C_3>0$. By the estimates for $I_1$ and $II_1$, and choosing $\varepsilon _0< {\inf _{\tilde{c}\le u_{0,\min }}\lambda _1(\beta ,\tilde{c})+\left( {2\pi \over L}\right) ^2\over C_1^2C_3}$, we get v=0. This proves (4) and $\mathrm {(5a)}$.

**Proof of** $\boldsymbol{\mathbf {(5b)}}$. By Lemma 5.7, there exists $c_0\in (-\infty ,u_{0,\min }]$ such that

$$\begin{aligned} \lambda _1(\beta ,c_0;u_0)=\inf _{\tilde{c}\le u_{0,\min }}\lambda _1(\beta ,\tilde{c};u_0)\,. \end{aligned}$$

In the case $L > {2\pi \over \sqrt{-\inf _{\tilde{c}\le u_{0,\min }}\lambda _1(\beta ,\tilde{c};u_0)}}$, we have

$$\begin{aligned} \inf _{\tilde{c}\le u_{0,\min }}\lambda _1(\beta ,\tilde{c};u_0)< -\left( {2\pi \over L}\right) ^2<0\,. \end{aligned}$$

It then follows again from Lemma 5.7 that there exists $c_L\in (-\infty , c_0)$ such that $\lambda _1(\beta ,c_L;u_0)=-\left( {2\pi \over L}\right) ^2$. Thus $c_{L}$ is an isolated real eigenvalue of the operator $\mathcal {R}_{{2\pi \over L},\beta , u_0}$. Since $u_0\in C^2([-d,d])$ lacks the regularity required for the bifurcation, by an argument similar to the first case of $\mathrm {(2b_2)}$ we can perturb $(u_0,0)$ to a nearby shear flow $(\tilde{u}_0,0)$ with $C^\infty$ regularity, and then construct a genuine traveling-wave solution by bifurcating at $(\tilde{u}_0,0)$.

In the remaining case $L={2\pi \over \sqrt{-\inf _{\tilde{c}\le u_{0,\min }}\lambda _1(\beta ,\tilde{c};u_0)}}$, we have $-\left( {2\pi \over L}\right) ^2=\lambda _1(\beta ,c_0;u_0)$. Let a∈(0,1). Then $\lambda _1(\beta ,c;au_0)=\lambda _1\left( {\beta \over a},{c\over a};u_0\right)$ for $c\le a u_{0,\min }$ and

$$\begin{aligned} -\left( {2\pi \over L}\right) ^2=\lambda _1(\beta ,c_0;u_0)>\lambda _1\left( {\beta \over a},c_0;u_0\right) =\lambda _1(\beta , ac_0;au_0) \end{aligned}$$

due to (5.6). This, along with Lemma 5.7, implies that there exists $c_{L,a}<c_0$ such that

$$\begin{aligned} \lambda _1(\beta ,ac_{L,a};au_0)=\lambda _1\left( {\beta \over a},c_{L,a};u_0\right) =-\left( {2\pi \over L}\right) ^2\,. \end{aligned}$$

Since $ac_{L,a}<ac_0\le au_{0,\min }$, $ac_{L,a}$ is an isolated real eigenvalue of $\mathcal {R}_{{2\pi \over L},\beta , au_0}$. With these preparations, arguing as in the second case of $\mathrm {(2b_2)}$, we can first perturb $(u_0,0)$ to the scaled shear flow $(au_0,0)$, then further perturb $(au_0,0)$ to $(\hat{u}_0,0)$ with $C^\infty$ regularity, to finally generate a genuine traveling-wave solution by bifurcating from $(\hat{u}_0,0)$.

Let β=0. If $0\notin Ran(u_0'')$, then by taking $\varepsilon _0>0$ small enough, $\Delta u\ne 0$ on $D_L$. By Theorem 3.12 (ii), the same conclusion as in Theorem 5.10 (i) holds. If $0\in Ran(u_0'')$, then $0\in Ran(\Delta u)$ is possible and by Theorem 3.3 the same conclusion as in Theorem 5.10 (iii) holds. □

#### Remark 5.11

In Theorem 5.10 (iii), it is natural to ask whether there exists a monotone shear flow with genuine nearby traveling waves having wave speeds which are generalized inflection values of the zonal velocity. A concrete example is the monotone shear flow $(U_{b,a},0)$ constructed in (5.6) of [50], which satisfies $U_{b,a}'>0$ on [-1,1], $U_{b,a}(0)=0$ and $\beta =U_{b,a}''(0)$, with b>0 sufficiently small and a>0. By Theorem 1.5 in [50], there exists a genuine traveling wave (*u*(*x*, *y*), *v*(*x*, *y*)) with zero wave speed (i.e. a steady flow) near the shear flow $(U_{b,a},0)$ for some b,a>0 sufficiently small. Since (*u*, *v*) is $C^2$ close enough to the monotone shear flow $(U_{b,a},0)$, c=0∈Ran(u) and *c* is neither a critical value nor a maximum/minimum of *u*. By Theorem 3.1, c=0 must be a generalized inflection value of *u*.

#### Remark 5.12

Let us describe more precisely what Theorem 5.10 (i)-(ii) reveals for $\beta \notin Ran(u_0'')$. Assume that $u_0'\ne 0$ on $D_L$. Let *L* be the zonal period and *c* be the wave speed of a traveling wave. We distinguish the three cases.

**Case 1.** $$0<(u_0'')_{\min }$$.

In the subcase $\lambda _1((u_0'')_{\max },u_{0,\min })>0$, we have $\lambda _1^{-1}(0,u_{0,\min })>(u_0'')_{\max }>0$, and

- if $\beta \in [0,(u_0'')_{\min })\cup ((u_0'')_{\max },\lambda _1^{-1}(0,u_{0,\min })]$, then $\lambda _1(\beta ,u_{0,\min })\ge 0$ and the rigidity of traveling waves with $c\in \mathbb {R}$ holds for L>0,
- if $\beta \in (\lambda _1^{-1}(0,u_{0,\min }),\infty )$, then $\lambda _1(\beta ,u_{0,\min })<0$ and
  - the rigidity of traveling waves with $c\in \mathbb {R}$ holds for $L\in \left( 0,{2\pi \over \sqrt{-\lambda _1(\beta ,u_{0,\min })}}\right)$,
  - genuine traveling waves exist for $L\in \left[ {2\pi \over \sqrt{-\lambda _1(\beta ,u_{0,\min })}},\infty \right)$,

near $$(u_0,0)$$ in $$C^2$$.

In the other subcase, $\lambda _1((u_0'')_{\max },u_{0,\min })\le 0$, we have $(u_0'')_{\max }\ge \lambda _1^{-1}(0,u_{0,\min })>0$ (due to $\lambda _1(0,u_{0,\min })>\lambda _1((u_0'')_{\min },u_{0,\min })\ge 0$), and

- if $\beta \in [0,(u_0'')_{\min })$, then $\lambda _1(\beta ,u_{0,\min })>0$ and the rigidity of traveling waves with $c\in \mathbb {R}$ holds for L>0,
- if $\beta \in ((u_0'')_{\max },\infty )$, then $\lambda _1(\beta ,u_{0,\min })<0$ and
  - the rigidity of traveling waves with $c\in \mathbb {R}$ holds for $L\in \left( 0,{2\pi \over \sqrt{-\lambda _1(\beta ,u_{0,\min })}}\right)$,
  - genuine traveling waves exist for $L\in \left[ {2\pi \over \sqrt{-\lambda _1(\beta ,u_{0,\min })}},\infty \right)$,

near $$(u_0,0)$$ in $$C^2$$.

**Case 2.** $$0\in Ran (u_0'')$$.

In the subcase $\lambda _1((u_0'')_{\max },u_{0,\min })>0$, we have $\lambda _1^{-1}(0,u_{0,\min })>(u_0'')_{\max }\ge 0$, and

**Fig. 7:**
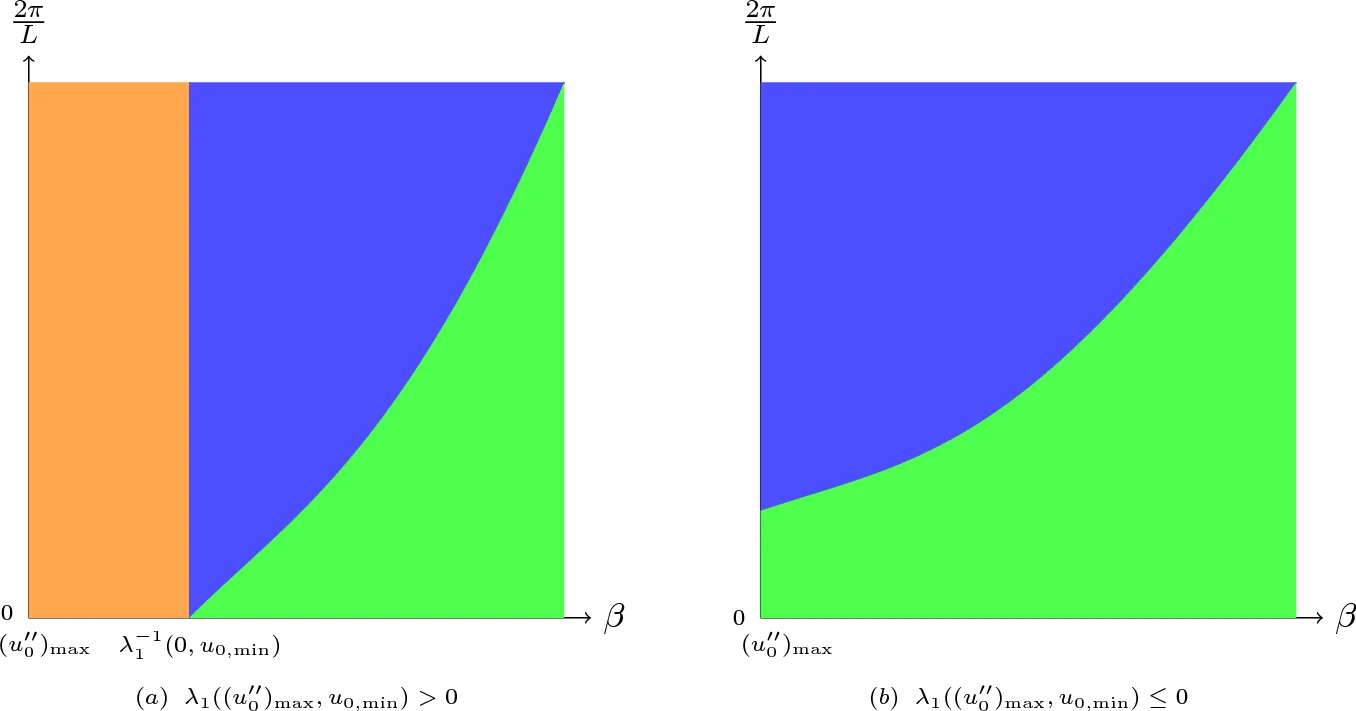
The illustration for Case 2 in Remark 5.12: near a monotone shear flow, the rigidity of traveling waves with arbitrary wave speeds holds for $(\beta ,{2\pi \over L})$ in the orange and blue regions, while genuine traveling waves exist for $(\beta ,{2\pi \over L})$ in the green regions. The boundary between the blue and green regions is the curve ${2\pi /L}=\sqrt{-\lambda _1(\beta ,u_{0,\min })}$

- if $\beta \in ((u_0'')_{\max },\lambda _1^{-1}(0,u_{0,\min })]$, then $\lambda _1(\beta ,u_{0,\min })\ge 0$ and the rigidity of traveling waves with $c\in \mathbb {R}$ holds for L>0,
- if $\beta \in (\lambda _1^{-1}(0,u_{0,\min }),\infty )$, then $\lambda _1(\beta ,u_{0,\min })<0$ and
  - the rigidity of traveling waves with $c\in \mathbb {R}$ holds for $L\in \left( 0,{2\pi \over \sqrt{-\lambda _1(\beta ,u_{0,\min })}}\right)$,
  - genuine traveling waves exist for $L\in \left[ {2\pi \over \sqrt{-\lambda _1(\beta ,u_{0,\min })}},\infty \right)$,

near $(u_0,0)$ in $C^2$, see Fig. 7 (a).

In the other subcase, $\lambda _1((u_0'')_{\max },u_{0,\min })\le 0$, by Lemma 5.8 we have $(u_0'')_{\max }\ge \lambda _1^{-1}(0,u_{0,\min })>0$, and for every $\beta \in ((u_0'')_{\max },\infty )$, $\lambda _1(\beta ,u_{0,\min })<0$ and

- the rigidity of traveling waves with $c\in \mathbb {R}$ holds for $L\in \left( 0,{2\pi \over \sqrt{-\lambda _1(\beta ,u_{0,\min })}}\right)$,
- genuine traveling waves exist for $L\in \left[ {2\pi \over \sqrt{-\lambda _1(\beta ,u_{0,\min })}},\infty \right)$,

near $(u_0,0)$ in $C^2$, see Fig. 7 (b).

**Case 3.** $$0>(u_0'')_{\max }$$.

In this case, we have $\lambda _1((u_0'')_{\max },u_{0,\min })>0$, since otherwise, $0>(u_0'')_{\max }\ge \lambda _1^{-1}(0,u_{0,\min })$, and thus $\lambda _1(0,u_{0,\min })<0$, which contradicts $\lambda _1(0,u_{0,\min })>0$ (see Lemma 5.8). Since $\lambda _1((u_0'')_{\max },u_{0,\min })>0$, by Lemma 5.8 we have $\lambda _1^{-1}(0,u_{0,\min })>0>(u_0'')_{\max }$, and

- if $\beta \in [0,\lambda _1^{-1}(0,u_{0,\min })]$, then $\lambda _1(\beta ,u_{0,\min })\ge 0$ and the rigidity of traveling waves with $c\in \mathbb {R}$ holds for L>0,
- if $\beta \in (\lambda _1^{-1}(0,u_{0,\min }),\infty )$, then $\lambda _1(\beta ,u_{0,\min })<0$ and
  - the rigidity of traveling waves with $c\in \mathbb {R}$ holds for $L\in \left( 0,{2\pi \over \sqrt{-\lambda _1(\beta ,u_{0,\min })}}\right)$,
  - genuine traveling waves exist for $L\in \left[ {2\pi \over \sqrt{-\lambda _1(\beta ,u_{0,\min })}},\infty \right)$,

near $$(u_0,0)$$ in $$C^2$$.

#### Remark 5.13

Let us compare the rigidity property of traveling waves with arbitrary wave speeds near monotone shear flows in bounded versus unbounded channels. Assume that the Rayleigh stability condition (i.e. $\beta \notin \overline{Ran(u_0'')}$) holds. In an unbounded channel, the rigidity near general monotone shear flows with appropriate asymptotic behavior is proved for arbitrary zonal periods (see Remark 5.2). In a bounded channel, however, it depends on β and on the zonal period *L*, see Theorem 5.10 and Remark 5.12. In particular, we provide critical regions of (β,L) such that the rigidity near monotone shear flows does not hold in Theorem 5.10, see the green regions of Fig. 7 for Case 2 in Remark 5.12.

#### Example 5.14

As an illustration of the physical relevance of Theorem 5.10, we consider Case 3 of Remark 5.12. The data provided in Fig. 12.5 of [42] shows the presence of steady waves (with zero speed) in the zonal band between 72$^\circ$N and 78$^\circ$N on Saturn, with an underlying shear flow $u_0(y)$ that increases poleward and has a strictly concave shape. For this zonal band we have $\beta>0>(u_0'')_{\max }$, and Theorem 5.10 ensures that only waves with sufficiently large wavelengths can arise as perturbations of this shear flow. The wavelength of the observed waves, which form Saturn’s hexagon, is indeed about 14500 km (see [9]).

Applying Theorem 5.10 to the Couette flow (i.e. $u_0(y)=y$, y∈[-1,1]) and using the fact that $\lambda _{1}^{-1}(0,-1)>0$ and $Ran(u_0'')=\{0\}$, we get the following result.

#### Corollary 5.15

Consider the Couette flow $u_0(y)=y$, y∈[-1,1].

(i) Let $\beta \in (0,\lambda _{1}^{-1}(0,-1)]$ and L>0 be arbitrary. Then the rigidity of traveling waves with $c\in \mathbb {R}$ holds near the Couette flow in $C^2$, as described in Theorem 5.10 (i).
(ii) Let $$\beta \in (\lambda _{1}^{-1}(0,-1),\infty )$$.
  $\mathrm{(ii_{a})}$ If $L\in \left( 0,\frac{2\pi }{\sqrt{-\lambda _{1}(\beta ,-1)}}\right)$, then the same conclusion as in Corollary 5.15 (i) holds.
  $\mathrm{(ii_{b})}$ If $L\in \left[ \frac{2\pi }{\sqrt{-\lambda _{1}(\beta ,-1)}},\infty \right)$, then genuine traveling waves exist near the Couette flow in $C^2$, as described in Theorem 5.10(ii).

#### Remark 5.16

In the β-plane setting, compared to Theorem 1.3 in [50], Corollary 5.15 improves the regularity requirement, reducing it from $H^{\ge 5}$ to $C^2$. In the *f*-plane setting, we mention that the rigidity of traveling waves with $c\in \mathbb {R}$ holds for L>0 near the Couette flow in $H^{>{5\over 2}}$ [35].

#### Remark 5.17

The cases 1-3 in Remark 5.12 show that when $\lambda _1((u_0'')_{\max },u_{0,\min })>0$, then the value $\lambda _1^{-1}(0,u_{0,\min })$ serves as the transitional β-parameter for the onset of genuine traveling waves near the monotone shear flow $(u_0,0)$ in $C^2$. More precisely, if $\beta \le \lambda _1^{-1}(0,u_{0,\min })$, the rigidity of nearby traveling waves holds for all wave speeds and for all zonal periods, while if $\beta >\lambda _1^{-1}(0,u_{0,\min })$, there exist genuine nearby traveling waves for some large wavelengths.

We use Matlab to numerically compute the transitional β-parameter $\lambda _1^{-1}(0,u_{0,\min })$ for monotone shear flows with either linear profiles or concave parabolic profiles. These computations illustrate how the transitional β-value varies as the zonal-band width increases or as the background shear intensifies. To emphasize the dependence on both the shear profile and the zonal-band width, we rewrite the transitional parameter as $\beta _\mathrm{{crit}}(u_0, d)=\lambda _1^{-1}(0,u_{0,\min })$.

**Linearly sheared profiles:** We first compute the transitional β-parameter for the Couette flow $u_0(y)=y$ confined to the band [-1,1]. The corresponding singular Rayleigh-Kuo boundary value problem (5.4) is

5.42 $$\begin{aligned} -\phi ''-{\beta \over y+1}\phi =\lambda \phi , \,\,\,\,\phi (\pm 1)=0. \end{aligned}$$

By numerical computation, we have $\lambda _1(1.8352,-1;y, 1)\approx 0$ and thus

$$\begin{aligned} \beta _\mathrm{{crit}}(y, 1)\approx 1.8352. \end{aligned}$$

For a general linearly sheared flow $u_0(y)=ay+b$ with ±a>0 confined to a band [-d,d], the corresponding singular Rayleigh-Kuo boundary value problem (5.4) is

5.43 $$\begin{aligned} -\phi ''-{\beta \over ay\pm ad}\phi =\lambda \phi , \,\,\,\,\phi (\pm d)=0. \end{aligned}$$

By performing the scaling $\phi (y)=\varphi (z)$ with y=±dz, (5.43) is transformed to

5.44 $$\begin{aligned} -\varphi ''-{\pm {d\over a}\beta \over z+1}\varphi =d^2\lambda \varphi , \,\,\,\,\varphi (\pm 1)=0. \end{aligned}$$

By comparing (5.44) with (5.42), we obtain

5.45 $$\begin{aligned} \beta _\mathrm{{crit}}(ay+b, d)=\pm {a\over d}\beta _\mathrm{{crit}}(y, 1)\approx \pm {a\over d}\cdot 1.8352\quad \text {for} \quad \pm a>0. \end{aligned}$$

That is, the transitional β-parameter for the linearly sheared flow $u_0(y)=ay+b$ (±a>0) on [-d,d] is a scaled version of that for the Couette flow on [-1,1]. The relation (5.45) explains the following trends for linear profiles:

(i) within a fixed zonal band, the stronger the shear effect is, the larger is the range of β for which the rigidity of traveling waves persists for all wave speeds and for all zonal periods;
(ii) for a fixed linearly sheared profile, the wider the zonal band is, the larger is the admissible range of β that allows the existence of genuine traveling waves.

**Fig. 8:**
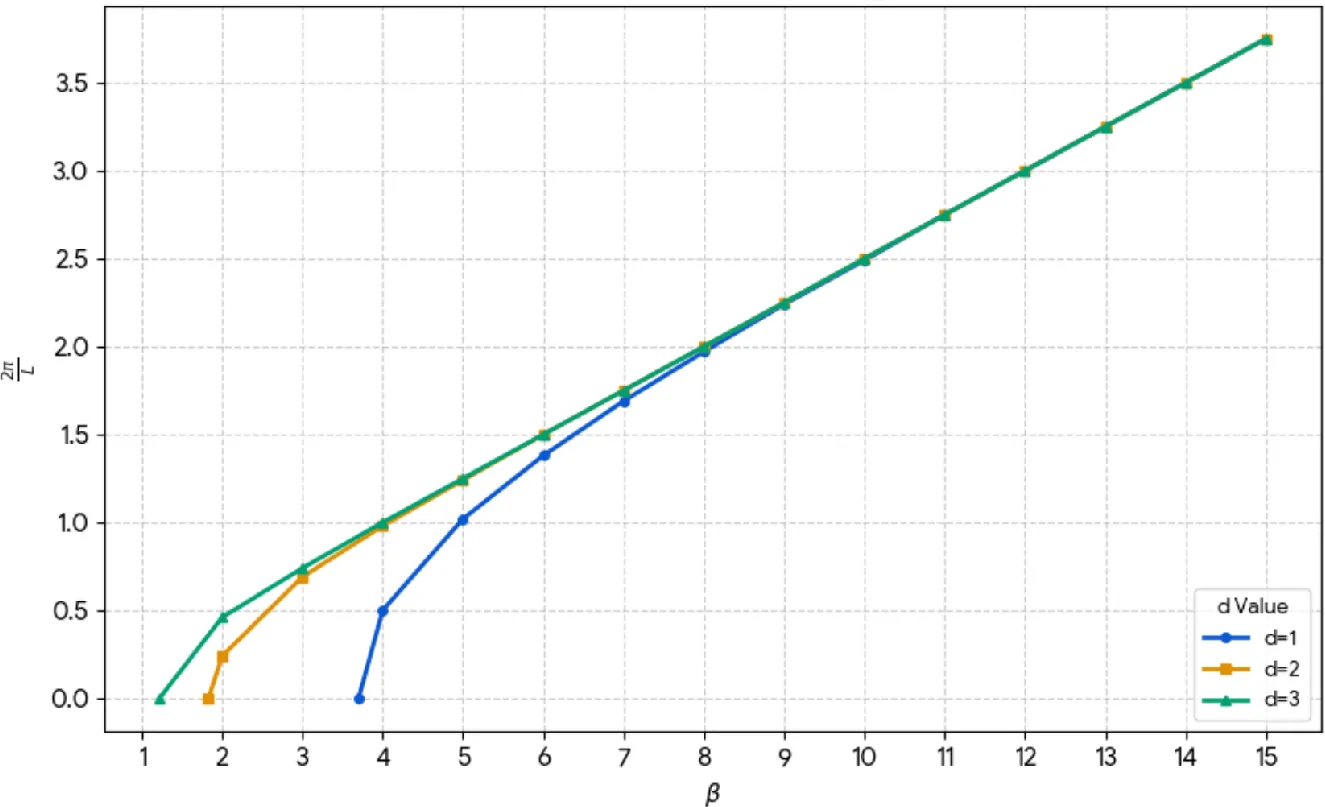
For values of β slightly above the transitional threshold, the critical wavelength depends sensitively on the zonal-band width, leading to noticeable variation among different widths. As β increases further, these critical wavelengths become gradually close to one another

Note that for a general linearly sheared flow $u_0(y)=ay+b$ (a≠0) we have $Ran(u_0'')=\{0\}$ and $\lambda _1((u_0'')_{\max },u_{0,\min })=\lambda _1(0,u_{0,\min })>0$ by Lemma 5.8. Consequently, the first subcase of Case 2 in Remark 5.12 always holds. As β exceeds the transitional value, a boundary curve emerges in the (β,L)-plane, given by ${2\pi \over L}=\sqrt{-\lambda _1(\beta ,u_{0,\min })}$, as depicted in Fig. 7 (a). This curve marks the critical wavelength for any fixed β that exceeds the transitional value: absence (existence) of genuine traveling waves holds for smaller (larger) wavelength, near $(u_0,0)$ in $C^2$. For a specific linearly sheared profile $u_0(y)=2y+3$ confined to the zonal band [-d,d], we numerically compute the critical wave number ${2\pi \over L}=\sqrt{-\lambda _1(\beta ,u_{0,\min })}, \beta >\beta _\mathrm{{crit}}(u_0, d)$ and simulate the boundary curves for d=1,2,3. The resulting computations and observations are summarized in Fig. 8.

**Concave parabolic profiles:** For the concave parabolic profiles $u_0(y)=-y^2+by+e$ confined to the zonal band [-d,d], we impose the condition $d<{b\over 2}$ to ensure that $u_0$ is monotone on this band. The corresponding Rayleigh-Kuo boundary value problem (5.4) is

5.46 $$\begin{aligned} -\phi ''+{\beta +2\over (y+d)(y-d-b)}\phi =\lambda \phi , \,\,\,\,\phi (\pm d)=0. \end{aligned}$$

Note that in a fixed zonal band, increasing *b* displaces the parabola horizontally to the right while maintaining its shape, resulting in a stronger shear effect. Numerical computation of the eigenvalues of (5.46) yields the transitional β-values listed in Table 2, which exhibit trends (i)-(ii) comparable to those observed for the linear profiles.

#### Example 5.18

We use the numerical computation of the transitional β-value in Remark 5.17 to explain the lack of waves in Jupiter’s zonal band between 37$^\circ$S and 39$^\circ$S that was noted in [39]. Indeed, the profile $u_0$ of the zonal flow in this band is linearly sheared, featuring an eastward jet with speed 45 *m*/*s* along the northern boundary and a westward jet with speed 5 *m*/*s* along the southern boundary (see Fig. 4 in [26] for accurate data). By (1.1), a bandwidth of two degrees of latitude corresponds to $2d={\pi \over 90}\approx 0.035$. Using the values listed in Table 1 together with the dimensionless velocity expression (2.6) in [10], we obtain the boundary values $u_0(-d)=-{1\over 30}$ and $u_0(d)={3\over 10}$, yielding the linear profile $u_0(y)=ay+b$ with $a={1\over 6d}$ and $b={2\over 15}$, which describes the observed flow pattern. Due to (1.1) and (5.45), we have

$$\begin{aligned} \beta ={2\times 1.76\times 10^{-4}\times 69911\times 10^3\over 150} \cos (38^\circ )\approx 129< \beta _\mathrm{{crit}}(ay+b, d) \approx 1004. \end{aligned}$$

By Theorem 5.10 and Remark 5.17, the rigidity of traveling waves holds in a $C^2$-neighborhood of $(u_0,0)$ for arbitrary wave speeds and zonal periods. This result is consistent with the absence of genuine traveling waves in the corresponding zonal band and provides theoretical support for the observed shear-flow patterns described in [39].

Under the Rayleigh stability condition, $C^2$ shear flows (not necessarily monotone) turn out to be nonlinearly Lyapunov stable (see Theorem 5.19 below). However, due to the existence of genuine nearby traveling waves, asymptotic stability (i.e. nonlinear inviscid damping) fails near a monotone shear flow $(u_0(y),0)$ for $(\beta ,L)\in \bigg (\max \bigg \{(u_0'')_{\max },$ $\lambda _1^{-1}(0,u_{0,\min })\bigg \},\infty \bigg )\times \left[ {2\pi \over \sqrt{-\lambda _1(\beta ,u_{0,\min })}},\infty \right)$. This means that in such case, the long-time dynamics near the monotone shear flow is considerably richer. The rigidity near the monotone shear flow suggests that asymptotic stability may potentially be proved in certain function spaces for other values of (β,L). In the *f*-plane setting, it is worth noting that nonlinear inviscid damping in certain Gevrey spaces has been proved near a class of monotone shear flows in [28, 37], where the Rayleigh stability condition does not hold since the shear flow $u_0$ satisfies $u_0''=0$ near the boundary and, thus $0=\beta \in Ran(u_0'')$.

**Table 2:** Numerical computation of $\beta _{crit}(-y^2+by+e,d)$

	d=1	d=2	d=3
b=7	13.2496	6.7922	4.6236
b=8	15.0898	7.7176	5.2450
b=9	16.9289	8.6416	5.8648

#### Theorem 5.19

Assume that $u_0\in C^2([-d,d])$, $u_0'\ne 0$ on [-d,d], and $(\beta ,L)\in (\max \{(u_0'')_{\max },$ $\lambda _1^{-1}(0,u_{0,\min })\},\infty )\times \left[ {2\pi \over \sqrt{-\lambda _1(\beta ,u_{0,\min })}},\infty \right)$. Then $(u_0, 0)$ is nonlinearly Lyapunov stable in (vorticity) $L^2$, but nonlinear inviscid damping fails near $(u_0, 0)$ in $C^2$.

#### Proof

First, we prove that $(u_0, 0)$ is nonlinearly Lyapunov stable in (vorticity) $L^2$ as long as $\beta \notin Ran(u_0'')$. The proof of nonlinear Lyapunov stability is valid for general $C^2$ shear flows, not necessarily the monotone ones. We use the standard method introduced by Arnold [1, 2]. Without loss of generality, we assume that $\beta >(u_0'')_{\max }$. Let $c<u_{0,\min }$. We define $f_0=(\psi _0+cy)\circ (\gamma _0+\beta y)^{-1}$ on $Ran(\gamma _0+\beta y)$, where $\psi _0$ and $\gamma _0$ are the stream function and vorticity of $(u_0,0)$. Then $f_0(\gamma _0+\beta y)=\psi _0+cy$ and $-f_0'(\gamma _0+\beta y)$ has uniform upper and lower positive bounds, that is,

$$\begin{aligned} 0<{u_{0,\min }-c\over \beta -(u_0'')_{\min }}\le -f_0'(\gamma _0+\beta y)={u_0-c\over \beta -u_0''}\le {u_{0,\max }-c\over \beta -(u_0'')_{\max }}. \end{aligned}$$

We now extend $f_0$ to $f\in C^1(\mathbb {R})$ such that $f=f_0$ on $Ran(\gamma _0+\beta y)$ and

5.47 $$\begin{aligned} 0<{1\over 2}{u_{0,\min }-c\over \beta -(u_0'')_{\min }}\le -f'\le {2(u_{0,\max }-c)\over \beta -(u_0'')_{\max }}. \end{aligned}$$

We choose *F* such that $F^{\prime }=f$. Define the energy-Casimir-momentum functional by

$$\begin{aligned} H(\gamma )=\int _{D_L}\left( -{1\over 2}|\vec {u}|^2-F(\gamma +\beta y)+cy(\gamma +\beta y)\right) dxdy \end{aligned}$$

along a perturbed solution $\vec {u}=(u,v)$ of the β-plane equation. Here, $\gamma =\partial _x v-\partial _y u=\Delta \psi$ and the boundary conditions are $\psi |_{y=\pm d}= B_{\pm }$ (with constants only depending on time). Without loss of generality, we can take the circulation (which is conserved) of the perturbed solution ψ satisfying

5.48 $$\begin{aligned} \int _{\mathbb {T}_L}\partial _y\psi (x,\pm d)dx=\int _{\mathbb {T}_L}\psi _0'(\pm d)dx=-Lu_0(\pm d) \end{aligned}$$

(i.e. the circulation of the perturbed solution is the same as that of the background shear flow). Indeed, if the circulation of the perturbed solution is different from that of the shear flow, then we can first modify the shear flow to a nearby shear flow such that the circulation of the modified shear flow is the same as that of the perturbed solution. Then, by an argument analogous to the one used below for nonlinear stability, we can show that the perturbed solution remains $L^2$-close (in vorticity) to the modified shear flow for all t>0. Since the modified shear flow is $L^2$-close (in vorticity) to the background shear flow, we conclude that the perturbed solution remains $L^2$-close (in vorticity) to the background shear flow for all times t>0.

Note that the functional H(γ) is conserved along the solution $\vec {u}=(u,v)$. In fact, by (2.1) we have

$$\begin{aligned} {d\over dt}{1\over 2}\int _{D_L}|\vec {u}|^2dxdy=&\, -\int _{D_L}\vec {u}\cdot ((\vec {u}\cdot \nabla )\vec {u}+\nabla P+\beta yJ\vec {u})dxdy\\ =&\, -\int _{D_L}\left( \vec {u}\cdot \nabla \left( {|\vec {u}|^2\over 2}\right) +\vec {u}\cdot \nabla P+\beta y\vec {u}\cdot J\vec {u}\right) dxdy\\ =&\, \int _{D_L}\left( (\nabla \cdot \vec {u}){|\vec {u}|^2\over 2}+(\nabla \cdot \vec {u}) P\right) dxdy=0, \end{aligned}$$

where we have used $\nabla \cdot \vec {u}=0$ and $\vec {u}\cdot J\vec {u}=0$. Thus, the kinetic energy ${1\over 2}\int _{D_L}|\vec {u}|^2dxdy$ is conserved along the solution $\vec {u}$.

Let (*X*(*s*; *x*, *y*), *Y*(*s*; *x*, *y*)) be the particle trajectory along the velocity $\vec {u}$, that is, (*X*(*s*; *x*, *y*),  *Y*(*s*; *x*, *y*)) solves the equation

$$\begin{aligned} {\left\{ \begin{array}{ll} \dot{X}(s)=u(s,X(s), Y(s)),\\ \dot{Y}(s)=v(s,X(s), Y(s)), \end{array}\right. } \end{aligned}$$

with the initial data X(0)=x,Y(0)=y. Note that (2.4) expresses the conservation of total vorticity along a fluid trajectory and the flow map (*X*, *Y*) is area preserving, that is,

$$\begin{aligned} {d\over dt}(\gamma (t,X(t),Y(t))+\beta Y(t))=0\quad \text {and} \quad {\partial {(X,Y)}\over \partial {(x,y)}}=1. \end{aligned}$$

Then

$$\begin{aligned}&{d\over dt}\int _{D_L}F(\gamma +\beta y)dxdy\\&\quad =\int _{D_L}F'(\gamma (t,X(t),Y(t))+\beta Y(t)){d\over dt}(\gamma (t,X(t),Y(t))+\beta Y(t))dXdY=0\,, \end{aligned}$$

showing that the Casimir functional $\int _{D_L}F(\gamma +\beta y) dxdy$ is conserved along the solution $\vec {u}$.

By (2.3) we have

5.49 $$\begin{aligned}&{d\over dt}\int _{D_L}y(\gamma +\beta y)dxdy\nonumber \\&\quad =\int _{D_L}y\partial _{t}(\gamma +\beta y)dxdy\nonumber \\&\quad =\int _{D_L}-\left( y(\vec {u}\cdot \nabla )\gamma +y\beta \partial _{x}\psi \right) dxdy\nonumber \\&\quad =\int _{D_L}-y\nabla \cdot (\vec {u}\gamma ) dxdy-\int _{-d}^dy\beta \psi \bigg |_{x=0}^{L}dy\nonumber \\&\quad =\int _{D_L}-y\partial _{y}(v\gamma )dxdy, \end{aligned}$$

where we have used the fact that both γ and ψ are periodic in *x*. Then (5.49) becomes

$$\begin{aligned}&{d\over dt}\int _{D_L}y(\gamma +\beta y)dxdy=-\int _{\mathbb {T}_L}y(v\gamma )\bigg |_{y=-d}^{d}dx+\int _{\mathbb {T}_L}\int _{-d}^dv\gamma dydx\\&\quad =\int _{D_L}\partial _{x}\psi (\partial _{xx}\psi +\partial _{yy}\psi )dydx\\&\quad =\int _{D_L}\frac{1}{2}\partial _{x}(\partial _{x}\psi )^2dydx -\int _{D_L}\frac{1}{2}\partial _{x}(\partial _{y}\psi )^2dydx=0, \end{aligned}$$

where we have used v(x,d)=v(x,-d)=0. Thus, the momentum functional $\int _{D_L}cy(\gamma +\beta y) dxdy$ is conserved along the solution $\vec {u}$. This proves that H(γ) is conserved along the solution $\vec {u}$.

Moreover, the functional derivative $H'(\gamma _0)=\psi _0+cy-f(\gamma _0+\beta y)$ is identically zero, since *H* is a functional of the vorticity. By $\psi |_{y=\pm d}= B_{\pm }$ and (5.48), we have

$$\begin{aligned}&\int _{D_L}\left( -{1\over 2}|\vec {u}|^2+{1\over 2}|\vec {u}_0|^2\right) dxdy\\&\quad ={1\over 2}\int _{D_L}\left( \psi \gamma -\psi _0\gamma _0\right) dxdy\\&\qquad -{1\over 2}\left( \psi |_{y= d}\int _{\mathbb {T}_L}\psi _y(x,d) dx-\psi |_{y=-d}\int _{\mathbb {T}_L}\psi _y(x,-d)dx\right) \\&\qquad +{1\over 2}\left( \psi _0(d)\int _{\mathbb {T}_L}\psi _{0y}(d)dx -\psi _0(-d)\int _{\mathbb {T}_L}\psi _{0y}(-d)dx\right) \\&\quad ={1\over 2}\int _{D_L}\left( \psi \gamma -\psi _0\gamma _0\right) dxdy -{1\over 2}\int _{D_L}\psi \gamma _0dxdy+\bigg ({1\over 2}\int _{D_L}\psi \gamma _0dxdy\\&\qquad -{1\over 2}\left( \psi |_{y= d}\int _{\mathbb {T}_L}\psi _{0y}(d) dx-\psi |_{y=-d}\int _{\mathbb {T}_L}\psi _{0y}(-d)dx\right) \\&\qquad +{1\over 2}\left( \psi _0(d)\int _{\mathbb {T}_L}\psi _{y}(x,d)dx -\psi _0(-d)\int _{\mathbb {T}_L}\psi _{y}(x,-d)dx\right) \bigg )\\&\quad ={1\over 2}\int _{D_L}\left( \psi \gamma -\psi _0\gamma _0\right) dxdy -{1\over 2}\int _{D_L}\psi \gamma _0dxdy+{1\over 2}\int _{D_L}\psi _0\gamma dxdy\\&\quad =\int _{D_L}\left( {1\over 2}(\psi -\psi _0)(\gamma -\gamma _0)+ \psi _0(\gamma -\gamma _0)\right) dxdy \end{aligned}$$

and thus

$$\begin{aligned}&H(\gamma )-H(\gamma _0)\\&\quad =\int _{D_L}\left( -{1\over 2}|\vec {u}|^2+{1\over 2}|\vec {u}_0|^2-(F(\gamma +\beta y)-F(\gamma _0+\beta y))+cy(\gamma -\gamma _0)\right) dxdy\\&\quad =\int _{D_L}\bigg ({1\over 2}(\psi -\psi _0)(\gamma -\gamma _0)+(\psi _0+cy)(\gamma -\gamma _0)\\&\qquad -(F(\gamma +\beta y)-F(\gamma _0+\beta y))\bigg )dxdy\\&\quad =\int _{D_L}-{1\over 2}|\nabla (\psi -\psi _0)|^2dxdy\\&\qquad +\int _{D_L} \bigg (-(F(\gamma +\beta y)-F(\gamma _0+\beta y)-F'(\gamma _0+\beta y)(\gamma -\gamma _0))\bigg )dxdy\\&\quad =I+II. \end{aligned}$$

By $-F^{\prime \prime }=-f^{\prime }$ and (5.47), we have

$$\begin{aligned} {1\over 4}{u_{0,\min }-c\over \beta -(u_0'')_{\min }}\Vert \gamma -\gamma _0\Vert _{L^2(D_L)}^2\le II\le {u_{0,\max }-c\over \beta -(u_0'')_{\max }}\Vert \gamma -\gamma _0\Vert _{L^2(D_L)}^2. \end{aligned}$$

By the Poincaré inequality, we have $\Vert \nabla (\psi -\psi _0)\Vert _{L^2(D_L)}^2=-\int _{D_L}(\psi -\psi _0)(\gamma -\gamma _0)dxdy\le C_0 \Vert \nabla (\psi -\psi _0)\Vert _{L^2(D_L)}\Vert \gamma -\gamma _0\Vert _{L^2(D_L)}$, and thus $\Vert \nabla (\psi -\psi _0)\Vert _{L^2(D_L)}\le C_0 \Vert \gamma -\gamma _0\Vert _{L^2(D_L)}$. Then

$$\begin{aligned} I\ge -{1\over 2} C_0^2\Vert \gamma -\gamma _0\Vert _{L^2(D_L)}^2. \end{aligned}$$

Choose $c<u_{0,\min }$ such that

$$\begin{aligned} {u_{0,\min }-c\over 2(\beta -(u_0'')_{\min })}>C_0^2. \end{aligned}$$

The estimates for *I* and *II* yield

$$\begin{aligned} {u_{0,\max }-c\over \beta -(u_0'')_{\max }}\Vert \gamma (0)-\gamma _0\Vert _{L^2(D_L)}^2\ge &   H(\gamma (0))-H(\gamma _0)=H(\gamma (t))-H(\gamma _0)\\\ge &   {1\over 2}\left( {u_{0,\min }-c\over 2(\beta -(u_0'')_{\min })}- C_0^2\right) \Vert \gamma (t)-\gamma _0\Vert _{L^2(D_L)}^2. \end{aligned}$$

This proves that

$$\begin{aligned}&\Vert \gamma (t)-\gamma _0\Vert _{L^2(D_L)}^2 \\&\quad \le {{2(u_{0,\max }-c)/ (\beta -(u_0'')_{\max })} \over {(u_{0,\min }-c)/ (2\beta -2(u_0'')_{\min })}- C_0^2}\Vert \gamma (0)-\gamma _0\Vert _{L^2(D_L)}^2\,,\qquad t >0\,. \end{aligned}$$

By Theorem 5.10, genuine traveling waves exist near the monotone shear flow $(u_0,0)$ in $C^2$, and thus nonlinear inviscid damping near $(u_0,0)$ in $C^2$ fails. □

## Applications to the Couette–Poiseuille flow, to the Bickley Jet and to the Kolmogorov Flow

In this section, building on the results of Sect. 3, we investigate the rigidity of traveling waves near non-monotone shear flows which include the Couette–Poiseuille flow and the Bickley jet in a bounded channel. Moreover, we study the rigidity of traveling waves with a fixed wave speed near a class of shear flows and describe the inviscid dynamical structures near the Kolmogorov flow in the *f*-plane setting.

### Rigidity Near a Class of Shear Flows with Applications

Based on Theorem 3.12, we establish a rigidity result for geophysical traveling waves with arbitrary speeds near a class of general shear flows. Then we apply this result to the Couette–Poiseuille flow and the Bickley jet.

#### A General Rigidity Result

We first present a rigidity criterion near a general class of shear flows.

##### Proposition 6.1

Assume that $u_0\in C^2([-d,d])$, $(u_0'')_{\min }>0$ and $\beta \in \left[ 0,(u_0'')_{\min }\right)$. If $(u(x-ct,y),v(x-ct,y))\in C^2(D_L)$ is a traveling-wave solution to the β-plane equation (2.1)-(2.2) with $c\in \mathbb {R}$, and satisfies

6.1 $$\begin{aligned} \Vert \Delta u-u_{ 0}''\Vert _{C^0(D_L)}<(u_0'')_{\min }-\beta , \end{aligned}$$

then (u(x-ct,y),v(x-ct,y)) is a shear flow.

##### Proof

It follows from (6.1) that

$$\begin{aligned} |(\Delta u)(x,y)-u_{0}''(y)| <(u_{0}'')_{\min }-\beta ,\quad (x,y)\in D_L. \end{aligned}$$

Thus

$$\begin{aligned} \beta -(u_{0}'')_{\min }<(\Delta u)(x,y)-u_{0}''(y)\le (\Delta u)(x,y)-(u_{0}'')_{\min },\quad (x,y)\in D_{L}. \end{aligned}$$

Hence $0\le \beta <(\Delta u)_{\min }$. By Theorem 3.12, (u(x-ct,y),v(x-ct,y)) is a shear flow. □

##### Example 6.2

Saturn’s southern polar troposphere features at cloud level a strongly sheared circumpolar jet flowing eastward in the zonal band between 65$^\circ$S and 70$^\circ$S. The jet profile exhibits a nearly flat slope at 65$^\circ$S, with a poleward speed increasing from about $0\,\text {m}\,\text {s}^{-1}$ at 65$^\circ$S to a maximal speed of $100\,\text {m}\,\text {s}^{-1}$ at 70$^\circ$S (see Fig. 12.5 in [42]). With the scaling parameters provided in Table 1, we obtain the boundary values $u_0(-d)={2\over 3}$, $u_0(d)=0$ and $u_0'(d)=0$. Consequently, we may model this sheared, purely zonal flow by the parabolic profile

$$\begin{aligned} u_0(y)={1\over 6d^2}y^2-{1\over 3d}y+{1\over 6},\quad -d\le y\le d, \end{aligned}$$

in a band of width $2d={\pi \over 36}\approx 0.0873$ corresponding to about 5 degrees of latitude. Using the data in Table 1, we infer from (1.1) that

$$\begin{aligned} \beta ={2\times 1.62\times 10^{-4}\times 58232\times 10^3\over 150}\cos (67.5^\circ )\approx 48<(u_0'')_{\min }={1\over 3d^2}\approx 175 \end{aligned}$$

so that Proposition 6.1 predicts the absence of genuine traveling waves near this zonal flow. This conclusion is validated by the field data (see [42]).

#### Application to the Couette–Poiseuille Flow

For $\Gamma \in \mathbb {R}$, the generalized Couette–Poiseuille flow is given by

6.2 $$\begin{aligned} u_\textrm{cp}(y)=\Gamma y+(1-\Gamma )y^2, \quad y\in [-1,1]\,. \end{aligned}$$

##### Corollary 6.3

Assume that Γ<1 and $\beta \in \left[ 0,2-2\Gamma \right)$. If $(u(x-ct,y),v(x-ct,y))\in C^2(D_L)$ is a traveling-wave solution to the β-plane equation (2.1)-(2.2) with $c\in \mathbb {R}$ and such that

6.3 $$\begin{aligned} \Vert \Delta u-u_\textrm{cp}''\Vert _{C^0(D_L)}<2-2\Gamma -\beta , \end{aligned}$$

then (u(x-ct,y),v(x-ct,y)) is a shear flow in $D_L=\mathbb {T}_L\times [-d,d]$ with d=1.

##### Proof

Since Γ<1, we have $u_\textrm{cp}''\equiv 2-2\Gamma >0$. The result therefore follows immediately from Proposition 6.1. □

##### Remark 6.4

For the Poiseuille flow, corresponding to Γ=0 in (6.2), in the *f*-plane setting, Corollary 6.3 complements the results in [13, 15]. Indeed, the rigidity of traveling waves with arbitrary wave speeds was obtained in [13] under the high regularity assumption (velocity) $H^{>6}$, and Corollary 6.3 lowers the regularity to $C^2$. In addition, although the rigidity of steady flows near the Poiseuille flow was proved in $C^2$ (see [15]), Corollary 6.3 further extends the conclusion to encompass all traveling wave speeds. See also the recent independent result for β=0 in Corollary 1.6(i) of [18].

#### Application to the Bickley Jet

The profile of a bounded Bickley jet is

$$\begin{aligned} u_\textrm{b}(y)=-\operatorname {sech}^{2}(y),\quad y\in [-d,d]. \end{aligned}$$

As another application of Proposition 6.1, we investigate the rigidity of traveling waves with arbitrary speeds near the Bickley jet.

##### Corollary 6.5

Assume that $d\in \left( 0,\frac{\ln (2+\sqrt{3})}{2}\right)$ and $\beta \in \left[ 0,2\operatorname {sech}^{4}(d)-4\operatorname {sech}^{2}\right. \left. (d)\operatorname {tanh}^{2}(d)\right)$. If $(u(x-ct,y),v(x-ct,y))\in C^2(D_L)$ is a traveling-wave solution to the β-plane equation (2.1)-(2.2) with $c\in \mathbb {R}$, and satisfies

6.4 $$\begin{aligned} \Vert \Delta u-u_\textrm{b}''\Vert _{C^0(D_L)}<2\operatorname {sech}^{4}(d)-4\operatorname {sech}^{2}(d)\operatorname {tanh}^{2}(d)-\beta , \end{aligned}$$

then (u(x-ct,y),v(x-ct,y)) is a shear flow.

##### Remark 6.6

The real zeros of $u_\textrm{b}''(y)=2\operatorname {sech}^{4}(y)-4\operatorname {sech}^{2}(y)\operatorname {tanh}^{2}(y)=0$ are $\frac{\ln (2+\sqrt{3})}{2}$ and $\frac{\ln (2-\sqrt{3})}{2}$. Moreover, $u_\textrm{b}''>0$ on $\left( \frac{\ln (2-\sqrt{3})}{2},\frac{\ln (2+\sqrt{3})}{2}\right)$. If $d\in \left( 0,\frac{\ln (2+\sqrt{3})}{2}\right)$, then

6.5 $$\begin{aligned} 2\operatorname {sech}^{4}(d)-4\operatorname {sech}^{2}(d)\operatorname {tanh}^{2}(d)=u_\textrm{b}''(\pm d)=(u_\textrm{b}'')_{\min }>0. \end{aligned}$$

Thus the range of β in Corollary 6.5 is non-null.

##### Proof

By (6.5), we have $(u_\textrm{b}'')_{\min }= 2\operatorname {sech}^{4}(d)-4\operatorname {sech}^{2}(d)\operatorname {tanh}^{2}(d)>0$. The conclusion follows directly from Proposition 6.1. □

### Application to the *f*-Plane Setting

In this subsection, as an application of Theorem 3.3, we study the rigidity of traveling waves in the *f*-plane setting. Moreover, we prove some rigidity results for traveling waves with non-vanishing speeds near a Kolmogorov flow in the *f*-plane setting. On the other hand, we construct genuine analytic traveling waves with vanishing speed (i.e. steady flow) near the Kolmogorov flow.

#### *Rigidity of Traveling Waves in the f-Plane Setting*

The following rigidity result in the *f*-plane setting is a consequence of Theorem 3.3.

##### Corollary 6.7

Let β=0 and $c\in \mathbb {R}$. Assume that $\Delta u\ne 0$ whenever u=c. If $(u(x-ct,y),v(x-ct,y))\in C^2(D_L)$ is a traveling-wave solution to the β-plane equation (2.1)-(2.2), then (u(x-ct,y),v(x-ct,y)) is a shear flow.

##### Remark 6.8

In the *f*-plane setting, by employing a different approach, Corollary 6.7 generalizes Corollary 1.6 $(\textrm{ii})$ in [18] (for the case of a bounded periodic channel) and Theorem 1.2 in [15] in two aspects: one is by generalizing steady flows to traveling waves with non-vanishing wave speeds, and the other aspect is that the traveling waves are not necessarily near a shear flow. See Proposition 6.9 for the rigidity of traveling waves with a fixed wave speed near a shear flow.

As an application of Corollary 6.7, we establish the rigidity of traveling waves with a fixed wave speed near a class of shear flows in the *f*-plane setting.

##### Proposition 6.9

Let β=0. Assume that $u_0\in C^2([-d,d])$ and $c\in \mathbb {R}$ satisfy $u_0''\ne 0$ whenever $u_0-c=0$. Then there exists $\varepsilon _0>0$ such that if $(u(x-ct,y),v(x-ct,y))\in C^2(D_L)$ is a traveling-wave solution to the β-plane equation (2.1)-(2.2) and satisfies

$$\begin{aligned} \Vert u-u_{0}\Vert _{C^2(D_L)}<\varepsilon _0, \end{aligned}$$

then (u(x-ct,y),v(x-ct,y)) has to be a shear flow.

##### Proof

If u=c has no zeros, then (u(x-ct,y),v(x-ct,y)) is a shear flow by Theorem 1.1 in [31]. If u=c has zeros, we choose an arbitrary zero $(x_0,y_0)$. We will show that

6.6 $$\begin{aligned} (\Delta u)(x_0,y_0)\ne 0. \end{aligned}$$

Since $u_0\in C^2 ([-d,d])$ and $u_0''\ne 0$ whenever $u_0-c=0$, the set $\{y:u_0-c=0\}$ is finite and is denoted by $\{y_i:1\le i\le n\}$. Choose $\delta _0>0$ such that $(y_i-\delta _0,y_i+\delta _0)\cap [-d,d]$, $1\le i\le n$, are disjoint, and

6.7 $$\begin{aligned} |u_0''|>{\min _{1\le i\le n}|u_0''(y_i)|\over 2}>0\quad \text {on}\quad U_0=\cup _{1\le i\le n}((y_i-\delta _0,y_i+\delta _0)\cap [-d,d]).\end{aligned}$$

Moreover, $$\min _{y\in [-d,d]\setminus U_0}|u_0(y)-c|>0$$. Let

$$\begin{aligned} \varepsilon _0=\min \left\{ {\min _{1\le i\le n}|u_0''(y_i)|\over 2},{\min _{y\in [-d,d]\setminus U_0}|u_0(y)-c|\over 2}\right\} >0. \end{aligned}$$

Since $\Vert u-u_0\Vert _{C^2(D_L)}<\varepsilon _0$, we have

$$\begin{aligned}&|u(x,y)-c|\ge |u_0(y)-c|-|u_0(y)-u(x,y)|\\&\quad>\min _{y\in [-d,d]\setminus U_0}|u_0(y)-c|-{\min _{y\in [-d,d]\setminus U_0}|u_0(y)-c|\over 2}\\&\quad ={\min _{y\in [-d,d]\setminus U_0}|u_0(y)-c|\over 2}>0 \end{aligned}$$

for $(x,y)\in \mathbb {T}_L\times ([-d,d]\setminus U_0)$. Thus, u-c≠0 on $\mathbb {T}_L\times ([-d,d]\setminus U_0)$, which implies $(x_0,y_0)\in \mathbb {T}_L\times U_0$. Therefore, by (6.7) and $\Vert u-u_0\Vert _{C^2(D_L)}<\varepsilon _0$ we have

$$\begin{aligned} |(\Delta u)(x_0,y_0)|\ge |u_0''(y_0)|-|u_0''(y_0)-(\Delta u)(x_0,y_0)|>{\min _{1\le i\le n}|u_0''(y_i)|\over 2}-\varepsilon _0\ge 0. \end{aligned}$$

This proves (6.6). Applying Corollary 6.7, (u(x-ct,y),v(x-ct,y)) is a shear flow. □

The following remark about the *f*-plane setting explains how Theorem 3.3 (ii) complements the rigidity result established in [20]:

##### Remark 6.10

In the *f*-plane setting, let $(u(x-ct,y),v(x-ct,y))\in C^2(D_L)$ be a traveling-wave solution to (2.1)-(2.2). Then (u-c,v) is a steady solution of (2.1)-(2.2). Theorem 3.3 (ii) can be viewed as a rigidity result in the presence of stagnation points of (u-c,v), see (3.10). This stands in contrast to the rigidity result by Hamel-Nadirashvili [20], which assumes that (u-c,v) has no stagnation points throughout the domain. Our result therefore complements Hamel-Nadirashvili’s by addressing a regime that was previously excluded. The combination of Theorem 3.3 (ii) and Hamel-Nadirashvili’s result can be stated as “if *c* is not a generalized inflection value of *u* or if (u-c,v) has no stagnation points, then (u(x-ct,y),v(x-ct,y)) is a shear flow in the *f*-plane setting."

#### Application to Inviscid Dynamical Structures Near the Kolmogorov Flow in the *f*-Plane Setting

In the finite channel $(x,y)\in D_{L_0}=\mathbb {T}_{L_0}\times [-d,d]$ with $L_0={4\pi \over \sqrt{3}}$ and d=π, we consider the Kolmogorov flow

6.8 $$\begin{aligned} u_\textrm{k}(y)=\sin (y),\quad y\in [-\pi ,\pi ]\,, \end{aligned}$$

and we study the inviscid dynamical structures near it in the *f*-plane setting.

##### Proposition 6.11

Let β=0.

(i) For any δ>0, if a traveling-wave solution $(u(x-ct,y),v(x-ct,y))\in C^2(D_{L_0})$ to the β-plane equation (2.1)-(2.2) satisfies that $c\in (-\infty ,-\delta ]\cup [\delta ,\infty )$ and if
  6.9 $$\begin{aligned} \Vert u-u_\textrm{k}\Vert _{C^2(D_{L_0})}<\delta , \end{aligned}$$
  then (u(x-ct,y),v(x-ct,y)) is a shear flow.
(ii) For any $\varepsilon \in \mathbb {R}$, $(u_\varepsilon ,v_\varepsilon )$ in (3.30) is a non-sheared steady solution to the β-plane equation (2.1)-(2.2). In particular, for ε small enough, the non-sheared steady flow $(u_\varepsilon ,v_\varepsilon )$ is sufficiently close in analytic regularity to the Kolmogorov flow (6.8).

##### Proof

(i) If c∉Ran(u), then (u(x-ct,y),v(x-ct,y)) is a shear flow by Theorem 1.1 in [31]. Thus we only need to consider the case c∈Ran(u). For any $(x_c,y_c)\in D_{L_0}$ satisfying $u(x_c,y_c)=c$, by (6.9) we get

$$\begin{aligned} |(\Delta u)(x_c,y_c)|&=|c-(c-u_\textrm{k}(y_c))-\left( u_\textrm{k}(y_c)+(\Delta u)(x_c,y_c)\right) |\\&\ge |c|-|c-u_\textrm{k}(y_c)|-|u_\textrm{k}(y_c)+(\Delta u)(x_c,y_c)|\\&\ge |c|-|u(x_c,y_c)-u_\textrm{k}(y_c)|-|-u_\textrm{k}''(y_c)+(\Delta u)(x_c,y_c)|\\&\ge |c|-\Vert u-u_\textrm{k}\Vert _{C^2(D_{L_0})}\\&>\delta -\delta =0, \end{aligned}$$

where we have used $u_\textrm{k}(y_c)=-u_\textrm{k}''(y_c)$ and $c\in (-\infty ,-\delta ]\cup [\delta ,\infty )$. Then (u(x-ct,y),v(x-ct,y)) is a shear flow by Corollary 6.7.

(ii) The stream function and vorticity of $(u_\varepsilon ,v_\varepsilon )$ in (3.30) are

$$\begin{aligned} \psi _\varepsilon (x,y)&=\cos (y)+\varepsilon \cos \left( {y\over 2}\right) \sin \left( {\sqrt{3}\over 2}x\right) \quad \text {and}\\ \gamma _\varepsilon (x,y)&=-\cos (y)-\varepsilon \cos \left( {y\over 2}\right) \sin \left( {\sqrt{3}\over 2}x\right) . \end{aligned}$$

Since $\{\psi _\varepsilon ,\gamma _\varepsilon \}=0$ on $D_{L_0}$ and

$$\begin{aligned} v_\varepsilon =0\quad \text {on}\quad y=\pm \pi , \end{aligned}$$

$(u_\varepsilon ,v_\varepsilon )$ is a non-sheared steady solution to the β-plane equation (2.1)-(2.2) with β=0. Moreover, by its expression in (3.30), $(u_\varepsilon ,v_\varepsilon )$ is sufficiently close in analytic regularity to the Kolmogorov flow (6.8) as long as ε is taken small enough. □

## Data Availability

All data for this paper are properly cited and referred to the relevant data that can be found in [12, 17, 19, 26, 39, 41–43, 45–47].
